# Individual differences and motives for the acceptance of cognitive enhancement: A mixed-methods investigation

**DOI:** 10.1371/journal.pone.0353234

**Published:** 2026-07-10

**Authors:** Sandra Grinschgl, Larissa Kohlmeier, Aljoscha C. Neubauer

**Affiliations:** 1 Institute of Psychology, University of Graz, Graz, Austria; 2 Institute of Psychology, University of Bern, Bern, Switzerland; University of Padova, ITALY

## Abstract

Despite a long history of human efforts to enhance cognitive abilities such as attention, memory, and intelligence, the proliferation of (new) technological and medical methods—including brain stimulation, pharmaceutical interventions, and gamified brain training—has renewed scientific and public interest in cognitive enhancement, even as evidence for their effectiveness in improving broader cognitive abilities remains inconsistent. Understanding who would be willing to enhance their cognitive abilities and why is crucial as these cognitive enhancement methods become more popular and accessible in society. Thus, in two preregistered lab studies, we investigated individuals’ acceptance (i.e., hypothetical willingness to make use) of various cognitive enhancement methods, tested predictors thereof, and explored individuals’ motives to utilize cognitive enhancement methods or be reluctant to do so. In Study 1 (*N* = 203), we found considerably higher acceptance for more active (i.e., more involved) than passive cognitive enhancement methods. Lower age, greater interest in science fiction, and stronger investigative interests (partially) predicted participants’ acceptance of cognitive enhancement methods, while personality traits, self-assessed and measured intelligence did not. In Study 2 (*N* = 197), we aimed to (conceptually) replicate key findings from Study 1, and in addition qualitatively assess the motives for the acceptance and rejection of cognitive enhancement methods. Our findings confirmed the higher acceptance of active than passive cognitive enhancement methods. Additionally, we found that greater interest in science fiction consistently predicted participants’ acceptance of cognitive enhancement, while personality traits and (self-assessed) intelligence did not show consistent associations. Critically, these quantitative effects were rather small. Qualitative analyses additionally provided exploratory insights into the motives for accepting or rejecting active and passive cognitive enhancement methods. Across these two studies, acceptance of cognitive enhancement seems to be more closely linked to specific interests than to personality traits or (self-assessed) abilities. We emphasize the importance of studying situational factors, such as person-environment fit, to better understand the willingness to make use of cognitive enhancement.

## 1. Introduction

Questions about how far we can—and should—improve human cognition have long fascinated both science and society. From the idea of memory-enhancing techniques to visions of ‘Homo superior’ in literature and film, the prospect of going beyond natural limits has sparked curiosity, excitement, and controversy. Although people have pursued forms of cognitive enhancement for decades (e.g., the use of stimulants as “study drugs” in the 1930s) or even longer (e.g., our ancestors chewing coca leaves for alertness; see [1] for a summary), new, radical forms of cognitive enhancement—such as direct modifications of the brain via brain-computer interfaces—have only recently been targeted in empirical research and public debates. Especially in today’s fast-paced society, an enhancement of our cognitive abilities seems to be of high interest. Given the rapid development of AI (artificial intelligence) technologies and the rising fears surrounding the loss of many traditional jobs [2]), discussions about the necessity of cognitive enhancement seem omnipresent. As Corneliu E. Giurgea, an early advocate of cognitive enhancement, stated, “Man is not going to wait passively for millions of years before evolution offers him a better brain” [3] (translated in [4, p. 379]). In the present set of studies, we investigated the acceptance of different cognitive enhancement methods (i.e., whether individuals would—hypothetically—be willing to make use of them) and wanted to know whether predictors such as intelligence, personality traits, and interests might be contributing to cognitive enhancement acceptance. Furthermore, we qualitatively assessed motives to apply cognitive enhancement.

### 1.1. Cognitive enhancement methods

Certain philosophical and political movements such as Transhumanism strongly advocate cognitive enhancement methods. Transhumanists propose to apply technological and medical innovations to improve humans’ characteristics, such as cognitive abilities, creativity, or personality traits [5]. Importantly, this enhancement is aimed at healthy individuals and, thus, can be distinguished from compensational methods to treat, for instance, diseases [6]. According to Transhumanism, enhanced humans should overcome biological limitations and be “smart enough” to resolve global issues, such as the climate crisis [7,8].

While there are more and more public debates about cognitive enhancement (e.g., see longevity project of Bryan Johnson or the Huberman Lab Podcast by Andrew Huberman), the public’s perception on cognitive enhancement might vary depending on the enhancement method. In a recent study, Grinschgl et al. [9] investigated the acceptance of different cognitive enhancement methods and observed a substantially higher acceptance of *active* cognitive enhancement methods than *passive* methods*.* Active and passive cognitive enhancement methods differ in the required level of agency–with higher agency for active cognitive enhancement methods—and the perceived risk–with higher risk being associated with passive cognitive enhancement methods [6,9]. Most active and passive cognitive enhancement methods, however, currently demonstrate no or only low efficacy (for a comprehensive review see [6]), thus, at least in (healthy) adults a substantial improvement of cognitive abilities does not seem to be possible at this moment.

### 1.2. Active cognitive enhancement methods

Active cognitive enhancement methods are characterized by the need to invest resources, like time and energy, to achieve an (supposed) improvement of cognitive abilities [9]. For instance, working memory or neurofeedback training requires active exercising over multiple weeks or even months.

**Working memory training.** Working memory training is offered by numerous apps and websites, most commonly through working memory tasks such as the n-back or complex span tasks (e.g., BrainScale [10]).

**Game-based enhancement.** Game-based enhancement refers to the improvement of cognitive abilities using video games, e.g., incorporating game elements into non-gaming, training contexts (gamification, see [11]). There is a substantial market for game-based cognitive enhancement methods, as evidenced by the popularity of apps like *Elevate* [12] and *Lumosity* [13], each with over 50 million users [14,15], and newer VR-based approaches, such as *Enhance VR* from *Virtuleap* [16].

**Neurofeedback training.** Neurofeedback training enables individuals to learn to actively control their brain activity using direct visual, auditory or haptic feedback based on electroencephalography (EEG) or functional magnetic resonance imaging (fMRI) signals [17]. While being primarily used in a clinical context (e.g., for treating psychiatric disorders [18]), consumer-grade neurofeedback devices are already available (e.g., the Crown from Neurosity [19]).

### 1.3. Passive cognitive enhancement methods

Unlike active cognitive enhancement methods, passive methods—such as a simple intake of a smart drug—require less agency and a smaller investment of time and energy to (presumably) benefit from an increase in cognitive performance.

**Pharmacological enhancement.** Pharmacological enhancement refers to the (ab)use of pharmacological substances, also often referred to as smart drugs, with the goal to increase cognitive abilities. Review papers show that prevalence estimates—especially among university students—differ substantially (e.g., between 2.3 and 55% [20]). While some sources suggest arising use of pharmaceutical enhancement [21,22], others suggest a decline (see [23] for a summary).

**Current based enhancement.** Non-invasive forms of current based enhancement refer to the application of weak electrical currents to the scalp, modulating the electrical activity in the brain, presumably impacting cognitive performance. Consumer-grade devices are already available—such as the LIFTiD Neurostimulation [24]—and there is a growing community of do-it-yourself tDCS users [25].

**Invasive brain machine interfaces.** Brain-machine interfaces (BMIs), or brain-computer interfaces (BCIs), enable direct communication between the brain and external devices, usually through surgically implanted electrodes. While primarily studied for therapeutic uses (e.g., prosthetic control [26] or communication in locked-in patients [27]), the potential of invasive BMIs for cognitive enhancement in healthy individuals is also discussed, notably by Elon Musk’s company Neuralink [28].

**Genetic enhancement.** Genetic enhancement, also referred to as gene editing, allows the modification of genes and thereby physiological, psychological or cognitive characteristics in, for instance, a fetus [29]. Gene editing is mostly investigated in regard to a therapeutic application (e.g., to cure genetic diseases [30]) and gene-edited babies have been born [31]. To our best knowledge, gene editing has not yet been specifically tested for cognitive enhancement, but heavily discussed, for instant with regard to “designer babies”.

### 1.4. The acceptance of cognitive enhancement methods

Previous research has mostly examined how people evaluate cognitive enhancement more broadly, focusing on general attitudes and the arguments individuals raise in favor of or against such methods. For example, Dijkstra and Schuijff [32] found that arguments in favor of cognitive enhancement often emphasized individual benefits, while oppositions were typically rooted in concerns about societal consequences. Similarly, Grinschgl et al. [33] observed that if consequences of cognitive enhancement are more targeting the society, they are more negative. In return, consequences targeting the individual are more positive. Concerns about fairness, coercion, and medical safety have also been highlighted, particularly in the context of pharmacological enhancement [34]. Furthermore, studies suggest that cognitive enhancement methods perceived as more natural, less invasive, and less frequently applied are generally more accepted [35,36].

While these studies provide insights into general attitudes towards cognitive enhancement, relatively little is known about how individual differences—such as cognitive abilities, personality traits, and interests—are related to the acceptance of cognitive enhancement methods. In one of the few psychological studies, Laakasuo et al. [37] investigated a futuristic form of enhancement—mind uploading—and observed that high purity norms and sexual disgust sensitivity are linked to condemning this method. On the other hand, a younger age and high Science-Fiction hobbyism, but not personality, was linked to an acceptance of mind uploading. In a later study of Breivik et al. [38], certain personality traits such as openness and agreeableness were linked to the willingness to use pharmacological enhancement.

Beyond those investigations of single cognitive enhancement methods, Schönthaler et al. [39] investigated individual differences with regard to the acceptance of a range of different passive cognitive enhancement methods. In a hierarchical regression model, a lower industriousness (sub-facet of conscientiousness) and self-transcendence values, as well as higher self-enhancement values explained variance in the acceptance of passive cognitive enhancement methods, while other personality and Dark triad traits did not.

Grinschgl et al. [9] extended this study by including active and passive cognitive enhancement methods. In this online study, the authors observed that cognitive enhancement methods can—through factor analysis—be separated into active and passive methods, with the former showing a considerably higher acceptance than the latter (*d* = 1.30). Furthermore, they observed a small positive correlation between intelligence and the acceptance of active cognitive enhancement methods, but not the acceptance of passive methods. There was no link between self-estimated intelligence and the acceptance of either form of cognitive enhancement methods. With regard to personality, Grinschgl et al. [9] found a negative relationship of conscientiousness as well as a positive relationship of neuroticism with the acceptance of passive cognitive enhancement methods. For active cognitive enhancement methods, they observed a positive relationship with openness. The Dark triad traits Machiavellianism and grandiose narcissism were positively correlated with acceptance of both active and passive cognitive enhancement methods, while vulnerable narcissism was only related to the acceptance of passive cognitive enhancement methods. Purity norms were negatively correlated to the acceptance of active cognitive enhancement methods, while Science-Fiction hobbyism was positively related to both forms of cognitive enhancement. Most importantly, in hierarchical regressions models, for passive cognitive enhancement methods only a younger age, lower conscientiousness, and higher Science-Fiction hobbyism served as significant predictors. For active cognitive enhancement methods, a younger age, higher openness, higher Science-Fiction hobbyism and lower purity norms significantly predicted acceptance.

Following up on this study, in the present two studies we sought to replicate previous findings and investigate additional individual differences that might be related to the acceptance of both active and passive cognitive enhancement methods. First, we aimed at taking a closer look at intelligence and self-estimated intelligence, as cognitive enhancement methods explicitly target cognitive abilities, like intelligence. For this link, two hypotheses have been proposed [9,40]: the *rich-get-richer* hypothesis and the *compensation* hypothesis. The *rich-get-richer* hypothesis proposes that individuals with high cognitive abilities would benefit more from cognitive enhancement and might also be more drawn towards cognitive enhancement (see also ‘Matthew effect’ [41]). Their cognitive abilities would increase, further distinguishing them from individuals with lower cognitive abilities. The *compensation* hypothesis proposes the opposite effect. People with lower cognitive abilities would benefit more from cognitive enhancement and might be more drawn towards it, resulting in an equalizing effect. This would alter the distribution of intelligence in the population, rendering cognitive ability less important due to the reduced variance [40] (see also reverse Matthew effect [42]).

However, the decision to enhance one’s cognitive abilities might be made based on the subjective assessment of one’s own abilities, rather than one’s actual abilities. Thus, potentially self-estimated intelligence—not psychometrically measured intelligence—is related to the acceptance of cognitive enhancement methods. Similarly, Neubauer & Hofer [43] showed that for professional interests (potentially steering important life decisions like career choices), the self-estimates of abilities play a larger role than psychometric abilities themselves. While Grinschgl et al. [9] investigated these relationships of psychometrically measured and self-estimated intelligence with the acceptance of cognitive enhancement methods in an online study, we sought to investigate them in a lab-based assessment (as more appropriate for intelligence tests) in the present study.

As observed in previous studies [9,38,39], also certain personality traits might be related to the acceptance of cognitive enhancement. Thus, in Study 1 we investigated how the Big 5 traits are related to the acceptance of both active and passive cognitive enhancement methods. Especially, conscientiousness might be negatively related to the acceptance of—at least passive—cognitive enhancement methods, as observed previously [9,44,45]. To gain a more nuanced understanding of the relationship between conscientiousness and the acceptance of cognitive enhancement methods, we aimed to investigate the link between their acceptance and the six facets of conscientiousness (competence, order, dutifulness, achievement striving, self-discipline and deliberation [46]) in our second study. Potentially, while some facets of conscientiousness might be negatively related to the acceptance of cognitive enhancement methods, others might not show a relationship [39], or even a positive relationship (such as achievement striving).

Beyond the Big 5 personality traits, in Study 1 we also tested the relationships of the Dark triad traits and the acceptance of cognitive enhancement methods. While Machiavellianism and grandiose neuroticism might be positively related to the acceptance of active and passive cognitive enhancement methods as observed in Grinschgl et al. [9] in zero-order correlations, the third Dark triad trait psychopathy might not. We aimed at again testing a potential (lack of) relationship in the present studies.

In addition to personality, traits such as anxiety and novelty seeking might be related to the acceptance of cognitive enhancement methods. Research indicates that individuals with high levels of anxiety perform worse on complex cognitive tasks [47]. Thus, further rejection of cognitive enhancement could result in a *doubled disadvantage*, as proposed by Neubauer [40]. Anxiety may, however, influence the acceptance of cognitive enhancement methods in various ways: it is positively correlated with prescription drug abuse [48], which could affect the acceptance of pharmacological enhancement. Yet, it is also associated with higher risk avoidance [49], reduced treatment seeking [50], and a heightened fear of medical interventions [51] and technology [52], possibly suggesting a negative relationship between anxiety and the acceptance of (specifically passive) cognitive enhancement methods. To explore this potential relationship (and the direction thereof), we aimed to assess trait anxiety in relation to the acceptance of cognitive enhancement methods in our second study. Furthermore, in this study we also tested novelty seeking as a potential predictor of the acceptance of cognitive enhancement methods. A study from Maier et al. [53] found a positive relationship between novelty seeking and the use of pharmacological enhancement and people with high levels of novelty seeking may be early adopters of new technologies [54]. They further show a higher risk preference [55], and higher delay discounting (i.e., a higher preference for a smaller, immediate reward over a bigger, delayed rewards [56]), potentially indicating a positive relationship with the acceptance of (specifically passive) cognitive enhancement methods.

As additional—and so far untested—predictors of the acceptance of cognitive enhancement methods, in Study 1 we examined self-esteem and vocational interests as described by the RIASEC model [57]. Individuals with lower self-esteem might be more drawn toward cognitive enhancement, similar to those with lower self-estimated cognitive abilities. The six RIASEC dimensions capture broad domains of interest: realistic (practical, hands-on activities), investigative (scientific and analytical problem-solving), artistic (creative and expressive pursuits), social (helping and teaching others), enterprising (persuasive and leadership-oriented tasks), and conventional (structured, rule-based activities). Certain interests, such as investigative interests, which emphasize curiosity, analysis, and problem-solving—may be linked to acceptance of cognitive enhancement methods. This assumption builds on previous findings showing that technical interests and Science-Fiction hobbyism are associated with a higher acceptance of cognitive enhancement methods [9,39].

Relatedly, in both of our studies we investigated the relationship of Science-Fiction hobbyism and the acceptance of cognitive enhancement methods. Thereby, we aimed to replicate previous findings from Laakasuo et al. [37] and Grinschgl et al. [9], showing a positive correlation between Science-Fiction hobbyism and the acceptance of cognitive enhancement methods. This effect might be caused by the familiarity with cognitive enhancement methods, which are often portrayed in Science-Fiction media [9].

Research on the psychological predictors of cognitive enhancement acceptance is still emerging, often inconsistent, and mostly focused on single enhancement methods. The present study therefore examines a range of individual difference variables: those conceptually or empirically linked to cognitive enhancement acceptance in previous work, such as personality traits, intelligence, and science-fiction hobbyism, as well as less frequently examined but conceptually plausible predictors such as novelty seeking and trait anxiety. The goal is to establish a broader empirical foundation from which more focused theoretical models on the predictors of cognitive enhancement acceptance can be developed in the future.

### 1.5. Motives for the acceptance of cognitive enhancement

In addition to quantitatively investigating individual differences as predictors for the acceptance of cognitive enhancement methods, we also aimed to qualitatively assess individuals’ motives for their willingness to use (or not use) cognitive enhancement methods.

Previous research on motives for cognitive enhancement has predominantly focused on single methods—most commonly pharmaceutical enhancement—without considering a broader spectrum of methods. For example, studies among university students have consistently linked the use of pharmaceutical enhancement to workload and stress management, such as improving concentration, memory, or extending waking hours [58,59]. Similarly, a large-scale qualitative study by De Santis et al. [60] on the non-medical use of ADHD medication among university students found that these substances were primarily used to achieve better academic outcomes, including increased wakefulness, concentration, and memory. More recent, smaller-scale qualitative studies [61,62] have reported similar findings. Some authors even suggest that the prevailing narrative around ‘cognitive enhancement’ to improve cognition is flawed, as the actual motives are predominantly emotional, motivational, and stress-related (see [1] for a summary).

Relatively little research has investigated why individuals choose to not apply cognitive enhancement methods. For instance, a study on Brazilian university students [63] identified reluctance to use pharmaceutical enhancement due to concerns about side effects, limited access, and ethical considerations. Other studies have highlighted broader concerns regarding pharmaceutical enhancement, such as medical safety, fairness (i.e., getting an unfair advantage through enhancement or availability) and coercion [34].

In the present studies, we sought to provide an initial understanding why individuals would decide to apply enhancement methods or be reluctant to do so. Thus, we aimed to explore the motives for the acceptance and rejection of a broad range of cognitive enhancement methods, offering a detailed and nuanced perspective and providing exploratory insights into the underlying motives for both active and passive cognitive enhancement methods.

### 1.6. Present studies

In the present studies, we investigated a range of individual differences that might be related to the acceptance of active and passive cognitive enhancement methods. Furthermore, we conducted qualitative analyses to assess the motives for the acceptance of cognitive enhancement methods. Our research questions and hypotheses were preregistered and are outlined in Table 1.

**Table 1:** Preregistered research questions and hypotheses in Study 1 and 2.

Study 1	Study 2
*RQ1:* What is the underlying factor structure of the of the Trans- and Posthumanistic Enhancement Questionnaire?	*H1:* The factor structure of the Trans- and Posthumanistic Enhancement Questionnaire with the factors active and passive enhancement can be confirmed.
*RQ2:* Are there significant correlations between psychometric intelligence and the AoE?	*RQ1:* Are there significant correlations between psychometric intelligence and the AoE?
*RQ3:* Are there significant correlations between self-estimated intelligence and the AoE?	*RQ2:* Are there significant correlations between self-estimated intelligence and the AoE?
*RQ4:* Are there significant correlations between the Big Five factors (agreeableness, conscientiousness, extraversion, openness, neuroticism) and the AoE?	*H2:* There is a significant negative correlation between conscientiousness and the acceptance of passive enhancement methods.
–	*RQ3:* Is there a significant correlation between conscientiousness and the acceptance of active enhancement methods?
–	*RQ4:* Are there significant correlations between the six facets of conscientiousness (competence, order, dutifulness, achievement striving, self-discipline, and deliberation) and the AoE?
*H1:* There are significant positive correlations between the Dark Triad traits machiavellism and grandiose narcissism and the AoE.	–
*RQ5:* Are there significant correlations between between the Dark Triad trait psychopathy and the AoE?	–
–	*RQ5:* Are there significant correlations between trait-anxiety and the AoE?
–	*RQ6*: Are there significant correlations between novelty-seeking and the AoE?
*RQ6:* Are there significant correlations between a person’s self-esteem and the AoE?	–
*RQ7:* Are there significant correlations between the different dimensions of interests (RIASEC) and the AoE?	–
*H2:* There are significant positive correlations between Science-Fiction hobbyism and the AoE.	*H3:* There are significant positive correlations between Science-Fiction hobbyism and the AoE.
*RQ8:* Do psychometric and self-estimated intelligence predict the AoE in addition to control variables (e.g., gender, age, education), personality traits, self-esteem, interests, and Science-Fiction hobbyism?	*RQ7:* Do novelty-seeking, trait anxiety, conscientiousness, and intelligence (psychometric and self-estimated) predict the AoE in addition to control variables and Science-Fiction hobbyism?
	*RQ8:* What are the motives behind the acceptance of active and passive enhancement methods?
	*RQ9:* What are the motives behind the rejection of active and passive enhancement methods?

*Note.* AoE = Acceptance of cognitive enhancement methods. All research questions/hypotheses were tested for the acceptance of active and passive enhancement methods. Hypotheses are confirmatory; research questions are planned exploratory due to inconclusive or limited prior evidence. RQ8 (Study 1) and RQ7 (Study 2) were preregistered to only include significantly correlating factors in the hierarchical regression models. Please also note that those research questions were slightly differently phrased in the preregistration. As this original phrasing was not fully correct for our preregistered hierarchical regression models, we adapted the phrasing slightly so that it fully represents the corresponding analyses.

First, we aimed to assess the acceptance of these cognitive enhancement methods by using the Trans- and Posthumanistic Enhancement Questionnaire [9], Thus, we tested the factor structure of this questionnaire aiming to replicate the factor-analytical assignment of the cognitive enhancement methods to passive and active enhancement as observed by Grinschgl et al. [9]. While in Study 1, this was done by a planned exploratory factor analysis, in Study 2 we investigated the previously observed factor structure with a confirmatory factor analysis.

Furthermore, we investigated the correlations between psychometrically measured intelligence as well as self-estimated intelligence with the acceptance of cognitive enhancement methods in both studies. As previous results on those relationships are inconclusive [9], we did not have specific hypotheses with this regard.

In Study 1, we tested the relationships of the Big 5 and Dark triad traits and the acceptance of cognitive enhancement methods. Relationships with the Big 5 traits and the Dark triad trait psychopathy were tested exploratorily, while we expected significant positive correlations between the acceptance of cognitive enhancement methods and the Dark Triad traits Machiavellism and grandiose narcissism [9]. In Study 2, we investigated conscientiousness and its sub-facets in relation to the acceptance of cognitive enhancement methods. We expected a significant negative correlation between conscientiousness and the acceptance of passive cognitive enhancement methods [9]. The relationships of conscientiousness and the acceptance of active cognitive enhancement methods, as well as relationships with the subfacets (competence, order, dutifulness, achievement striving, self-discipline, and deliberation) were analyzed exploratorily. Furthermore, in Study 2 we also exploratorily investigate the relationships of trait anxiety as well as novelty seeking with the acceptance of cognitive enhancement methods, while in Study 1 we tested the relationship between self-esteem and the acceptance of cognitive enhancement methods.

We also tested how interests might be related to the acceptance of cognitive enhancement methods. In Study 1, we assessed participants’ RIASEC interests and exploratorily investigate their relationship with acceptance. In Study 1 and 2, we assessed Science-Fiction hobbyism and expected a significant positive correlation with the acceptance of cognitive enhancement methods [9].

Finally, we aimed to assess whether the tested factors explain variance in the acceptance of active and passive cognitive enhancement methods in hierarchical multiple regression models in both studies. Additionally, we qualitatively analyzed motives for the acceptance as well as rejection of active and passive cognitive enhancement methods in Study 2.

## 2. Method study 1

This study was conducted in the laboratory of the Institute of Psychology, University of Graz, where data collection took place between 03 February 2023 and 17 March 2023. The study was preregistered on the Open Science Framework (OSF): https://osf.io/tj5vb, and the data and analysis code are available here: https://osf.io/qbfsc.

### 2.1. Participants

To determine the sample size for this study, we conducted a power analysis using G*Power (version 3.1 [64]) for bivariate correlations and two-sided testing). To observe an effect size of *r* = .20 with a statistical power of .80 and an alpha level of .05, data of 193 participants need to be collected. As preregistered, we rounded our sample size up to 200 participants.

For study participation, participants had to be between 18 and 65 years old and speak German at least at C1-level. Due to a miscommunication with our research assistant who was responsible for data collection, we slightly oversampled and collected data of 206 participants. We excluded one participant because the answers to the paper-pencil intelligence tests were not readable, and two participants due to an experimenter error which did not allow us to allocate which paper-pencil test belongs to which of the two participants. Thus, we analyzed a final sample of 203 participants. Our sample had a medium age of 29.00 years (*SD* = 10.29), and included 121 women and 82 men. As their highest educational qualification, 4 participants stated a compulsory education, 15 participants an apprenticeship, 96 participants a high school diploma or an equivalent university entrance qualification, 48 participants a bachelor’s degree, 38 participants a master’s degree, one participant a PhD, and one participant indicated “other”.

Participants were recruited via social media, university mailing lists, and flyers. For 90 minutes of study participation, the participants received 15€ or course credits (the latter was only available to psychology students). All participants provided their written informed consent, and the study was approved by the ethics committee of University of Graz. All measures were used in their German version and in the order as presented below. With exception of the paper-pencil intelligence tests, all measures were implemented via LimeSurvey and presented digitally on lab computers. Descriptive statistics and Cronbach’s α are depicted in Table 2.

**Table 2:** Descriptive Statistics of the Main Variables in Study 1.

Variable	*M*	*SD*	Cronbach’s α
**Acceptance of Enhancement**			
Passive Enhancement Methods	2.85	1.01	.70
Pharmacological Enhancement	3.11	1.47	–
Current-based Enhancement	3.37	1.43	–
Genetic Enhancement	2.62	1.37	–
Brain-Machine Interface	2.31	1.33	–
Active Enhancement Methods	4.41	1.04	.70
Working Memory Training	4.80	1.17	–
Game-based Enhancement	4.20	1.41	–
Neurofeedback Training	4.23	1.36	–
**Measured General Intelligence** (z-score)	0.01	0.78	–
**Verbal Intelligence** (sum score)	10.87	3.82	.76
**Numerical Intelligence** (sum score)	8.86	4.02	.80
**Spatial Intelligence** (sum score)	11.17	4.97	.92
**Self-Estimated General Intelligence** (IQ)	107.24	9.76	–
**Big Five**			
Extraversion	3.46	0.88	.85
Agreeableness	3.21	0.77	.66
Conscientiousness	3.62	0.69	.71
Neuroticism	3.18	0.81	.75
Openness	4.05	0.62	.64
**Dark Triad**			
Machiavellianism	2.79	1.21	.80
Psychopathy	2.57	1.01	.55
Grandiose Narcissism	3.56	1.19	.79
**Self-Esteem**	4.58	0.78	.89
**General Interests**			
Realistic	2.58	0.80	.86
Investigative	3.18	0.76	.84
Artistic	3.18	0.84	.86
Social	3.41	0.75	.86
Enterprising	2.87	0.77	.85
Conventional	2.55	0.65	.77
**Science-Fiction Hobbyism**	3.19	1.17	.88

*Note. N* = 203.

**Self-Esteem.** To measure participants’ self-esteem, we used the German translation of the Rosenberg-Self-Esteem-Scale [65] by Von Collani and Herzberg [66]. This scale includes 10 items that need to be answered on a 6-point Likert scale from “1 - not at all” to “6 -completely”. A high mean value across the 10 items indicated high self-esteem.

**Acceptance of cognitive enhancement methods.** Participants acceptance of cognitive enhancement methods was measured with a previously developed and tested questionnaire [9,39]. This questionnaire included descriptions (i.e., short vignettes) of 8 different cognitive enhancement methods (see https://osf.io/du39z/). For the present study, we choose 7 out of these 8 methods: pharmacological enhancement, brain stimulation, genetic enhancement, working memory training, game-based enhancement, neurofeedback-training, and brain-computer interfaces. We excluded the vignette for mind upload, as it does not describe the enhancement of one’s cognitive functioning during one’s lifetime, but rather the preservation or continuation of the mind without a physical body (i.e., beyond physical death).

For each cognitive enhancement method, the participants answered three questions on a 6-point Likert scale from “1 – strongly disagree” to “6 – strongly agree”. The first question—which is also our main variable indicating participants’ acceptance of cognitive enhancement—asked whether participants would use the respective methods to enhance their cognitive abilities. The second question targeted the acceptance of enhancement for other people, and the third question if participants have concerns about applying the respective enhancement method accompanied by an open question to write down the biggest concerns. These latter two questions were only included for exploratory purposes as described in the preregistration. A thematic analysis of the open-ended responses can be found in the supporting information (S1 and S2 Tables).

To keep participants’ attention, for half of the participants three cognitive enhancement methods (pharmacological enhancement, brain stimulation, and genetic enhancement) and the corresponding questions were presented before the following self-estimates and intelligence tests, the other four methods and corresponding questions were presented after the intelligence test. For the other half of participants, this order was reversed. Within each presentation block, the order of presented enhancement methods was randomized. We tested the underlying factor structure of this questionnaire and, depending on the derived factors, calculated mean scores across the respective enhancement methods for each factor (see results section).

**Self-estimated Intelligence.** To assess participants’ self-estimated intelligence, we used two different measures. With a single-item scale, the participants estimated their IQ in comparison to the population by moving a slider on a bell curve, ranging from an IQ of 55 (“strongly below-average”) to 145 (“strongly above-average) [67,68] (see https://osf.io/76drs/). The participants answered this scale for verbal, numerical, spatial, and general intelligence. With a multi-item scale, participants rated their verbal, numerical, and spatial intelligence with 9-10 questions each [69]. The participants rated questions like “I’m very good at logical thinking” on a 5-point Likert scale from “1 – strongly disagree” to “5 – strongly agree”. A mean score for each intelligence dimension can be calculated. Please note that the latter measure (multi-item self-estimates) were collected for exploratory purposes only, as outlined in the preregistration. Descriptive statistics and a correlation table between those self-estimates and the acceptance of passive and active enhancement can be found in the supporting information (S3 and S4 Tables).

**Intelligence.** Following participants’ self-estimates of their intelligence, they answered the Wilde intelligence test (WIT-2) [70]. For our study, we selected one test for each intelligence domain based on the reported reliabilities and necessary time to perform the test. For verbal intelligence, the participants performed the analogies test including 20 items and taking 4:30 minutes. For numerical intelligence, they performed the test “arithmetic problems” (in German “eingekleidete Rechenaufgaben”) including 20 items and taking 16 minutes. For spatial intelligence, participants performed the test “mirrored images” also including 20 items and taking 3 minutes. Please note that we reported wrong test durations in our preregistration. Here, the correct times and as they were administered in the study are described. As an indicator for participants’ general intelligence, we averaged the z-transformed sum scores of the three domains. Exploratory analyses with the intelligence subscales can be found in S4 Table in the supporting information.

**Big 5 Personality.** To assess the Big Five personality factors, we used the German short version of the Big-Five-Inventory (BFI-K) [71]. The questionnaire contains 21 items which are rated on a 5-point Likert scale ranging from “1 - very inapplicable” to “5 - very applicable”. For each factor (extraversion: 4 items; agreeableness: 4 items; conscientiousness: 4 items; neuroticism: 4 items; openness: 5 items), we calculated mean values using the corresponding items.

**Dark Triad.** In addition to the Big 5 personality traits, we also assessed participants’ dark personality traits with the Dirty Dozen Questionnaire [72] in its German version by Küfner et al. [73]. With a total of 12 items, the questionnaire measured participants’ Machiavellianism, grandiose narcissism, and psychopathy levels (4 items each). Participants rated statements on a 9-point Likert scale ranging from “1 - not true at all” to “9 - exactly true”. We calculated mean values for each of the three dark triad traits using the corresponding items. The internal consistency was rather low for psychopathy (see Table 2), but acceptable for Machiavellianism and grandiose narcissism.

**General Interests.** As a measure for participants’ interest, we used the “Allgemeiner Interessensstruktur-Test-Revised” (AIST-R) [74] measuring interests based on the RIASEC model [57]. With 60 items, we assessed Realistic, Investigative, Artistic, Social, Enterprising, and Conventional interests (10 items each). The participants had to rate each item on a 5-point Likert scale from “1 – I’m very interested in this, I like doing this very much” to “5 – I’m not interested in this at all, I don’t like doing this”. We computed mean values for each dimension using the corresponding items.

**Science-Fiction hobbyism.** To measure participants’ interest in Science-Fiction, we used the German translation of the Science-Fiction-Hobbyism Scale [9,37]. The scale contains 11 items that need to be rated on a 7-point Likert scale ranging from “1 – strongly disagree” to “7 – strongly agree”, for which a mean value is computed.

## 3. Results study 1

For Study 1, we first tested the underlying factor structure of our questionnaire measuring the acceptance of cognitive enhancement methods (RQ1, see Table 1). Based on the results of this factor analysis, we tested the correlations between potential predictors (e.g., intelligence, personality) and the acceptance of cognitive enhancement methods, either as confirmatory tests with specific hypotheses (H1-2) or preregistered and planned exploratory analysis (RQ2–7), in cases where prior evidence was limited or non-conclusive. Besides calculating Pearson correlations, we also calculated preregistered Bayes Factors for these analyses. These Bayes Factors serve as complementary evidence in favor of the null over the alternative hypothesis and were computed using a default Jeffreys-type Bayesian test for linear correlation [75], with noninformative priors on the population means and variances and a symmetric, and a weakly informative prior on the correlation coefficient under the alternative hypothesis.

We additionally report follow-up exploratory (i.e., not preregistered) Bonferroni–Holm–corrected *p*-values for bivariate correlations within each test family (i.e., for the Big Five, Dark Triad, and general interests; see S5 Table). The rationale for this selective correction is that multiplicity concerns are most relevant when multiple correlated outcomes are drawn from the same measurement instrument.

An intercorrelation table of all variables can be found in the supporting information (S6 Table). Finally, we also tested the explained variance in the acceptance of cognitive enhancement methods due to the observed predictors in hierarchical regression models (RQ8) as preregistered. We calculated bootstrapped confidence intervals based on 2000 samples for all correlational and regression analyses. In our preregistration, we planned to calculate these confidence intervals only if the requirements of normality and/or homoscedasticity are violated, however, in the end we decided to calculate it for all correlations and regressions to get robust estimates. All analyses were two-tailed.

### 3.1. RQ1: Acceptance of cognitive enhancement methods

As observed in a previous study [9], the acceptance of cognitive enhancement methods can—theoretically and empirically—be separated in active and passive methods. Following up on this previous study, we tested the underlying factors structure of the enhancement questionnaire with a preregistered (i.e., planned) exploratory factor analysis, using the Maximum-Likelihood method with Varimax rotation. The Kaiser-Meyer-Olkin value, KMO = .80 as well as Bartlett’s test of sphericity, χ2(21) = 308.49, *p* < .001 suggest that our data are adequate for this analysis. Both the Scree plot and Eigenvalues suggest the extraction of two factors that together explain a variance of 42.49%. Replicating our previous study [9], 4 out of the 7 tested cognitive enhancement methods (pharmacological enhancement, brain stimulation, genetic enhancement, brain computer interfaces) form the first factor, Eigenvalue = 2.91, explained variance 33.73%, while the remaining 3 methods (working memory training, game-based enhancement, neurofeedback training) form the second factor, Eigenvalue = 1.16, explained variance 8.75% (see Table 3). Based on these results, we conducted further analyses on these two factors—the first factor representing passive cognitive enhancement methods such as pharmacological enhancement which require relatively little active participation of the user, and the second factor representing active cognitive enhancement methods such as working memory training which require a high effort and commitment of the user. We calculated the mean values across the acceptance item for the respective cognitive enhancement methods for each factor.

**Table 3:** Rotated factor matrix.

	Factor 1 (Passive Enhancement)	Factor 2 (Active Enhancement)
Pharmacological Enhancement	**.59**	.26
Current-based Enhancement	**.50**	.40
Genetic Enhancement	**.75**	.06
Brain Machine Interface	**.44**	.19
Working Memory Training	.09	**.62**
Game-based Enhancement	.21	**.59**
Neurofeedback Training	.31	**.68**

*Note. N* = 203*.* Extraction method: Maximum Likelihood. Rotation method: Varimax. Factor loadings above  .40 are depicted in bold.

For additional exploratory purposes and to replicate our previous study [9], we calculated a non-preregistered t-Test comparing the acceptance of these two forms of cognitive enhancement. Same as for our previous study, we observed a higher acceptance for active than passive cognitive enhancement methods, *t*(202) = −20.88, *p* < .001, *d* = −1.52. As preregistered, we also calculated an ANOVA to compare the acceptance of all 7 cognitive enhancement methods. The Greenhouse-Geisser corrected results for missing sphericity, suggest a large difference between the acceptance of cognitive enhancement methods, *F*(5.44, 1099.86) = 134.59, *p* < .001, *η*^*2*^ = .28. Bonferroni-corrected post-hoc tests indicate significant differences in the acceptance of most cognitive enhancement methods, all *p*s < .001 (see also Table 2 for the means of each method and S7 Table for the post-hoc comparisons). Only between the acceptance of pharmacological and current-based enhancement, genetic enhancement and brain-machine interfaces, as well as game-based enhancement and neurofeedback training we did not observe significant differences, all *p*s >= .097.

### 3.2. RQ2–3: Psychometrically measured and self-estimated intelligence and acceptance of enhancement

Investigating RQ2 (planned exploratory analyses), we observed no significant relationship between psychometrically measured intelligence and acceptance of passive cognitive enhancement methods (see Table 4). For active cognitive enhancement methods, we observed a significant positive relationship, indicating a higher acceptance of active cognitive enhancement methods being related to a higher intelligence. For self-estimated intelligence (RQ3; planned exploratory analyses), no significant relationships—neither with passive nor active cognitive enhancement were observed.

**Table 4:** Correlational Analyses of Main Variables in Study 1.

	Passive Enhancement		Active Enhancement	
	*r* [95% CI]	*BF*_01_	*r* [95% CI]	*BF*_01_
**Control Variables**				
Age	−.10 [−.24; .03]	2.10	−.29*** [−.41; −.16]	<0.01
Education	.08 [−.06; .20]	–	.05 [−.09; .18]	–
Gender	.19** [.05; .32]	0.20	.09 [−.05; .22]	2.91
**Measured General Intelligence (z-score)**	.05 [−.09; .18]	4.97	.19** [.06; .32]	0.15
**Self-Estimated General Intelligence (IQ)**	.11 [−.02; .25]	1.72	.11 [−.02; .25]	1.68
**Big Five**				
Extraversion	−.01 [−.15; .13]	6.04	−.03 [−.17; .11]	5.55
Agreeableness	−.09 [−.23; .05]	2.66	−.01 [−.15; .12]	6.00
Conscientiousness	−.17* [−.30; −.04]	0.31	−.16* [−.29; .02]	0.47
Neuroticism	.11 [−.03; .24]	1.83	.12 [−.02; .25]	1.58
Openness	.02 [−.12; .16]	5.87	.05 [−.09; .19]	4.65
**Dark Triad**				
Machiavellianism	.20** [.07; .33]	0.09	.13 [−.01; .26]	1.25
Psychopathy	.09 [−.05; .23]	2.69	< .01 [-.13; .15]	6.07
Grandiose Narcissism	.18* [.04; .31]	0.29	.19** [.05; .32]	0.20
**Self-Esteem**	−.06 [−.20; .08]	4.31	−.06 [−.20; .07]	4.09
**General Interests**				
Realistic	.03 [−.11; .16]	5.76	.02 [−.12; .15]	5.99
Investigative	.18** [.05; .31]	0.22	.26*** [.13; .39]	< 0.01
Artistic	<-.01 [-.14; .14]	6.13	.07 [−.06; .21]	3.54
Social	−.12 [−.25; .02]	1.53	.05 [−.09; .18]	4.90
Enterprising	−.07 [−.21; .07]	3.66	−.08 [−.22; .06]	3.18
Conventional	−.12 [−.25; .02]	1.52	−.15* [−.28; −.01]	0.70
**Science-Fiction Hobbyism**	.31*** [.17; .43]	<0.01	.23*** [.10; .36]	0.30

*Note.* * *p* < .05. ** *p* < .01. *** *p* < .001. *N* = 203. *BF*_01_ indicates evidence for the null hypothesis over the alternative hypothesis. For gender, a value of 0 indicates females and 1 males. Education (*n* = 202) was measured with a 6-point ordinal scale, thus, we calculated Spearman correlations (and no Bayes factor) for this variable. Confidence intervals depict 95% BCa bootstrapping confidence intervals for 2000 samples.

### 3.3. RQ4–5 & H1: Personality and acceptance of cognitive enhancement

With regard to the Big 5 factors (RQ4, planned exploratory analyses), we observed no significant relationships for extraversion, agreeableness, neuroticism, and openness and rather high Bayes Factors favoring the null hypothesis (see Table 4). For conscientiousness, we observed significant negative correlations with both the acceptance of passive and active cognitive enhancement methods, but these did not remain significant after follow-up Bonferroni-Holm correction (see S5 Table). As expected (H1; confirmatory analyses), we observed significant positive correlations of Machiavellianism and grandiose narcissism with the acceptance of passive cognitive enhancement methods. For active cognitive enhancement methods, we only observed a significant positive correlation with grandiose narcissism (i.e., H1 is partially supported). No significant correlations were observed with psychopathy (RQ5, planned exploratory analyses).

### 3.4. RQ6: Self-esteem and acceptance of enhancement

As shown in Table 4, no significant correlations were found for participants’ self-esteem and the acceptance of passive and active cognitive enhancement methods (planned exploratory analyses).

### 3.5. RQ7 & H2: Interests and acceptance of enhancement

For the RIASEC interests (RQ7, planned exploratory analyses), we observed a significant positive relationship between investigative interests and both the acceptance of passive and active cognitive enhancement methods (see Table 4), but only the association with active cognitive enhancement remained significant after follow-up Bonferroni-Holm correction (see S5 Table). In addition, conventional interests showed a significant negative correlation with the acceptance of active cognitive enhancement methods (but not after Bonferroni-Holm correction; see S5 Table). All other correlations for the RIASEC interests were non-significant and showing rather high Bayes Factors favoring the null hypothesis. For Science-Fiction hobbyism, as expected we observed a positive correlation with both passive and active cognitive enhancement methods, confirming H2 (confirmatory analyses).

### 3.6. RQ8: Hierarchical regression

For our final preregistered research question (planned exploratory analyses), we tested whether psychometric intelligence explains variance in the acceptance of cognitive enhancement methods in addition to personality traits and interests. As preregistered first, we added significantly correlating control variables (e.g., age, gender; see Table 4) to our regression models (Step 1), followed by psychometric intelligence (Step 2) and self-estimated intelligence (Step 3) if they significantly correlated. The fourth step included significantly correlating personality traits and the fifth step significantly correlating interests. In line with our preregistered analysis plan, we selected predictors for the regression model based on uncorrected bivariate significance, in order to avoid an overly conservative approach that could inflate Type II error.

For passive cognitive enhancement methods, we first entered gender as a control variable, explaining 3% of variance (see Table 5). As psychometric and self-estimated intelligence did not significantly correlate with the acceptance of passive cognitive enhancement methods (see Table 2), they were not entered into the regression model. Instead, as step 2 we entered conscientiousness, Machiavellianism, and grandiose narcissism (model explaining 8% of variance). Finally, we added investigative interests and Science-Fiction hobbyism, resulting in an overall explained variance of 13%. Science-Fiction hobbyism is the strongest–and in the final model the only significant–predictor for the acceptance of passive cognitive enhancement methods.

**Table 5:** Multiple Hierarchical Regression Analyses with the Criteria “Acceptance of Passive Enhancement” and “Acceptance of Active Enhancement” (Study 1).

		*R* ^*2*^	*∆R* ^*2*^	*∆F*	*B*	*t*
**Passive Enhancement**						
Model 1		.03	.03	7.16**		
	Gender				.38 [.09; .68]	2.68**
Model 2		.08	.04	2.94*		
	Gender				.27 [−.03; .58]	1.82
	Conscientiousness				−.12 [−.33; .11]	−1.06
	Machiavellianism				.10 [−.03; .25]	1.50
	Grandiose Narcissism				.06 [−.08; .19]	0.89
Model 3		.13	.05	5.66**		
	Gender				.06 [−.28; .40]	0.40
	Conscientiousness				−.09 [−.29; .12]	−0.86
	Machiavellianism				.09 [−.03; .24]	1.47
	Grandiose Narcissism				.04 [−.11; .17]	0.59
	Investigative Interests				.02 [−.19; .26]	0.21
	Science-Fiction Hobbyism				.21 [.05; .37]	2.82**
**Active Enhancement**						
Model 1		.08	.08	18.43***		
	Age				−.03 [−.04; −.01]	−4.29***
Model 2		.10	.01	2.88		
	Age				−.03 [−.04; −.01]	−3.63***
	Measured General Intelligence				.16 [−.02; .36]	1.70
Model 3		.12	.03	2.96		
	Age				−.02 [−.04; −.01]	−3.41**
	Measured General Intelligence				.12 [−.05; .33]	1.24
	Conscientiousness				−.17 [−.37; .04]	−1.64
	Grandiose Narcissism				.09 [−.03; .20]	1.41
Model 4		.18	.05	4.11**		
	Age				−.02 [−.04; −.01]	−3.16**
	Measured General Intelligence				.02 [−.18; .23]	0.16
	Conscientiousness				−.11 [−.32; .10]	−1.02
	Grandiose Narcissism				.09 [−.03; .20]	1.51
	Conventional Interests				−.16 [−.37; .04]	−1.49
	Investigative Interests				.31 [.10; .52]	2.68**
	Science-Fiction hobbyism				.01 [−.13; .13]	0.08

*Note.* *p < .05, **p < .01, ***p < .001; *N* = 203; For gender, a value of 0 indicates females and 1 males. Confidence intervals for *B* depict 95% BCa bootstrapping confidence intervals for 2000 samples.

For active cognitive enhancement methods, we entered age as a control variable, explaining 8% of variance, followed by psychometric intelligence (model explaining 9% of variance). Next, we entered conscientiousness and narcissism, resulting in a model explaining 12% of variance. Finally, we entered conventional and investigative interests as well as Science-Fiction hobbyism with an overall explained variance of 17%. In the final model, age and investigative interests are the strongest and the only significant predictors for the acceptance of active cognitive enhancement methods.

## 4. Method study 2

Building upon previous research [9] and Study 1, we sought to further validate and expand these findings in Study 2. To confirm the robustness of the factor structure identified in the exploratory factor analysis of Study 1, we calculated a preregistered confirmatory factor analysis (CFA). Additionally, we aimed to (conceptually) replicate key findings from Study 1, including the relationships with science-fiction hobbyism, self-estimated and psychometrically measured intelligence. We also aimed to gain a deeper understanding about the relationship between the acceptance of cognitive enhancement methods and conscientiousness, by looking at the subfacets of conscientiousness. Furthermore, we explored new predictors, namely trait anxiety and novelty seeking, that may influence the acceptance of cognitive enhancement methods. Finally, we qualitatively investigated the underlying motives driving individuals to accept or reject enhancement, thereby providing a more comprehensive understanding of the factors influencing cognitive enhancement acceptance. This study was conducted in the laboratory of the Institute of Psychology, University of Graz, where data collection took place between 28 June 2023 and 24 November 2023, and was preregistered on the Open Science Framework: https://osf.io/t4qd9. The study data and analysis code are also available on OSF: [https://osf.io/zyxh9.

### 4.1. Participants

The sample size for this study was determined identical to Study 1. As preregistered, we rounded up the sample size to 200 participants. As a prerequisite for participation, participants had to be between 18 and 65 years old and possess sufficient German language skills, either as a native speaker or at a C2 level. A total of 200 people took part in the study. We excluded participants with incomplete datasets (*n* = 3), which results in a deviation from the preregistered sample size of 200 participants. The 197 remaining participants were between 18 and 63 years old (*M* = 26.96, *SD* = 9.85). 132 participants identified as female, 64 as male, and one as diverse. As their highest educational qualification, five participants stated a compulsory education, six participants an apprenticeship, 127 participants a high school diploma or a university entrance qualification, 40 participants a bachelor’s degree, 17 participants a master´s degree and two participants a PhD.

Participants were recruited via social media, university mailing lists, flyers, announcements in university lectures, and with the help of Probando, a participant recruitment company. As an incentive for participation, subjects received an reimbursement of 10€ or a course credit for psychology students. All participants provided their written informed consent, and the study was approved by the ethics committee of University of Graz.

### 4.2. Material

All measures were used in their German translation, implemented via LimeSurvey and presented digitally on lab computers in the order as outlined below. Descriptive statistics and Cronbach’s α are depicted in Table 6.

**Table 6:** Descriptive Statistics of all Variables Relevant for Quantitative Analyses of Study 2.

Variable	*M*	*SD*	Cronbach’s α
**Acceptance of Enhancement**			
Passive Enhancement	3.01	1.03	0.53
Brain-Machine-Interface	2.43	1.47	–
Current-based Enhancement	3.48	1.33	–
Pharmacological Enhancement	3.10	1.49	–
Active Enhancement	4.46	1.01	0.70
Neurofeedback-Training	4.20	1.35	–
Game-based Enhancement	4.47	1.34	–
Working Memory Training	4.73	1.15	–
**Intelligence**			
Psychometric Intelligence	28.44	5.28	0.81
Self-estimated Intelligence	105.74	8.25	–
**Personality Traits**			
Conscientiousness	121.96	22.05	0.91
Competence	22.24	4.03	0.68
Order	19.93	5.01	0.71
Dutifulness	23.13	4.26	0.67
Achievement Striving	19.48	4.53	0.64
Self-Discipline	18.63	6.18	0.86
Deliberation	18.55	5.05	0.74
Trait Anxiety	41.64	10.12	0.92
Novelty Seeking	19.49	5.71	0.76
**Science-fiction Hobbyism**	3.19	1.19	0.89

*Notes. N* = 197.

**Self-estimated intelligence.** To assess self-estimated intelligence, we utilized the single-item-scale by Hofer et al. [67,68] that was also used in Study 1.

**Intelligence.** Intelligence was measured with the Wonderlic Personnel Test by Wonderlic [76]. Participants had 12 minutes to answer up to 50 items. The test comprises questions of varying levels of difficulty, which are answered in different formats. The items cover various domains, including logical thinking and numerical, verbal, and spatial skills. A sum score was calculated and adjusted for the age of the participant, as outlined in the manual.

**Acceptance of enhancement methods and motives.** To assess the acceptance of cognitive enhancement methods and the motives behind their acceptance or rejection, the questionnaire used in Study 1 was adjusted. We assessed three active (working memory training, neurofeedback training, game-based enhancement) and three passive (pharmacological enhancement, current-based enhancement and invasive brain-machine-interfaces) cognitive enhancement methods from the questionnaire. We excluded the vignette for genetic enhancement, as we wanted to focus on methods that are already developed and accessible for a broader audience. This change likely reduced the internal consistency of the questionnaire relative to Study 1 (see Table 6).

The questionnaire was expanded to assess the motives for the acceptance and rejection of cognitive enhancement and previous experiences with the enhancement method. To assess the motives, participants had to state in an open response format, depending on their previous answer, why they would use (answers from “rather agree” to “strongly agree”) or would not use (answers from “strongly disagree " to “rather disagree“) the method. Afterwards, participants were asked if they had previously used this method to enhance their cognitive abilities. This latter question was only included for exploratory purposes. All cognitive enhancement methods were presented in a randomized order. Measures of acceptance of cognitive enhancement methods and motives were administered balanced before vs. after the following described measures of conscientiousness, anxiety, and novelty seeking, which were administered in a randomized order.

**Conscientiousness.** Conscientiousness was measured using the corresponding scale of the Revised NEO Personality Inventory (NEO-PI-R) [46] (German translation by [77]). The scale contains 48 items, with eight items in each of the six facets. Participants were asked to indicate the applicability of the statements to themselves using a five-point Likert scale ranging from “strongly disagree” to “strongly agree”. A sum score was computed for conscientiousness and each of the six facets.

**Trait Anxiety.** Trait anxiety was assessed with the 20-item version of the State-Trait Anxiety Inventory (STAI) [78] (German translation by [79]). Participants were asked to rate the general accuracy of the statements on a four-point response scale from “almost never” to “almost always.” A sum score was computed indicating trait anxiety.

**Novelty Seeking.** Novelty seeking was measured using the corresponding scale of the Temperament and Character Inventory (TCI) [80]. The scale comprises 40 items, each requiring a ‘yes’ or ‘no’ response to indicate the statement’s applicability. A sum score was calculated to indicate novelty seeking.

**Science-fiction hobbyism.** Science-fiction hobbyism was measured with the Science-fiction hobbyism Scale [9,37].

## 5. Results study 2 – Quantitative

For Study 2, we first conducted a confirmatory factor analysis to confirm the factor structure of the Trans- and Posthumanistic Enhancement Questionnaire (see H1, Table 1) as observed in Study 1 and previous analyses [9]. To test correlations between potential predictors (e.g., intelligence, personality) and the acceptance of active and passive cognitive enhancement methods, we calculated Pearson correlations and Bayes Factors. As in Study 1, we analyzed these correlations with potential predictors either as confirmatory tests with specific hypotheses (H2-3) or as preregistered and planned exploratory analyses (RQ1–6) when prior evidence was limited or non-conclusive. We again report Bayes factors as complementary evidence for the null over the alternative hypothesis. As preregistered, we additionally examined scatter plots for all correlations, which did not indicate non-linearity. We additionally report follow-up (non-preregistered) Bonferroni–Holm–corrected *p*-values for bivariate correlations within each test family (i.e., Conscientiousness facets) in supplementary S8 Table. An intercorrelation table of all variables can be found in the supporting information (S9 Table). We also calculated planned exploratory hierarchical regression models to test the explained variance in the acceptance of active and passive cognitive enhancement methods (RQ7).

### 5.1. H1: Acceptance of cognitive enhancement methods

Due to the violated assumptions of the (bivariate and univariate) normal distribution and the existence of outliers, the maximum likelihood estimation method with robust standard errors (MLR) was applied to enable a model with robust estimation [81]. The latent variable was scaled by fixing the factor variance to 1. This method is frequently used to assess the dimensionality of a test [82].

We calculated χ^2^ to test the goodness of fit. The test showed significant results, χ^2^(8) = 16.21, *p* = .039. However, χ^2^ is less meaningful for non-normally distributed data due to overestimation of the standard error [81,83,84]. Therefore, we additionally assessed descriptive goodness-of-fit indices to examine the model fit. The descriptive goodness-of-fit indices (RMSEA, CFI, and SRMR; see Table 7) indicate an acceptable to good model fit.

**Table 7:** Descriptive Goodness-of-Fit Indices.

χ^2^/df	RMSEA [90% CI]	CFI	SRMR
2.03	0.080 [0.017; 0.137]	0.952	0.045

*Notes. N* = 197.

**Fig 1:**
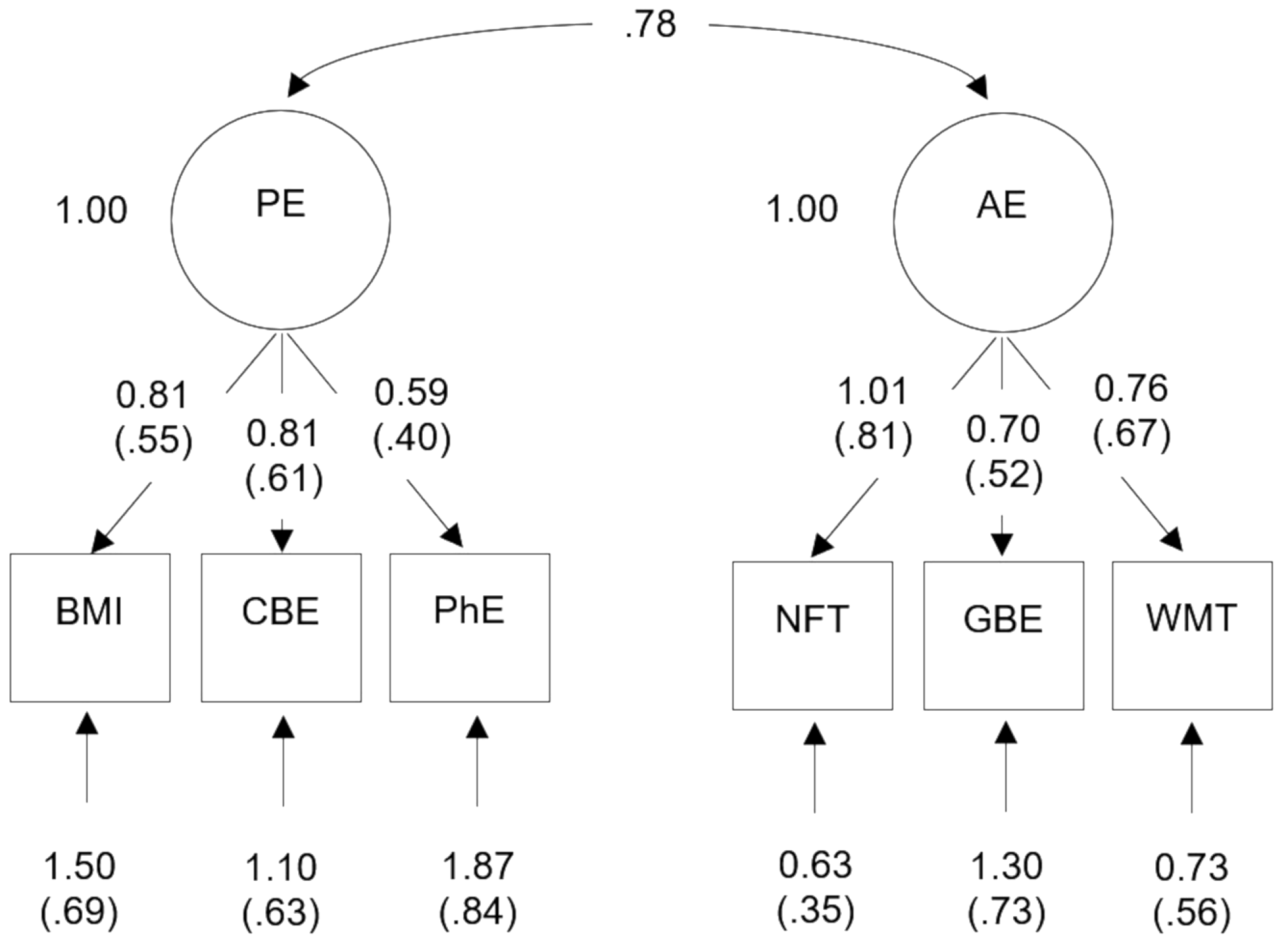
Completely standardized parameter estimates of the confirmatory factor analysis model. *Notes.* PE = Passive Enhancement Methods, AE = Active Enhancement Methods*,* BMI = Brain-Machine-Interface, CBE = Current-based Enhancement, PhE = Pharmacological Enhancement*,* NFT = Neurofeedback-Training, GBE = Game-based enhancement, WMT = Working-Memory Training.

The standardized parameter estimates can be found in Fig 1. We used the Critical Ratio test to test the significance of the model parameters. This test should be significant for all parameters in an appropriate model. All parameters show a significant value of z > ± 1.96 (Critical Ratio Test), which illustrates that the indicators load significantly (all *p* < .001) on the latent factor. This further verifies the goodness of model fit. The results of the confirmatory factor analysis support the hypothesized factor structure of active and passive cognitive enhancement methods, as identified by Grinschgl et al. [9] and in Study 1. These findings further strengthen the validity of the proposed measurement model.

To further replicate previous findings, we compared the acceptance of active and passive cognitive enhancement methods with a follow-up exploratory, non-preregistered *t*-test. As expected, we observed a higher acceptance for active than passive cognitive enhancement methods, *t*(196) = 19.46, *p* < .001, *d* = 1.39.

### 5.2. RQ1–2: Psychometrically measured and self-estimated intelligence and acceptance of cognitive enhancement

We observed a significant positive correlation between psychometric intelligence and the acceptance of active cognitive enhancement methods, but not for passive methods (RQ1, planned exploratory analyses). For self-estimated intelligence (RQ2, planned exploratory analyses), we observed a significant positive relationship with the acceptance of active and passive cognitive enhancement methods (see Table 8).

**Table 8:** Correlational Analyses of Main Variables in Study 2.

	Passive Enhancement		Active Enhancement	
	*r* [95% CI]	*BF*_01_	*r* [95% CI]	*BF*_01_
**Control Variables**				
Age	−.03 [−.16; .09]	5.21	−.17* [−.35; −.01]	0.39
Education	.02 [−.12; .18]	–	.10 [−.05; .26]	–
Gender	.27*** [.13; .40]	<0.01	.20** [.06; .33]	0.16
**Measured General Intelligence**	.05 [−.10; .18]	4.77	.29*** [.16; .40]	<0.01
**Self-Estimated General Intelligence**	.22** [.09; .37]	0.04	.20** [.07; .34]	0.11
**Conscientiousness**	−.08 [−.19; .04]	3.25	.04 [−.10; .19]	5.05
Competence	−.03 [−.18; .11]	5.53	.06 [−.09; .19]	4.16
Order	−.13 [−.25; .01]	1.34	.05 [−.07; .19]	4.72
Dutifulness	−.15* [−.27; −.03]	0.68	.01 [−.15; .14]	5.97
Achievement Striving	.06 [−.08; .19]	4.53	.10 [−.04; .24]	2.39
Self-Discipline	−.08 [−.21; .05]	3.47	−.08 [−.22; .06]	3.25
Deliberation	−.03 [−.16; .10]	5.47	.09 [−.05; .23]	2.80
**Trait Anxiety**	.04 [−.07; .17]	5.32	.06 [−.08; .21]	4.37
**Novelty Seeking**	.08 [−.05; .20]	3.43	.09 [−.05; .21]	3.02
**Science-fiction Hobbyism**	.30*** [.17; .43]	<0.01	.32*** [.19; .43]	<0.01

Note. * p < .05. ** p < .01. * p < .001. *N* = 197. BF01 indicates evidence for the null hypothesis over the alternative hypothesis. For gender (*N* = 196), a value of 0 indicates females and 1 males. Education was measured with a 6-point ordinal scale, thus, we calculated Spearman correlations (and no Bayes factor) for this variable. Confidence intervals depict 95% BCa bootstrapping confidence intervals for 2000 samples.

### 5.3. H2 & RQ 3–4: Conscientiousness and acceptance of enhancement

Regarding the Big 5 factor conscientiousness, no significant relationships—neither with passive (H2) nor active cognitive enhancement methods (RQ3, planned exploratory analysis) were observed (see Table 8). Investigating the relationship with subfacets of conscientiousness (RQ4, planned exploratory analyses), we observed a significant negative relationship between dutifulness and the acceptance of passive cognitive enhancement methods, which did not remain significant after follow-up Bonferroni-Holm correction (see S8 Table). No other significant relationships between the six subfacets of conscientiousness and the acceptance of active or passive cognitive enhancement methods were found and rather high Bayes Factors favor the null hypothesis.

### 5.4. RQ 5–6: Trait anxiety, Novelty Seeking and acceptance of enhancement

As shown in Table 8, we did not observe a significant relationship (and rather high Bayes Factors favoring the null hypothesis) between the acceptance of active or passive cognitive enhancement methods and trait anxiety (RQ5) or novelty seeking (RQ6)—both planned exploratory analyses.

### 5.5. H3: Science-fiction hobbyism and acceptance of enhancement

As expected, a significant positive correlation between Science-fiction hobbyism and the acceptance of active and passive cognitive enhancement methods was observed (H3; confirmatory analyses; see Table 8).

### 5.6. RQ7: Hierarchical regression

We conducted two preregistered, planned exploratory hierarchical regression analyses to test whether intelligence and personality traits explain variance in the acceptance of cognitive enhancement methods in addition to relevant control variables and Science-fiction hobbyism, which had been identified as the most consistent predictor in previous studies. As preregistered, we entered significantly correlating control variables (e.g., age, gender) to our regression models (Step 1), followed by Science-fiction interest (Step 2). In the third step, we added intelligence, self-estimated intelligence, and personality traits if they significantly correlated (see Table 9). As in Study 1 and in line with our preregistered analysis plan, we selected predictors for the regression model based on uncorrected bivariate significance.

**Table 9:** Multiple Hierarchical Regression Analyses with the Criteria “Acceptance of Passive Enhancement” and “Acceptance of Active Enhancement” (Study 2).

		*R* ^*2*^	*∆R* ^*2*^	*∆F*	*B*	*t*
**Passive Enhancement**						
Model 1		.07		15.38***		
	Gender				.59 [.27; 92]	3.92***
Model 2		.12	.05	11.16**		
	Gender				.42 [.11; .73]	2.69**
	Science-Fiction Hobbyism				.21 [.09; .38]	3.34**
Model 3		.14	.02	2.19		
	Gender				.38 [.06; .70]	2.42*
	Science-Fiction Hobbyism				.17 [−.04; .30]	2.61**
	Self-estimated Intelligence				.01 [−.01; .03]	1.44
	Dutifulness				−.03 [−.05; .00]	-1.54
**Active Enhancement**						
Model 1		.07		7.40***		
	Age				−.02 [−.04; −.00]	−2.99**
	Gender				.45 [.11; .74]	2.62**
Model 2		.14	.07	15.24***		
	Age				−.02 [−.04; −.00]	−2.49*
	Gender				.25 [−.07; .54]	1.61
	Science-Fiction Hobbyism				.24 [.14; .35]	3.90***
Model 3		.19	.05	6.16**		
	Age				−.02 [−.03; −.00]	−2.27*
	Gender				.19 [−.09; .49]	1.23
	Science-Fiction Hobbyism				.21 [.11; .32]	3.40**
	Psychometric Intelligence				.04 [.02; .07]	3.25**
	Self-estimated Intelligence				< .01 [-.01; .02]	0.26

*Notes. N* = 197*.* *p < .05, ** *p* < .01, *** *p* < .001. For gender, 0 indicates female, 1 indicates male. We excluded one participant who reported a non-binary gender identity from these analyses. Confidence intervals for *B* depict 95% BCa bootstrapping confidence intervals for 2000 samples.

For the acceptance of passive cognitive enhancement methods, we added gender as a control variable, accounting for 7% of variance. The addition of Science-fiction hobbyism explained another 5% of variance (model explaining 12% of variance). Finally, we added self-estimated intelligence and dutifulness. The predictors collectively accounted for 14% of the variance in the acceptance of passive cognitive enhancement methods.

For the acceptance of active cognitive enhancement methods, we entered age and gender as control variables, explaining 7% of variance. Next, we entered Science-fiction hobbyism, resulting in a model explaining 14% of variance. Finally, we entered self-estimated and psychometric intelligence, with an overall explained variance of 19%.

In contrast to Study 1, the order of predictor entry was reversed in this preregistered hierarchical regression analyses. Our aim was to determine whether our predictors could explain additional variance beyond science fiction hobbyism, the best predictor identified in previous research [9] and Study 1. In additional follow-up (non-preregistered) exploratory regressions, we reversed the order of entry for Steps 2 and 3 so that it is similar to Study 1. Results indicated that the same set of predictors explained significant and unique variance in the outcome variable, irrespective of entry order.

## 6. Results study 2 – Qualitative

In addition to our quantitative analyses, we conducted four preregistered thematic qualitative text analyses to investigate the motives for the acceptance and rejection of active and passive cognitive enhancement methods. We aimed at a nuanced understanding of the diverse perspectives and motives accounting for individuals’ attitudes toward enhancement in Study 2. Furthermore, we exploratorily coded the open-ended responses from Study 1 using the coding schemes developed for the rejection of active and passive cognitive enhancement methods. Since participants were asked to state their concerns regarding each cognitive enhancement method—regardless of their acceptance or rejection of the method—and not their motivations for their acceptance or rejection (as in Study 2), the results are not directly comparable. Nevertheless, the findings can be found in the supporting information (S1 and S2 Tables).

For Study 2, the primary coding was conducted by one of the authors (LK). Data were analyzed using thematic qualitative text analysis after Kuckartz [85,86]. This method deviates from our preregistration as we preregistered to employ a qualitative content analysis after Mayring [87]. However, Kuckartz’s theoretical considerations are better suited to assessing our research question, as they more closely reflect inductive category development [85,88]. The method of Kuckartz [86] includes seven iterative phases:

Phase 1: Initial Text Work. In this initial step of the analysis, the text was thoroughly analysed in relation to the research question.
Phase 2: Developing Main Thematic Categories. In this phase, we set the coding system’s level of abstraction–i.e., how closely they adhere to the participants’ formulation–and the size of the coding unit was set to contain complete thoughts or sentences. Based on that, categories were constructed inductive (= data-driven) based on about 25% of the answers, resulting in a coding system of the main categories with description.
Phase 3: First Coding Process. During this initial coding process, the entire text was assigned to the main categories. As the categories in thematic coding are not mutually exclusive, a text passage could be coded with more than one category. Text passages that did not contain information related to the research question, and unclear text passages—e.g., referring to a previous answer that was not identifiable because each participant was presented with the different enhancement methods in a randomized order—remained uncoded.
Phase 4: Selecting Main Categories that require further distinction. The relevant categories were selected and text passages compiled.
Phase 5: Developing Sub-Categories. We inductively created sub-categories and-if necessary-second level sub-categories.
Phase 6: Second coding process. During this second coding process, text passages were assigned to the sub-categories.
Phase 7: Analysis and Presentation of Result. The results were analyzed, focusing on category-based analysis.

Additionally, we implemented two phases of independent rater evaluations—one to strengthen the credibility of our findings and the other to assess inter-rater reliability.

In the first phase, 25% of answers from each analysis were coded by an independent rater based on the coding system. To enhance the credibility of our findings, any discrepancies and ambiguities identified after the coding process were discussed between the independent coder and one of the authors (LK) until consensus was reached. Based on these findings, the coding system was refined, and the data recoded by one of the authors.

In the second phase, a total of four raters (with a Master degree in psychology) each rated 25% of the data for one analysis. Prior to the rating exercise, each rater underwent a training session that included an introduction to the rating scheme and a discussion to clarify any ambiguities. Afterwards, further ambiguities were discussed and some deviations in coding were analysed and corrected (if they were the result of a misinterpretation of a code or a mistake). We calculated Krippendorff’s Alpha [89] and percent agreement to assess the inter-rater reliability of the coding systems. The resulting Krippendorff’s Alpha coefficients for each analysis overall (.90 - .96) and the percentage agreement for each analysis overall (97.67% − 98.55%) are satisfactory. The coefficients for each code can be found in the supporting information (S10–S13 Tables).

### 6.1. RQ8–9: Motives for the acceptance and rejection of active and passive enhancement methods

We conducted qualitative analyses with MAXQDA 2022 [90]. The motives were assessed in German and examples were translated with the help of DeepL Translator [91] for this article. We analyzed a total of 1182 open-ended responses from 197 participants. The number of responses regarding acceptance or rejection of each method can be found in Table 10. The identified motives reflect the acceptance and rejection of active and passive cognitive enhancement methods in relation to the acceptance rates of each method.

**Table 10:** Number of Open-ended Answers for the Motives for the Acceptance and Rejection of Cognitive Enhancement Methods.

Variable	Acceptance	Rejection
**Passive Enhancement**	**223**	**368**
Brain-Machine-Interface	45	152
Current-based Enhancement	96	101
Pharmacological Enhancement	82	115
**Active Enhancement**	**475**	**116**
Neurofeedback-Training	142	55
Game-based Enhancement	155	42
Working Memory Training	178	19

### 6.2. Motives for the acceptance of passive cognitive enhancement methods

For the acceptance of passive cognitive enhancement methods, we identified a total of 11 main categories. The main categories that were identified in more than 5% of answers with the definition, a representative quote of participants’ answers and the frequency can be found in Table 11. The full code system can be found in S14 Table in the supporting information.

**Table 11:** Main Categories and Frequencies of the Motives for the Acceptance of Passive Enhancement Methods.

Category	Definition	Example	Frequency
Cognitive Abilities	Referring to an improvement of cognitive abilities.	*To improve my mental abilities.*	46.64%
Interest in Enhancement	Referring to an interest in Transhumanism, or the enhancement method.	*(...) I would like to try it out of curiosity.*	23.77%
Utilize in	Addressing an area of life, where the enhancement would yield benefit or that one would want to improve.	*(…) to study for major exams.*	21.08%
Application	Referring positively to the application of the enhancement method.	*It sounds like a method that can improve mental performance without much effort.*	14.80%
Risk-Benefit Analysis	Referring to the evaluation of whether the benefits of the enhancement method outweigh the (potential) costs or risks, or the perception of minimal costs or risks.	*It seems to me that the benefits outweigh the risks.*	13.90%
Well-Being	Referring to positive aspects related to the safety, and health impact of the enhancement method.	*Given that this is a non-invasive method, I could imagine trying it out.*	13.00%
Targeted application	Referencing the temporary effect of the enhancement method or the possibility to apply it selectively.	*Because it can be used as needed (…)*	12.56%
Acquiring new skills	Referencing the acquisition of new skills through enhancement.	*For example, I could learn how to repair a car (…).*	8.97%
Prerequisites	Referring to aspects that must be met or are considered for the acceptance or use of the enhancement method.	*This answer only applies if there are absolutely no safety concerns (…).*	38.12%

*Notes*. *N*_*answers*_ = 223. Frequency = Percentage of answers in which the category occurs.

### 6.3. Motives for the rejection of passive cognitive enhancement methods

Regarding the rejection of passive cognitive enhancement methods, we identified a total of 14 main categories. The main categories that occurred in more than 5% of answers with the definition, a representative quote and the frequency can be found in Table 12. The full code system can be found in the supporting information (S15 Table).

**Table 12:** Main Categories and Frequencies of the Motives for the Rejection of Passive Enhancement Methods.

Category	Definition	Example	Frequency
Health Concerns	Concerns towards potential health effects of enhancement.	*I would have concerns about the side effects.*	45.11%
Safety Concerns	Concerns towards the safety of enhancement or the enhancement method.	*That’s too dodgy for me, sounds creepy (…).*	35.05%
Effort	The application is perceived as overly effortful, time-consuming, impractical, or excessive.	*This would take too much time and effort.*	15.49%
Ethical Considerations	Addressing ethical concerns like availability, loss of humanness, pricing, or criticism of meritocracy either on a personal or societal level.	*(…) if only some have access to it (due to costs, for example), this could lead to serious social problems.*	12.50%
Lack of long-term impact	The lack of sustained improvement through the enhancement method being perceived as a drawback.	*If this effect only lasts for such a short time, I don’t see any advantage in it.*	12.50%
Risk-Benefit Analysis	Referencing that (potential) costs outweigh benefits.	*It certainly has its advantages, but for me the risks and side effects are rather too great.*	8.97%
Unauthentic	Referencing the performance gain as not being based in one’s own abilities, and it therefore being perceived as less valuable, successful, or authentic.	*I would not have the feeling of having “made it” - i.e.,. the feeling of reward, if I could do it (…), but did not owe it to myself.*	8.15%
Illicit	The perception of enhancement similar to (illegal or legal) drugs, doping, cheating or perceiving it as cheating.	*I think that would fall under “cheating” for me (…).*	5.98%
Superior Alternatives	Preference for or perception of other enhancement methods as better.	*There are enough other methods to improve concentration and performance in other ways.*	5.98%
Unnecessary	The belief that an increase in cognitive performance is not necessary or desirable.	*I am satisfied with my current cognitive abilities.*	5.71%
Unnatural	The perception of the performance gain as unnatural or against nature.	*In my opinion everything should be left as natural as possible.*	5.43%
Rejection	Rejection of a specific aspect of the enhancement method or its application.	*In general, I would not be in favour of an unnecessary surgery.*	18.75%
Conditions	Aspects that could lead to acceptance of the enhancement method or reconsideration of its application.	*But if it were to become the norm, I would probably join in just so as not to be left behind.*	12.50%

*Notes*. *N*_*answers*_ = 368. Frequency = Percentage of answers in which the category occurs.

These analyses of motives influencing the acceptance and rejection of passive cognitive enhancement methods revealed a notable overlap between the two. *Health concerns* like *side-effects* or *addiction* and *safety concerns* like a loss of *control* or concerns about *data protection* emerged as the most frequently cited prerequisites for acceptance, and most cited reasons for rejection. Additionally, *ethical considerations* and the *effort* required for passive cognitive enhancement were identified not only as prerequisites for acceptance but also as relevant factors contributing to rejection. We further observed that some individuals who reject passive cognitive enhancement methods would reconsider its application under certain *conditions*. Most noteworthy, people stated that they would reconsider the application of passive cognitive enhancement if it were to be *evidence based*, i.e., rigorously tested or studied, which we also identified as one of the *prerequisites* for acceptance. We further observed that within both the acceptance and rejection of passive cognitive enhancement, some people stated that they would utilize these methods for a *therapeutic application*, which does not align with the definition of enhancement.

### 6.4. Motives for the acceptance of active cognitive enhancement methods

Regarding the acceptance of active cognitive enhancement methods, we identified a total of 13 main categories. The main categories that were identified in more than 5% of answers with the definition, a representative quote and the frequency can be found in Table 13. The full code system can be found in S16 Table in the supporting information.

**Table 13:** Main Categories and Frequencies of the Motives for the Acceptance of Active Enhancement Method.

Category	Definition	Example	Frequency
Cognitive Abilities	Referring to an improvement of cognitive abilities.	*It is always good to increase your intelligence.*	54.32%
Application	Referring to positive aspects about the application of the enhancement method.	*Sounds like a fun way to improve your cognitive skills (…).*	49.47%
Interest in Enhancement	Referring to an interest in enhancement, the enhancement method, its mode of action and effects.	*(…) the idea is, of course, once again ingenious.*	16.63%
Well-Being	Referring to positive aspects related to the safety, and health impact of the enhancement method.	*[The method] sounds harmless to me.*	14.53%
Utilize in	Addressing an area of life, where the enhancement would yield benefit or that one would want to improve.	*(…) and increase success in their academic (…) life.*	15.58%
Targeted application	Referencing the possibility to apply the enhancement selectively regarding which skills to improve or the extent of enhancement, or being in control about the enhancement	*You can also stop [the method] at any time (…).*	8.84%
Risk-Benefit Analysis	Referring to the evaluation of whether the benefits of the enhancement method outweigh the (potential) costs or risks, or the perception of minimal costs or risks.	*(…) the arguments in favour are so overwhelming that it would be almost negligent not to take advantage of this fictitious offer.*	8.21%
Authenticity	Perception of the performance gain being based on one’s own abilities, or it being natural.	*Opportunity to achieve improvement through my own work.*	7.79%
Prerequisites	Referring to aspects that must be met or are considered for the acceptance or use of the enhancement method.	*I would generally make use of it if the effort involved is not too high.*	10.74%

*Notes*. *N*_*answers*_ = 475. Frequency = Percentage of answers in which the category occurs.

### 6.5. Motives for the rejection of active cognitive enhancement Methods

For the acceptance of passive cognitive enhancement methods, we identified a total of 11 main categories. The main categories that occurred in more than 5% of answers with the definition, a representative quote and the frequency can be found in Table 14. The full code system can be found in the supporting information (S17 Table).

**Table 14:** Main Categories and Frequencies of the Motives for the Rejection of Active Enhancement Methods.

Category	Definition	Example	Frequency
Effort	The application being perceived as too effortful, time consuming or impractical.	*I wouldn’t necessarily want to do it because it’s time-consuming.*	41.38%
Safety Concerns	Concerns towards the safety of enhancement or the enhancement method.	*I’m suspicious of anything to do with artificial intelligence, as I’m not sure to what extent it can be controlled (…).*	18.97%
Unnecessary	The belief that an increase in cognitive performance is not necessary or desirable.	*(…) because I am not dissatisfied with my cognitive abilities.*	15.52%
Superior Alternatives	Preference for or perception of other enhancement methods as better.	*[I] would prefer to train my cognitive ability differently.*	7.76%
Risk-Benefit Analysis	Referencing that (potential) costs outweigh benefits.	*The amount of time it takes is not proportional to the benefit.*	7.76%
Ethical Considerations	Addressing ethical concerns like availability, loss of humanness, pricing, or criticism of meritocracy either on a personal or societal level.	*(…) not everyone can afford it (issue of equal opportunities).*	6.03%
Rejection of Video Games^a^	Rejection of video games or virtual reality.	*I wouldn’t play it because I generally don’t like computer games and it would annoy me (…)*	20.69%

*Notes*. *N*_*answers*_ = 116. Frequency = Percentage of answers in which the category occurs.

^a^Category only occurs for Game-based Enhancement.

The present analysis of the motives influencing the acceptance and rejection of active cognitive enhancement methods revealed a noteworthy overlap between the two. The most frequently cited reasons for rejection–*effort* and *safety concerns*–were also the most commonly mentioned *prerequisites* for acceptance. Furthermore, *ethical considerations* were identified not only as a prerequisite for acceptance but also as a reason for rejecting passive cognitive enhancement methods. Furthermore, some individuals who accepted active cognitive enhancement methods expressed *doubts* concerning their ability to follow through with the enhancement, citing concerns about their self-discipline or uncertainty regarding whether they belonged to the target group for specific methods, such as game-based enhancement. Notably, aside from *therapeutic application*, individuals who rejected active methods did not articulate any conditions under which they would reconsider its use, highlighting a key difference from the rejection of passive cognitive enhancement methods.

### 6.6. Comparison of the motives for the acceptance of active and passive cognitive enhancement methods

**Fig 2:**
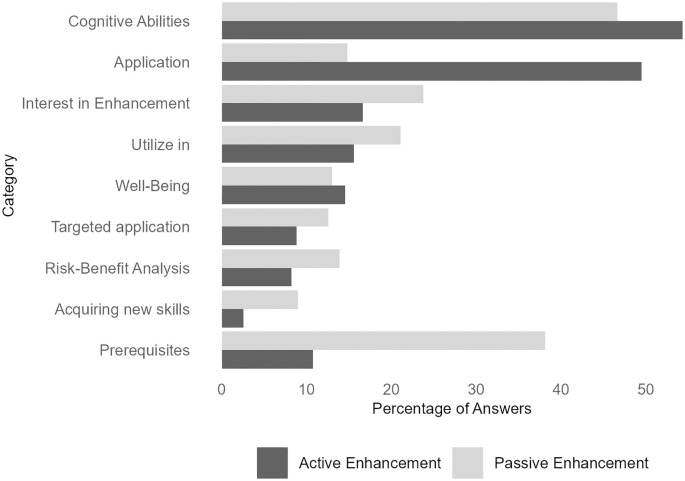
Comparison of main categories identified for the acceptance of active and passive enhancement methods.

The analyses revealed a significant overlap between the motives for accepting active and passive cognitive enhancement methods. A total of nine categories were identified in both analyses, although their frequencies varied (as illustrated in Fig 2).

The *application* of cognitive enhancement methods—referring to positive aspects such as perceiving the process as simple, being familiar with the application, or approving of the method—was mentioned considerably more frequently in the context of accepting active compared to passive cognitive enhancement methods. Another major difference was observed in the *prerequisites* cited for acceptance, including factors like health, safety and ethical concerns, the effort required, or the effectiveness of the cognitive enhancement method. While *prerequisites* were mentioned in 38.12% of responses regarding passive methods, they appeared in only 10.74% of responses concerning active cognitive enhancement methods. Additionally, we noted that *interest in enhancement* was more frequently cited in relation to the acceptance of passive than active cognitive enhancement methods.

### 6.7. Comparison of the motives for the rejection of active and passive cognitive enhancement methods

**Fig 3:**
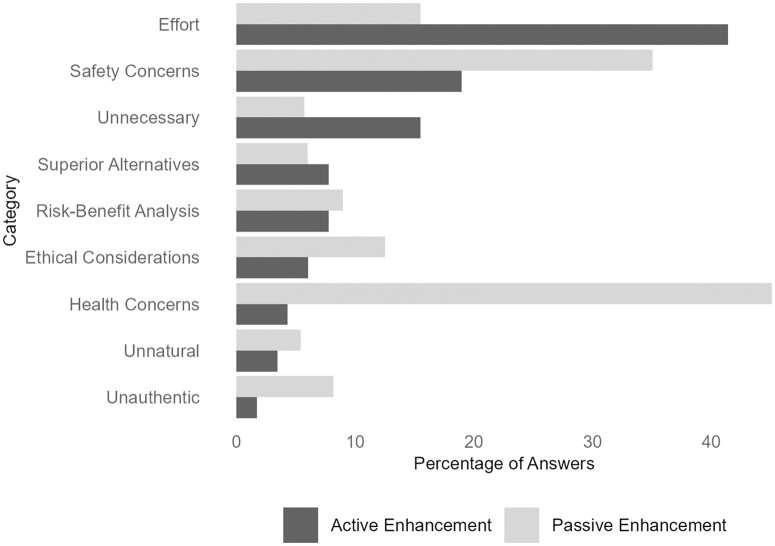
Comparison of main categories identified for the rejection of both active and passive enhancement methods.

We further identified a considerable degree of overlap between the motivations for rejecting active and passive cognitive enhancement methods. A total of nine categories were observed in both analyses, with varying frequencies (see Fig 3).

The most frequently mentioned motives for rejecting passive cognitive enhancement methods—*health* and *safety concerns*—were observed substantially more often in the context of passive than in active cognitive enhancement methods. Specifically, *health concerns*, such as side effects, secondary damage, and addiction, were cited in more than 40% of responses regarding the rejection of passive methods, compared to less than 5% for active cognitive enhancement methods. Additionally, *ethical considerations* —such as issues of availability, cost, or critiques of meritocracy—were raised more often in the context of passive cognitive enhancement methods. The issue of viewing enhancement as *unauthentic* was also deemed more relevant for passive than for active cognitive enhancement methods. In contrast, the most frequently cited motive for rejecting active methods, *effort*, was also mentioned in the context of passive cognitive enhancement, albeit much less frequently. Furthermore, the perception of cognitive enhancement as *unnecessary* was more prevalent in the rejection of active compared to passive cognitive enhancement methods.

## 7. Discussion

The present study follows up and extends initial studies on individual differences as predictors of the acceptance of (i.e., the hypothetical willingness to enhance oneself with) different cognitive enhancement methods [9,39]. We specifically focused on cognitive enhancement, i.e., targeting cognitive domains such as attention, memory, concentration, and, most importantly, intelligence–the trait that is also discussed as the main target of enhancement in many transhumanistic publications. While most cognitive enhancement methods currently seem not effective in enhancing intelligence (for an overview see [6]), their use might entail individual and societal risks–now and in the future [40]. Thus, it is essential to understand who is willing to use which enhancement methods and why.

In line with Grinschgl et al. [9], the two present studies have (by means of exploratory and confirmatory factor analyses) replicated the distinction of active vs. passive cognitive enhancement methods (see RQ1/H1) For active methods a person has to invest effort (like time, cognitive energy, and in general, agency) to a much higher degree than for passive methods, for which agency, time, and effort investments are low or almost nil (like taking a pill). In addition, we replicated the large difference in the acceptance of these two forms of enhancement. We found a higher acceptance for active than passive cognitive enhancement methods with effect sizes of Cohen d’s between 1.39 and 1.52. In Cohen’s terms (with 0.8 as a criterion [92]) such d-values do not only constitute large effects but effect sizes that are rarely found in psychology, if not in all probabilistic sciences [93].

Given these differences, it is important to understand which individuals tend to choose the easier, less effortful—but potentially riskier—form of cognitive enhancement, and which ones prefer the more demanding, effortful approach. Previous studies [9,39] explored associations between intelligence measures, personality traits, values, and science fiction hobbyism and the acceptance of cognitive enhancement methods, but found mostly small effects (*r* < .20) that often vanished in multiple regressions. To broaden the perspective, we included self-esteem and RIASEC interests in Study 1 in addition to intelligence and personality measures, and in Study 2 examined the subfacets of conscientiousness, along with trait anxiety and novelty seeking.

Generally, our findings were similar to previous studies: In Study 1, while no significant correlation was observed between measured general intelligence and the acceptance of passive methods, a small positive correlation was found with active cognitive enhancement methods (see RQ2). However, in the subsequent multiple regression analysis, general intelligence did not predict the acceptance of active cognitive enhancement methods. Similarly, self-estimated intelligence was not significantly related to the acceptance of either passive or active cognitive enhancement methods (see RQ3). Conscientiousness, as the only Big Five trait, correlated negatively with acceptance of passive and active cognitive enhancement (see RQ4; when not correcting for multiple testing, see supplementary S5 Table), but did not explain variance in multiple regressions. The same applies to the Dark triad traits Machiavellianism and grandiose narcissism that also (partially) showed small positive correlations (see H1). From the novel variables included in Study 1 (general interests and self-esteem, see RQ6 and RQ7) only investigative interests correlated significantly with the acceptance of both forms of cognitive enhancement and significantly explained variance in the multiple regression for active but not passive cognitive enhancement. Additionally, conventional interests correlated negatively with the acceptance of active cognitive enhancement (when not correcting for multiple testing, see S5 Table), but did not explain variance in the regression model. As in previous studies [9], science-fiction hobbyism correlated the highest with the acceptance of cognitive enhancement (see H2), but was only a significant predictor for passive, not active cognitive enhancement in multiple regression models. Age explained variance in the acceptance of active cognitive enhancement methods. Younger individuals might be more willing to put the necessary effort into this form of enhancement. Overall, in Study 1, the explained variance was modest, with 13% and 18% for the acceptance of passive and active cognitive enhancement methods, respectively (see RQ8).

Study 2 yielded similar results with rather small effect sizes: Self-estimated intelligence positively correlated with the acceptance of both forms of cognitive enhancement (but no explained variance in the regressions; see RQ2), while psychometrically measured intelligence was only related to the acceptance of active cognitive enhancement (see RQ1). Of the six conscientiousness subfacets, only dutifulness negatively correlated with passive cognitive enhancement methods (when not correcting for multiple testing, see S8 Table) while it did not contribute to the multiple regression models (see H2/RQ3/RQ4). Trait anxiety and novelty seeking were unrelated to the acceptance (see RQ5/RQ6), while Science-Fiction hobbyism was again positively correlated with the highest found correlations (*r* ≥ .30, see H3). While Science-Fiction hobbyism explained variance in both forms of cognitive enhancement, age and psychometric intelligence predicted acceptance of active cognitive enhancement methods: More intelligent and younger people seem more open to methods of active cognitive enhancement, but for passive methods intelligence seems to play no role. Instead, men might be more drawn towards passive methods. Similar to Study 1, the multiple regression models yielded 13% and 19% of explained variance in the acceptance of passive and active cognitive enhancement, respectively (see RQ7).

Taken together, in our studies we were able to replicate previous findings on the large difference in acceptance between passive and active cognitive enhancement methods and certain individual differences (especially Science-Fiction hobbyism) predicting the acceptance of cognitive enhancement methods. However, with the tested individual differences we were only able to explain a small amount of variance in the acceptance of passive and active cognitive enhancement methods.

To gain more insights into people’s views on cognitive enhancement, we investigated them qualitatively. More specifically, we analyzed the motives why individuals would accept or reject cognitive enhancement methods in Study 2. The most frequently stated motive for utilizing both active and passive cognitive enhancement methods was—unsurprisingly—the wish to improve one’s cognitive abilities (see RQ8/RQ9). Beyond the general desire for enhanced cognition, individuals were particularly interested in increasing their productivity (e.g., *“(…) to learn more information in a shorter amount of time.*”) and offsetting perceived deficits, such as reducing forgetfulness. Relatedly, many participants noted that cognitive enhancement could provide tangible benefits in their professional lives, for example stating that it could lead to “*improved academic performance*”. Another important driver was curiosity about enhancement methods, including interest in the methods themselves, their effects (e.g., “*I’d be curious to know if I could actually notice the improvement.”),* or transhumanistic worldviews (e.g., “*I believe this could contribute to the progression of humankind, and I can thus do my part.*”). Additionally, the perceived ease of application was a key factor, particularly for active methods. For instance, participants described these methods as straightforward to use, expressed familiarity with them, or found the process itself—especially in the case of game-based enhancement—enjoyable. Conversely, for both active and passive cognitive enhancement, responses highlighted prerequisites for their acceptance, i.e., conditions that must be met for participants to consider actually using these methods. Participants emphasized aspects such as health and safety, the effort required to apply cognitive enhancement, and ethical considerations, expressing for example that they “*would like to see research on the risks and side effects the [cognitive enhancement method] may have*” However, the frequency of these prerequisites differed substantially: they were mentioned in nearly 40% of responses regarding passive methods, but only in about 10% of responses concerning active cognitive enhancement methods. Notably, these prerequisites overlapped significantly with the motives cited for rejecting cognitive enhancement methods.

The motives for rejecting active and passive cognitive enhancement methods differed considerably. While the most frequently cited reason for rejecting passive methods was health concerns—participants expressed worries about potential side effects, lasting impairments, and addiction—these concerns were far less prevalent for active cognitive enhancement methods. The most frequently mentioned reason for rejecting active methods was, unsurprisingly given the “active” nature of these methods, the significant effort they require, for example stating that it “*would be too much of a hassle*”. Participants described these methods as too time- and labor-intensive, which deterred them from using them. While some participants also viewed passive cognitive enhancement methods as too effortful, this concern was less prevalent than for active methods. Another significant concern regarding both active and passive cognitive enhancement methods was safety. Participants expressed general skepticism about the methods, a desire for more evidence on their safety and efficacy, and fears of losing control over the methods, their effects, or even of themselves—particularly in the case of brain-machine interfaces or game-based enhancement. Further, participants expressed privacy concerns, for example stating “*Big Brother is watching you.*”, as a reference to George Orwell’s novel 1984 [94]. Additionally, many participants rejected specific aspects of cognitive enhancement methods, such as unnecessary surgery for brain-machine interfaces, the use of medication for pharmaceutical enhancement, or video games and virtual reality for game-based enhancement.

Overall, the quantitative findings replicated the strong preference for active over passive cognitive enhancement methods and identified a few consistent individual correlates, such as science-fiction hobbyism, though effect sizes were modest. By complementing these results with qualitative analyses, our mixed-methods approach provided a deeper understanding of why people accept or reject cognitive enhancement methods. The qualitative findings offered rich insights into individuals’ motives for accepting or rejecting cognitive enhancement methods, revealing both practical considerations and underlying beliefs about effort, safety, and ethical acceptability of cognitive enhancement.

### 7.1. Acceptance of cognitive enhancement methods

A main quantitative finding of our study was the considerably higher acceptance of active compared to passive cognitive enhancement methods. This may reflect meritocratic values prevalent in WEIRD (Western, Educated, Industrialized, Rich, and Democratic) countries—i.e., the belief that harder work–such as active cognitive enhancement–deserves to be rewarded. This is also supported by research such as Fitz et al. [95], which found that the public endorses meritocratic principles and the intrinsic value of hard work in the context of cognitive enhancement.

Some of our qualitative findings align with this assumption: for instance, participants viewed passive cognitive enhancement as more inauthentic, while active cognitive enhancement was considered more authentic, with participants stating for example that they would achieve the improvement through “*their own work.*”. However, the most common reason for rejecting active cognitive enhancement methods—the associated effort—contradicts the idea that hard work is universally desired. Even participants who were willing to use active methods expressed doubts about their long-term commitment due to the high level of agency required (e.g., stating “However, I would ultimately fail due to a lack of consistency.”).

The most prevalent reasons for rejecting passive cognitive enhancement—and the most striking differences between active and passive methods in our qualitative analyses—were related to health and safety concerns and the invasiveness of the methods. Consistent with these findings, studies show a higher willingness to utilize cognitive enhancement methods when they are perceived as non-invasive [35] and that individuals are mainly reluctant to use enhancement methods due to concerns about side effects [63].

Other relevant aspects potentially influencing attitudes toward cognitive enhancement, highlighted in previous literature, include coercion, peer pressure, authenticity, and ethical considerations (see [7,29,34] for more elaborate discussions on ethical and societal considerations). While we identified related motives in our analyses—such as viewing passive cognitive enhancement methods as less authentic, less natural, and less fair—these themes were only mentioned in a small number of responses. This was true for both the acceptance and rejection of cognitive enhancement methods analyzed in Study 2, as well as the concerns regarding cognitive enhancement analyzed in Study 1 (see supporting information Table S1 and S2). Maybe these aspects (coercion, peer pressure, fairness, and ethical considerations) are more implicit or potentially less salient when individuals decide whether to utilize cognitive enhancement methods themselves.

Nevertheless, we want to emphasize the importance of (some of) these aspects in shaping the utilization of cognitive enhancement methods. In terms of peer pressure and social influence, the use of enhancement by role models such as celebrities, athletes, or influencers might impact public attitudes. This phenomenon is evident in the rise of weight-loss injections, as well as in cognitive enhancement practices, such as the use of the supplement Methylene Blue. Public interest in Methylene Blue appears to have been sparked by a video showing Robert F. Kennedy Jr. adding a blue substance to his drink [96,97]. Methylene Blue is now being used by many individuals in an unregulated manner, despite not being approved as a supplement in the European Union. This raises significant safety concerns, including risks related to product quality, dose-dependent effects, and drug interactions [98,99].

Some frequently mentioned motives for both active and passive cognitive enhancement methods were related to situational factors such as perceived workload and stress management [58–60]. For instance, we observed a desire to use enhancement to work more efficiently, address perceived deficits, or gain advantages in one’s career or studies. Additionally, for both the rejection of active and passive cognitive enhancement methods, participants often mentioned that they did not view cognitive enhancement as desirable, frequently attributing this to factors such as their already advanced age or their (non-cognitively demanding) occupation. These motives underscore the importance of situational factors—such as one’s occupation, the cognitive demands of one’s environment, and one’s ability to meet those demands—in shaping decisions to utilize enhancement. We therefore recommend that future studies move beyond focusing solely on cognitive abilities and instead assess person-environment fit, specifically the alignment between individuals’ abilities and the cognitive demands of their daily lives.

From a conceptual perspective, the distinction between active and passive enhancement methods may be less clear-cut than our findings suggest. Some methods classified as passive also contain active components (e.g., the training required for brain–machine interfaces), and a framework proposed by Grinschgl et al. [6] conceptualizes active and passive enhancement along a continuum rather than as discrete categories. Although the active/passive distinction proved robust across both studies and replicated previous findings [9], the two-factor solution explains a limited proportion of variance (e.g., 42% in Study 1) and some item loadings are only moderate. Rather than two strictly distinctive factors, there might be a continuum of the required agency for different cognitive enhancement methods, with some active and passive cognitive enhancement methods containing both active and passive elements (see also [6]). Future research should aim at testing such a continuum by, for instance, including an even broader range of cognitive enhancement methods than the present study.

### 7.2. Intelligence and the acceptance of cognitive enhancement

This study examined the relationship of psychometrically measured intelligence and self-estimated intelligence with the acceptance of cognitive enhancement methods through two competing hypotheses: On the one hand individuals with higher intelligence might be more drawn towards cognitive enhancement, suggesting a *rich-get-richer* effect. On the other hand, individuals with lower intelligence might be more drawn towards cognitive enhancement, suggesting a *compensation* effect [40].

In both studies, we observed small to moderate positive correlations between intelligence and the acceptance of active cognitive enhancement–replicating Grinschgl et al. [9]. However, intelligence only predicted acceptance in the regression model for Study 2, not Study 1. These findings provide inconclusive evidence for a potential rich-get-richer effect when it comes to the acceptance of active cognitive enhancement. Research on general attitudes toward enhancement indicates that, at a societal level, cognitive enhancement is viewed as more acceptable for individuals with lower cognitive abilities or a lower performance level [100–102]. Thus, there might be a societal preference for a compensational motive to use cognitive enhancement.

As individuals may struggle to accurately evaluate their own cognitive abilities [43], and people with low cognitive abilities might not be aware of their deficits [69] it could be assumed that the decision to use cognitive enhancement methods is more closely tied to metacognitive beliefs about one’s intelligence, than psychometrically measured intelligence. However, self-estimated intelligence did not predict the acceptance of neither active nor passive cognitive enhancement in both studies–a null finding that was also observed in Grinschgl et al. [9]. Thus, while our study observed inconclusive results with regard to the psychometrically measured intelligence as predictor for active cognitive enhancement, self-estimated intelligence does not seem to act as a predictor. Qualitatively, we observed the offsetting of (perceived) deficits as a motive for the acceptance of enhancement, however not very frequently (7% of answers for passive cognitive enhancement methods, 6% for active cognitive enhancement methods). Similarly, also individuals’ self-esteem was not related to the willingness to use cognitive enhancement methods.

### 7.3. Personality and the acceptance of cognitive enhancement

Following up on previous studies [9], we tested conscientiousness and its subfacets as predictors for the acceptance of cognitive enhancement. While we observed single negative relationships, those factors did not explain variance in either active nor passive cognitive enhancement. Thus, unlike previous studies suggested [9,44,45], conscientiousness does not act as a predictor of cognitive enhancement acceptance in our two studies, neither did other Big 5 and Dark Triad traits. Thus, also traits like Machiavellianism and grandiose narcissism do not seem to predict the willingness to enhance one’s cognition (for similar results see [9,39])–contrary to assumptions that link enhancement motives to self-serving personality profiles.

For individuals with high levels of trait anxiety, rejecting cognitive enhancement could lead to a doubled disadvantage, as proposed by Neubauer [40]. However, our results showed no relationship between trait anxiety and the acceptance of cognitive enhancement methods. Higher anxiety might, on one hand, increase concerns about enhancement (e.g., health and safety concerns) but, on the other hand, could also evoke a fear of being at a disadvantage if enhancement is rejected. Exploring both motivational differences and more situation-specific forms of anxiety in the context of enhancement could help future studies achieve a deeper understanding of these relationships. We further found no relationship between novelty seeking and the acceptance of active or passive cognitive enhancement. Even though some theories suggest that individuals with high levels of novelty seeking may be early adopters of new technologies [54], novelty seeking might not be related to the willingness to enhance cognitive abilities–at least not with the tested enhancement methods. Our qualitative results, however, suggest that novelty (i.e., an interest in the enhancement methods and their effects; 19.73% of answers for passive, 16.63% for active cognitive enhancement methods) is a motive why individuals would consider their application.

### 7.4. Interests and the acceptance of cognitive enhancement methods

In line with previous studies [9], the strongest tested—but still with small effects sizes—predictors for the acceptance of cognitive enhancement were individuals’ interests. Particularly, a higher science fiction hobbyism—i.e., the preference for consuming science-fiction literature, movies, and series—is associated with a greater willingness to pursue enhancement [37,103]. Notably, few participants directly referenced science-fiction media as a motive for applying cognitive enhancement methods. Furthermore, and in contrast to our qualitative findings, when science-fiction media was mentioned, it was mainly in relation to rejecting passive enhancement—particularly by citing negative consequences for humanity.

Interestingly, science-fiction hobbyism did not predict the acceptance of passive cognitive enhancement beyond investigative interests, while the reverse pattern was observed for the acceptance of active cognitive enhancement. One reason for this might be the high correlation between Science-Fiction hobbyism and investigative interests (*r* = .56; see S6 Table), which suggests that these variables share substantial variance. As a result, when both are included in multiple regression models, each accounts for overlapping variance in the outcome, reducing the unique contribution of either predictor. Another reason for this might be that science-fiction hobbyists are more familiar with or receptive to passive forms of cognitive enhancement, as these are often depicted in fictional media (e.g., futuristic technologies, or effortless performance boosts). In contrast, individuals high in investigative interests—those drawn to scientific inquiry and experimentation—may be more inclined toward active forms of cognitive enhancement. Possibly ‘investigative people’ with a high interest in science are more aware of the potential of active rather than passive cognitive enhancement. Potentially, the source of exposure—fictional media versus scientific engagement—may be related to the type of enhancement that individuals find acceptable, though these sources also show a substantial overlap.

Unsurprisingly, none of the other RIASEC interests were significantly related to or predicted the acceptance of cognitive enhancement–likely due to their limited conceptual relevance for motives for cognitive enhancement. Together, these results highlight that interests related to science-fiction exposure and scientific curiosity, may be more relevant predictors of cognitive enhancement acceptance than intelligence and personality traits. Critically, also here the effect sizes were only small.

### 7.5. Limitations and future directions

A central limitation of our study concerns the conceptualization and measurement of “acceptance”. We operationalized acceptance as participants’ hypothetical *willingness* to make use of enhancement methods, assuming these methods were effective in enhancing cognitive performance as described in our study vignettes. This inevitably entails some conceptual ambiguity: our measure may capture a mixture of behavioral intentions, moral attitudes, technological openness, risk–benefit evaluations, perceived realism, or even mere curiosity. We therefore view the current work as an initial step in systematically examining cognitive enhancement acceptance and argue that future research should disentangle these underlying mechanisms more explicitly (e.g., by distinguishing behavioral intentions from moral approval, or by including measures of perceived realism, and risk-benefit evaluations) and use more ecologically-valid measure of the willingness to apply cognitive enhancement methods. A higher ecological validity could, for instance, be achieved by allowing participants to apply the methods (where feasible) and by assessing their motives for utilizing the methods beforehand (and maybe even in hindsight). Relatedly, future research should test the potential continuum of active and passive cognitive enhancement methods (see section ”acceptance of cognitive enhancement methods”).

It also needs to be noted that the internal consistency of the passive enhancement questionnaire (average of three passive cognitive enhancement methods) was rather low in Study 2 (but good in Study 1). This difference was likely introduced due to reducing the questionnaire by one enhancement method (genetic enhancement) in Study 2. However, the results still align with Study 1 and previous research [9], thus, the impact of this limited internal consistency might not be too severe.

Critically, across both studies, we conducted a large number of correlation and regression analyses, thus, chance findings cannot be ruled out. Moreover, some measures (e.g., intelligence and personality instruments) differed between the two studies (which is favorable when aiming at generalizable claims), and some predictors—most notably the psychopathy scale in Study 1—showed relatively low internal consistencies. These issues may have contributed to inconsistencies across studies and should be taken into account when interpreting the results. We therefore place particular emphasis on associations that were observed in both studies and are in line with previous research (e.g., the moderate association of cognitive enhancement acceptance and science-fiction hobbyism [9]).

Furthermore, the present samples primarily consisted of young, well-educated, German-speaking individuals from Austria and Germany, which limits the generalizability of our findings. Future studies should aim to include more diverse samples, particularly with respect to socio-economic status, which is associated not only with differential access to enhancement technologies but also with learning motivation [104]—factors that might shape enhancement acceptance. Should cognitive enhancement methods become more effective in the future, unequal access could further amplify existing disparities in cognitive performance and related outcomes. Attitudes toward enhancement may also differ across cultures, making cross-cultural replications an important next step. Beyond sample characteristics, further methodological limitations—typical for questionnaire studies of this sort—warrant consideration. For instance, acceptance was assessed via self-report, which is susceptible to social desirability bias, particularly given that some enhancement methods might carry stigma.

Based on the observed motives, we propose that future studies could specifically explore the predictive role of person-environment fit in the context of cognitive enhancement, particularly in relation to the cognitive demands of one’s occupation and the challenges of meeting them. While some participants cited motives such as offsetting perceived deficits or increasing efficiency, other individuals rejected enhancement methods because they did not perceive them as necessary, such as those who were already retired. Relatedly, Conrad et al. [105] observed a higher acceptance of enhancement in highly competitive or performance-driven environments, supporting the idea that contextual pressures and social expectations interact with individual motives. Thus, both on a quantitative level (e.g., measurable indicators of person–environment fit such as workload, cognitive strain, or job complexity) and on a qualitative level (e.g., subjective experiences of adequacy, motivation, and perceived necessity), it might be fruitful to examine how discrepancies between cognitive capacity and environmental demands influence not only the likelihood of enhancement use but also its perceived legitimacy and ethical acceptability.

Another factor that may influence the acceptance of especially passive cognitive enhancement methods, as indicated by the results of this study, is the belief in conspiracy theories, as well as a mistrust of technology or companies. Some participant answers were referring to passive cognitive enhancement methods being used to control human thoughts and/or behavior. Also mistrust in technology was reflected in several identified subcategories, including fears related to artificial intelligence, concerns about the safety of personal data, apprehensions about external control, and skepticism toward companies, particularly pharmaceutical companies. Thus, future studies might specifically target conspiracy beliefs and mistrust in technology as a predictor for the acceptance of cognitive enhancement.

## 8. Conclusion

Although we studied a large number of individual differences variables with regard to the acceptance of cognitive enhancement, we can conclude that stable trait variables such as intelligence and personality show only a very limited power to explain the acceptance of both active and passive cognitive enhancement methods. From our novel qualitative findings, we come to the conclusion that likely contextual and situational factors outweigh stable personality dispositions in shaping cognitive enhancement acceptance. In particular, perceived person–environment fit, job demands, and/or social norms may be more strongly related to cognitive enhancement acceptance than personality traits. Furthermore, broad traits may be too general to capture the specific motivational mechanisms involved in enhancement decisions, but future studies could test whether more context-specific factors such as perfectionism, moral flexibility, or mistrust might act as predictors for the acceptance of cognitive enhancement. More specific factors—and potentially broader traits—may interact in complex ways with contextual or situational variables. For example, the influence of intelligence on the acceptance of active versus passive cognitive enhancement methods may vary by age, occupational status, or broader dispositions such as investigative interests. Testing such higher-order interactions, however, will require substantially larger samples, which we aim to pursue in future research.

We also plan to use the qualitative findings to develop questionnaires that more precisely assess enhancement-related motives. The consistent (but rather small) link between science-fiction interest and cognitive enhancement acceptance suggests that additional, highly specific motives may drive people’s desire to enhance themselves—motives that remain largely unexplored. These may include *change motives*, such as whether individuals wish to improve their cognitive abilities at all, or whether they seek other forms of change, for example becoming more extraverted or less neurotic. In this sense, enhancement may need to be viewed more holistically, considering the domains in which people wish to change and the direction of that change.

## Supporting information

S1 Table Categories and Sub-Categories, Definitions, Examples and Frequency of the Concerns Regarding the Application of Active Enhancement Methods in Study 1. (PDF)

S2 Table Categories and Sub-Categories, Definitions, Examples and Frequency of the Concerns Regarding the Application of Passive Enhancement Methods in Study 1. (PDF)

S3 Table Descriptive Statistics multi-item self-estimates in Study 1. (PDF)

S4 Table Correlational Analyses of Intelligence Variables in Study 1. (PDF)

S5 Table Bonferroni-Holm Corrected Correlational Analyses in Study 1. (PDF)

S6 Table Correlation Matrix of Main Variables in Study 1. (PDF)

S7 Table Comparison of the acceptance of the seven enhancement methods in Study 1. (PDF)

S8 Table Bonferroni-Holm Corrected Correlational Analyses in Study 2. (PDF)

S9 Table Correlation Matrix of Main Variables in Study 2. (PDF)

S10 Table Inter-Rater Reliability of the Motives for the Acceptance of Passive Enhancement Methods in Study 2. (PDF)

S11 Table Inter-Rater Reliability of the Motives for the Acceptance of Active Enhancement Methods in Study 2. (PDF)

S12 Table Inter-Rater Reliability of the Motives for the Rejection of Passive Enhancement Methods in Study 2. (PDF)

S13 Table Inter-Rater Reliability of the Motives for the Rejection of Active Enhancement Methods in Study 2. (PDF)

S14 Table Categories and Sub-Categories, Definitions, Examples and Frequency of the Motives for the Acceptance of Passive Enhancement Methods in Study 2. (PDF)

S15 Table Categories and Sub-Categories, Definitions, Examples and Frequency of the Motives for the Rejection of Passive Enhancement Methods in Study 2. (PDF)

S16 Table Categories and Sub-Categories, Definitions, Examples and Frequency of the Motives for the Acceptance of Active Enhancement Methods in Study 2. (PDF)

S17 Table Categories and Sub-Categories, Definitions, Examples and Frequency of the Motives for the Rejection of Active Enhancement Methods in Study 2. (PDF)
