# Supplementary material for: Individual differences and motives for the acceptance of cognitive enhancement: A mixed-methods investigation
Source: PLoS One. 2026 Jul 10;21(7):e0353234. doi: 10.1371/journal.pone.0353234 (PMC13354088; doi:10.1371/journal.pone.0353234)
Supplement: S2 Table — (PDF) [file pone.0353234.s002.pdf]

Table S2

Categories and Sub-Categories, Definitions, Examples and Frequency of the Concerns Regarding the Application of Passive Enhancement Methods in Study 1.

Category	Definition	Example	Frequency	
			Absolut	%
**Health concerns**	Concerns towards potential health effects of enhancement.		**299**	**52.73%**
Side-effects	Concerns towards potential side-effects of enhancement.	*I would have concerns about the side effects.*	124	21.87%
Addiction or Dependence	Concerns towards potentially becoming dependent on the enhancement or developing an addiction.	*I would be afraid to become dependent (…).*	93	16.40%
Secondary damage	Concerns towards potential secondary damage or complications.	*I'm also afraid of permanent damage if [the enhancement method] is faulty at some point.*	111	19.58%
Invasiveness	Concerns about the high degree of invasiveness or excessive physiological/neurological intervention involved in the enhancement.	*I wouldn't do anything invasive to my brain for [enhancement].*	29	5.11%
**Safety Concerns**	Concerns towards the safety of enhancement or the enhancement method.		**117**	**31.22%**
Scepticism	Expressing concerns towards the enhancement method itself, the technology, or AI.	*That's too dodgy for me, sounds creepy (…).*	66	11.64%

Category	Definition	Example	Frequency	
			Absolut	%
Data protection	Concerns towards data safety, being hacked or companies using or stealing data.	*I think I would also be afraid that the super-sensitive data that would presumably be generated in the process would fall into the wrong hands.*	29	5.11%
Evidence	Expressing concerns about insufficient knowledge, information, research, or testing regarding the enhancement method, or a high level of inquiry.	*I am not familiar with [the enhancement method].*	51	8.99%
Control	Concerns towards a potential risk of being externally controlled or experiencing a loss of control over the enhancement, or the enhancement method being manipulated.	*This makes direct control and manipulation of the human mind possible.*	62	10.93%
**Effort**	The application is perceived as overly effortful, time-consuming, impractical, or excessive.	*This would take too much time and effort.*	**168**	**29.63%**
**Ethical Considerations**	Addressing ethical concerns like availability, loss of humanness, pricing, or criticism of meritocracy either on a personal or societal level.	*(…) if only some have access to it (due to costs, for example), this could lead to serious social problems.*	**7**	**1.23%**
**Lack of long-term impact**	The lack of sustained improvement through the enhancement method being perceived as a drawback.	*If this effect only lasts for such a short time, I don't see any advantage in it.*	**12**	**2.12%**

Category	Definition	Example	Frequency	
			Absolut	%
**Risk-Benefit Analysis**	Referencing that (potential) costs outweigh benefits.	*It certainly has its advantages, but for me the risks and side effects are rather too great.*	**7**	**1.23%**
**Superior Alternatives**	Preference for or perception of other enhancement methods as better.	*There are enough other methods to improve concentration and performance in other ways.*	**5**	**0.88%**
**Unnecessary**	The belief that an increase in cognitive performance is not necessary or desirable.	*I am satisfied with my current cognitive abilities.*	**4**	**0.71%**
**Unauthentic**	Referencing the performance gain as not being based in one's own abilities, and it therefore being perceived as less valuable, successful, or authentic.	*I would not have the feeling of having "made it" - i.e. the feeling of reward, if I could do it (...), but did not owe it to myself.*	**17**	**3.00%**
**Unnatural**	The perception of the performance gain as unnatural or against nature.	*In my opinion everything should be left as natural as possible.*	**13**	**2.30%**
**Sci-fi Media**	Referencing science-fiction media, like movies or books.	*For me, that belongs in a science fiction movie.*	**11**	**1.94%**
**Illicit**	The perception of enhancement similar to (illegal or legal) drugs, doping, cheating or perceiving it as cheating.	*I think that would fall under "cheating" for me (...).*	**3**	**0.53%**
**Rejection**	Rejection of a specific aspect of the enhancement method or its application.		**21**	**3.70%**

Category	Definition	Example	Frequency	
			Absolut	%
of Medication [a]	Rejection of (non-medically necessary) medication.	*I am generally critical of taking medication unless it is medically necessary.*	3	0.53%
of Surgery [b]	Rejection of (non-medically necessary) surgeries.	*In general, I would not be in favour of an unnecessary surgery.*	5	0.88%
of Implants [b]	Rejection of (non-medically necessary) implants.	*I would not want to have a chip implanted in me.*	7	1.23%
of Gene-editing [c,d]	Rejection of (non-medically necessary) gen-editing.	*[I have] fundamental concerns about genetic modification in humans (…).*	7	1.23%
**Conditions**	Aspects that could lead to acceptance of the enhancement method or reconsideration of its application.		**23**	**4.06%**
Therapeutic application	The approval of the enhancement method only in case of illness or disability, therefore rejecting its use for enhancement purposes.	*(…) if there is no absolute medical necessity.*	11	1.94%
Evidence-based application	The need for the enhancement method to be rigorously tested or studied or for sufficient information to be available before considering the application.	*I would also wait for further studies and find out about the long-term effect.*	10	1.76%

Category	Definition	Example	Frequency	
			Absolut	%
Preventing Deficits	Considering the application of enhancement to prevent falling behind others who benefit from its advantages.	*But if it were to become the norm, I would probably join in just so as not to be left behind.*	2	.35%

Notes. *N* = 368. Main categories are depicted in bold. Frequency = Number and percentage of answers in which the category occurs.

[a] Category only occurs for pharmacological enhancement.

[b] Category only occurs for brain-machine-interface.

[c] Category only occurs for genetic enhancement.

[d] Categories only occur in Study 1, not Study 2.
