# Supplementary material for: Individual differences and motives for the acceptance of cognitive enhancement: A mixed-methods investigation
Source: PLoS One. 2026 Jul 10;21(7):e0353234. doi: 10.1371/journal.pone.0353234 (PMC13354088; doi:10.1371/journal.pone.0353234)
Supplement: S3 Table — (PDF) [file pone.0353234.s003.pdf]

Table S3

Descriptive Statistics Multi-Item Self-Estimates in Study 1.

Variable	*M*	*SD*	Cronbach's α
**Self-Estimated Intelligence Multi-Item**			
Verbal	3.51	0.60	.85
Numerical	3.10	0.91	.95
Figural	3.27	0.70	.88

Note. N = 203.
