# Supplementary material for: Individual differences and motives for the acceptance of cognitive enhancement: A mixed-methods investigation
Source: PLoS One. 2026 Jul 10;21(7):e0353234. doi: 10.1371/journal.pone.0353234 (PMC13354088; doi:10.1371/journal.pone.0353234)
Supplement: S4 Table — (PDF) [file pone.0353234.s004.pdf]

Table S4

Correlational Analyses of Intelligence Variables in Study 1.

	Passive Enhancement		Active Enhancement	
	r [95% CI]	BF_{01}	r [95% CI]	BF_{01}
**Self-Estimated Intelligence Single-Item (IQ)**				
General	.11 [-.02; .25]	1.72	.11 [-.02; .25]	1.68
Verbal	.07 [-.07; .21]	3.69	.10 [-.04; .24]	2.17
Numerical	.07 [-.07; .21]	3.75	-.03 [-.17; .11]	5.60
Spatial	.10 [-.04; 0.23]	2.29	.03 [-.11; .16]	5.74
**Self-Estimated Intelligence Multi-Item**				
Verbal	<.01 [-.14; .13]	6.11	.11 [-.03; .24]	2.07
Numerical	.04 [-.10; .17]	5.43	-.10 [-.24; .04]	2.21
Figural	.11 [-.02; 0.25]	1.69	-.09 [-.23; .04]	2.58
**Measured Intelligence (z-score)**				
General	.05 [-.09; .18]	4.97	.19** [.06; .32]	0.15
Verbal	.02 [-.12; .15]	5.96	.21** [.08; .34]	0.06
Numerical	.02 [-.12; .15]	5.98	.08 [-.06; .21]	3.50
Spatial	.08 [-.06; .21]	3.44	.17* [.03; .30]	0.36

Note. * $p < .05$. ** $p < .01$. *** $p < .001$. $N = 203$. Confidence intervals depict 95% BCa bootstrapping confidence intervals for 2000 samples. BF_{01} indicates evidence for the null hypothesis over the alternative hypothesis. Psychometrically measured intelligence variables were transformed into z-scores before correlation analysis.
