# Supplementary material for: Individual differences and motives for the acceptance of cognitive enhancement: A mixed-methods investigation
Source: PLoS One. 2026 Jul 10;21(7):e0353234. doi: 10.1371/journal.pone.0353234 (PMC13354088; doi:10.1371/journal.pone.0353234)
Supplement: S5 Table — (PDF) [file pone.0353234.s005.pdf]

Table S5

Bonferroni-Holm Corrected Correlational Analyses in Study 1.

	Passive Enhancement		Active Enhancement	
	r	p_{Holm}	r	p_{Holm}
**Big Five**				
Extraversion	-.01	1.00	-.03	1.00
Agreeableness	-.09	.57	-.01	1.00
Conscientiousness	-.17*	.07	-.16*	.11
Neuroticism	.11	.46	.12	.38
Openness	.02	1.00	.05	1.00
**Dark Triad**				
Machiavellianism	**.20****	**.01**	.13	.14
Psychopathy	.09	.19	< .01	.89
Grandiose Narcissism	**.18***	**.02**	**.19****	**.02**
**General Interests**				
Realistic	.03	1.00	.02	.99
Investigative	.18**	.05	**.26*****	**<.001**
Artistic	<-.01	1.00	.07	.98
Social	-.12	.45	.05	.99
Enterprising	-.07	.91	-.08	.98
Conventional	-.12	.45	-.15*	.17

Note. Bolded correlations are significant under the Bonferroni-Holm correction. Uncorrected p-value: * $p < .05$. ** $p < .01$. *** $p < .001$. $N = 203$.
