# Supplementary material for: Individual differences and motives for the acceptance of cognitive enhancement: A mixed-methods investigation
Source: PLoS One. 2026 Jul 10;21(7):e0353234. doi: 10.1371/journal.pone.0353234 (PMC13354088; doi:10.1371/journal.pone.0353234)
Supplement: S6 Table — (PDF) [file pone.0353234.s006.pdf]

Table S6

Correlation Matrix of Main Variables in Study 1

Variable	1	2	3	4	5	6	7	8	9	10	11	12	13	14	15	16	17	18	19	20	21	22
1 Gender	-																					
2 Age	.11 [-.03–.24]	-																				
3 PEA	.19** [0.05–0.32]	-.10 [-0.24–0.03]	-																			
4 AEA	.09 [-0.05–0.22]	-.29*** [-0.41–-0.16]	.46*** [0.35–0.57]	-																		
5 Intelligence (z-score)	.15* [0.01–0.28]	-.29*** [-0.41–-0.16]	.05 [-0.09–0.18]	.19** [0.06–0.32]	-																	
6 SEI (IQ)	.28*** [0.15–0.41]	-.10 [-0.23–0.04]	.11 [-0.02–0.25]	.11 [-0.02–0.25]	.40*** [0.28–0.51]	-																
7 Extraversion	-.09 [-0.22–0.05]	.09 [-0.05–0.23]	-.01 [-0.15–0.13]	-.03 [-0.17–0.11]	< .01 [-0.14–0.14]	-.08 [-0.21–0.06]	-															
8 Agreeableness	-.18** [-0.31–-0.05]	.17* [0.04–0.30]	-.09 [-0.23–0.05]	-.01 [-0.15–0.12]	-.09 [-0.23–0.05]	-.25*** [-0.37–-0.12]	.35*** [0.22–0.47]	-														
9 Conscientiousness	-.28*** [-0.40–-0.15]	.04 [-0.10–0.18]	-.17* [-0.30–-0.04]	-.16* [-0.29–-0.02]	-.17* [-0.31–-0.04]	-.13 [-0.26–0.01]	.05 [-0.08–0.19]	.11 [-0.03–0.24]	-													
10 Neuroticism	-.06 [-0.20–0.07]	-.19** [-0.32–-0.05]	.11 [-0.03–0.24]	.12 [-0.02–0.25]	-.10 [-0.23–0.04]	-.11 [-0.25–0.02]	-.39*** [-0.50–-0.27]	-.20** [-0.33–-0.07]	-.10 [-0.23–0.04]	-												
11 Openness	.02 [-0.12–0.16]	-.07 [-0.20–0.07]	.02 [-0.12–0.16]	.05 [-0.09–0.19]	-.07 [-0.21–0.07]	-.02 [-0.16–0.12]	.12 [-0.02–0.26]	.06 [-0.08–0.20]	-.07 [-0.20–0.07]	.18** [0.05–0.31]	-											
12 Machiavellianism	.15* [0.02–0.28]	-.07 [-0.20–0.07]	.20** [0.07–0.33]	.13 [-0.01–0.26]	.08 [-0.06–0.21]	.16* [0.02–0.29]	.06 [-0.08–0.20]	-.39*** [-0.50–-0.27]	-.34*** [-0.45–-0.21]	.08 [-0.06–0.22]	.04 [-0.10–0.17]	-										
13 Psychopathy	.36*** [0.24–0.48]	.01 [-0.13–0.15]	.09 [-0.05–0.23]	.01 [-0.13–0.15]	.10 [-0.04–0.24]	.20** [0.06–0.33]	-.21** [-0.34–-0.08]	-.45*** [-0.56–-0.34]	-.32*** [-0.44–-0.19]	.07 [-0.07–0.20]	-.03 [-0.17–0.10]	.50*** [0.38–0.59]	-									
14 Narcissism	.19** [0.06–0.32]	-.20** [-0.33–-0.06]	.18* [0.04–0.31]	.19** [0.05–0.32]	.16* [0.02–0.29]	.17* [0.04–0.31]	.11 [-0.03–0.25]	-.26*** [-0.38–-0.13]	-.23** [-0.35–-0.09]	.05 [-0.09–0.19]	.09 [-0.05–0.23]	.50*** [0.39–0.60]	.38*** [0.26–0.49]	-								
15 Self-Esteem	-.02 [-0.15–0.12]	.31*** [0.18–0.43]	-.06 [-0.20–0.08]	-.06 [-0.20–0.07]	-.07 [-0.20–0.07]	.01 [-0.13–0.15]	.37*** [0.25–0.49]	.24*** [0.10–0.36]	.22** [0.09–0.35]	-.60*** [-0.68–-0.50]	-.09 [-0.23–0.04]	-.07 [-0.20–0.07]	-.06 [-0.19–0.08]	< .01 [-0.14–0.14]	-							
16 Realistic	.44*** [0.32–0.54]	.06 [-0.08–0.20]	.03 [-0.11–0.16]	.02 [-0.12–0.15]	.13 [-0.00–0.27]	.15* [0.02–0.29]	-.10 [-0.24–0.04]	-.07 [-0.20–0.07]	-.02 [-0.16–0.11]	-.05 [-0.19–0.09]	-.05 [-0.18–0.09]	.02 [-0.12–0.16]	.20** [0.06–0.33]	> -.01 [-0.14–0.13]	.02 [-0.12–0.15]	-						
17 Investigative	.33*** [0.20–0.45]	-.09 [-0.23–0.04]	.18** [0.05–0.31]	.26*** [0.13–0.39]	.34*** [0.21–0.45]	.31*** [0.18–0.43]	-.19** [-0.32–-0.06]	-.09 [-0.23–0.04]	-.11 [-0.24–0.03]	.07 [-0.07–0.20]	.12 [-0.02–0.25]	.10 [-0.04–0.23]	.17* [0.03–0.30]	.06 [-0.08–0.20]	-.10 [-0.23–0.04]	.56*** [0.46–0.65]	-					
18 Artistic	-.26*** [-0.38–-0.13]	-.06 [-0.20–0.08]	<-.01 [-0.14–0.14]	.07 [-0.06–0.21]	-.08 [-0.21–0.06]	-.14 [-0.27–0.00]	.15* [0.01–0.28]	.10 [-0.04–0.24]	-.03 [-0.17–0.11]	.17* [0.03–0.30]	.70*** [0.62–0.76]	-.07 [-0.20–0.07]	-.05 [-0.18–0.09]	.02 [-0.12–0.16]	-.05 [-0.19–0.08]	-.07 [-0.21–0.07]	.01 [-0.13–0.15]	-				
19 Social	-.27*** [-0.40–-0.14]	-.03 [-0.17–0.11]	-.12 [-0.25–0.02]	.05 [-0.09–0.18]	.03 [-0.10–0.17]	-.16* [-0.29–-0.02]	.47*** [0.35–0.57]	.31*** [0.18–0.43]	.30*** [0.17–0.42]	-.07 [-0.20–0.07]	.13 [-0.01–0.26]	-.15* [-0.28–-0.01]	-.36*** [-0.48–-0.24]	-.04 [-0.18–0.10]	.12 [-0.01–0.26]	-.21** [-0.34–-0.07]	-.13 [-0.26–0.01]	.26*** [0.13–0.39]	-			
20 Enterprising	.06 [-0.08–0.20]	.06 [-0.08–0.20]	-.07 [-0.21–0.07]	-.08 [-0.22–0.06]	-.03 [-0.17–0.10]	-.01 [-0.15–0.13]	.55*** [0.45–0.64]	.06 [-0.08–0.20]	.12 [-0.02–0.25]	-.33*** [-0.44–-0.20]	-.03 [-0.17–0.11]	.19** [0.05–0.32]	.03 [-0.11–0.17]	.20** [0.06–0.33]	.28*** [0.15–0.41]	.08 [-0.06–0.22]	-.11 [-0.24–0.03]	.03 [-0.11–0.16]	.43*** [0.31–0.53]	-		
21 Conventional	.03 [-0.11–0.16]	.15* [0.01–0.28]	-.12 [-0.25–0.02]	-.15* [-0.28–-0.01]	-.09 [-0.22–0.05]	-.09 [-0.22–0.05]	-.06 [-0.20–0.08]	-.06 [-0.20–0.08]	.27*** [0.14–0.40]	-.11 [-0.25–0.02]	-.19** [-0.32–-0.05]	-.06 [-0.19–0.08]	.11 [-0.03–0.25]	-.03 [-0.17–0.10]	.07 [-0.07–0.21]	.22** [0.08–0.34]	.05 [-0.08–0.19]	-.18* [-0.31–-0.04]	.02 [-0.11–0.16]	.34*** [0.21–0.45]	-	
22 SciFi Hobbyism	.43*** [0.31–0.54]	-.23*** [-0.36–-0.10]	.31*** [0.17–0.43]	.23*** [0.10–0.36]	.14 [-0.00–0.27]	.23** [0.09–0.35]	-.15* [-0.28–-0.01]	-.20** [-0.33–-0.06]	-.21** [-0.33–-0.07]	.09 [-0.04–0.23]	.25*** [0.12–0.38]	.15* [0.01–0.28]	0.28*** [0.15–0.40]	.20** [0.07–0.33]	-.10 [-0.23–0.04]	.37*** [0.24–0.48]	.56*** [0.45–0.65]	.19** [0.05–0.32]	-.22** [-0.35–-0.09]	-.06 [-0.20–0.08]	-.08 [-0.21–0.06]	-

*Note. * p < .05. ** p < .01. *** p < .001. N = 203.* For gender, a value of 0 indicates females and 1 males. Confidence intervals depict 95% BCa bootstrapping confidence intervals for 2000 samples. *PEA = Passive Enhancement Acceptance, AEA = Active Enhancement Acceptance, SEI = Self-Estimated Intelligence, SciFi = Science Fiction.*
