# Supplementary material for: Individual differences and motives for the acceptance of cognitive enhancement: A mixed-methods investigation
Source: PLoS One. 2026 Jul 10;21(7):e0353234. doi: 10.1371/journal.pone.0353234 (PMC13354088; doi:10.1371/journal.pone.0353234)
Supplement: S7 Table — (PDF) [file pone.0353234.s007.pdf]

Table S7

Comparison of the acceptance of the seven enhancement methods in Study 1

Contrast	Estimate	Standard Error	df	*t*	*p*
PhE - CBE	-0.26	0.11	202	-2.35	.412
PhE - GE	0.49	0.10	202	4.76	<.001
PhE - BMI	0.80	0.12	202	6.79	<.001
PhE - WMT	-1.69	0.12	202	-14.59	<.001
PhE - GBE	-1.09	0.12	202	-8.89	<.001
PhE - NFT	-1.11	0.11	202	-9.81	<.001
CBE - GE	0.75	0.11	202	6.98	<.001
CBE - BMI	1.06	0.12	202	9.14	<.001
CBE - WMT	-1.43	0.11	202	-12.76	<.001
CBE - GBE	-0.83	0.11	202	-7.44	<.001
CBE - NFT	-0.85	0.10	202	-8.18	<.001
GE - BMI	0.31	0.11	202	2.86	.097
GE - WMT	-2.18	0.12	202	-18.41	<.001
GE - GBE	-1.58	0.12	202	-12.78	<.001
GE - NFT	-1.60	0.12	202	-13.81	<.001
BMI - WMT	-2.49	0.12	202	-21.05	<.001
BMI - GBE	-1.89	0.12	202	-15.56	<.001
BMI - NFT	-1.91	0.11	202	-17.50	<.001
WMT - GBE	0.60	0.09	202	6.02	<.001
WMT - NFT	0.58	0.09	202	6.21	<.001
GBE - NFT	-0.02	0.10	202	-0.24	1.000

Note. p-values are Bonferroni-corrected. N = 203. BMI = Brain-Machine-Interface, CBE = Current-based Enhancement, PhE = Pharmacological Enhancement, NFT = Neurofeedback-Training, GBE = Game-based enhancement, WMT = Working-Memory Training, CB = Current-based Enhancement.
