# Supplementary material for: Individual differences and motives for the acceptance of cognitive enhancement: A mixed-methods investigation
Source: PLoS One. 2026 Jul 10;21(7):e0353234. doi: 10.1371/journal.pone.0353234 (PMC13354088; doi:10.1371/journal.pone.0353234)
Supplement: S8 Table — (PDF) [file pone.0353234.s008.pdf]

**Table S8**

*Bonferroni-Holm Corrected Correlational Analyses in Study 2.*

| | Passive Enhancement | | Active Enhancement | |
|---|---|---|---|---|
| | *r* | $p_{Holm}$ | *r* | $p_{Holm}$ |
| **Conscientiousness** | -.08 | 1.00 | .04 | 1.00 |
| Competence | -.03 | 1.00 | .06 | 1.00 |
| Order | -.13 | .47 | .05 | 1.00 |
| Dutifulness | -.15* | .24 | .01 | 1.00 |
| Achievement Striving | .06 | 1.00 | .10 | 1.00 |
| Self-Discipline | -.08 | 1.00 | -.08 | 1.00 |
| Deliberation | -.03 | 1.00 | .09 | 1.00 |

*Note.* Bolded correlations are significant under the Bonferroni-Holm correction. Uncorrected p-value: * $p < .05$. ** $p < .01$. *** $p < .001$. $N = 197$.
