# Supplementary material for: Individual differences and motives for the acceptance of cognitive enhancement: A mixed-methods investigation
Source: PLoS One. 2026 Jul 10;21(7):e0353234. doi: 10.1371/journal.pone.0353234 (PMC13354088; doi:10.1371/journal.pone.0353234)
Supplement: S9 Table — (PDF) [file pone.0353234.s009.pdf]

Table S9

Correlation Matrix of Main Variables in Study 2

Variable	1	2	3	4	5	6	7	8	9	10	11	12	13	14	15	16
1 Age	-															
2 Gender[a]	-.06<br>[-.20–.08]	-														
3 AoPE	-.04<br>[-.18–.10]	-.28***<br>[-.41–-.15]	-													
4 AoAE	-.17*<br>[-.30–-.03]	-.20**<br>[-.33–-.06]	.47***<br>[.35–.57]	-												
5 Intelligence	-.07<br>[-.21–.07]	-.11<br>[-.25–.03]	.05<br>[-.09–.19]	.29***<br>[.15–.41]	-											
6 SAI	.01<br>[-.13–.14]	-.24***<br>[-.37–-.11]	.23**<br>[.09–.35]	.20**<br>[.06–.33]	.31***<br>[.18–.43]	-										
7 C	.12<br>[-.02–.25]	.09<br>[-.05–.23]	-.08<br>[-.22–.06]	.04<br>[-.10–.18]	-.06<br>[-.19–.08]	-.01<br>[-.15–.13]	-									
8 C-C	.11<br>[-.03–.25]	-.04<br>[-.18–.10]	-.03<br>[-.17–.11]	.06<br>[-.08–.20]	.04<br>[-.11–.17]	.17*<br>[.03–.30]	.69***<br>[.61–.76]	-								
9 C-O	.01<br>[-.13–.15]	.15*<br>[.01–.28]	-.13<br>[-.26–.01]	.05<br>[-.09–.19]	-.05<br>[-.19–.09]	-.13<br>[-.27–.01]	.77***<br>[.71–.82]	.38***<br>[.25–.49]	-							
10 C-Du	.16*<br>[.02–.29]	.10<br>[-.04–.24]	-.15*<br>[-.28–-.01]	.01<br>[-.13–.15]	-.02<br>[-.16–.12]	-.04<br>[-.18–.10]	.80***<br>[.74–.84]	.49***<br>[.38–.59]	.55***<br>[.45–.64]	-						
11 C-A	-.02<br>[-.16–.12]	.05<br>[-.09–.19]	.06<br>[-.09–.19]	.10<br>[-.04–.24]	-.07<br>[-.21–.07]	.03<br>[-.11–.17]	.72***<br>[.65–.78]	.42***<br>[.29–.53]	.48***<br>[.36–.58]	.48***<br>[.36–.58]	-					
12 C-S	.18*<br>[.04–.31]	.06<br>[-.08–.20]	-.08<br>[-.21–.06]	-.08<br>[-.22–.06]	-.11<br>[-.25–.03]	-.03<br>[-.17–.11]	.86***<br>[.82–.89]	.57***<br>[.47–.66]	.59***<br>[.50–.68]	.66***<br>[.57–.73]	.59***<br>[.50–.68]	-				
13 C-De	.08<br>[-.06–.21]	.08<br>[-.07–.21]	-.03<br>[-.17–.11]	.09<br>[-.05–.23]	> -.01<br>[-.14–.14]	-.01<br>[-.15–.13]	.67***<br>[.58–.74]	.35***<br>[.22–.47]	.45***<br>[.33–.55]	.47***<br>[.35–.57]	.33***<br>[.20–.45]	.41***<br>[.29–.52]	-			
14 Anx	-.20**<br>[-.33–-.06]	.15*<br>[.01–.29]	.04<br>[-.10–.18]	.06<br>[-.08–.20]	.01<br>[-.13–.15]	-.09<br>[-.22–.05]	-.35***<br>[-.47–-.22]	-.55***<br>[-.64–-.45]	-.20**<br>[-.33–-.06]	-.28***<br>[-.40–-.14]	-.13<br>[-.26–-.01]	-.36***<br>[-.48–-.24]	-.09<br>[-.23–.05]	-		
15 NS	-.07<br>[-.21–.07]	-.05<br>[-.19–.09]	.08<br>[-.06–.21]	.09<br>[-.06–.22]	-.02<br>[-.16–.12]	.04<br>[-.10–.18]	-.51***<br>[-.60–-.40]	-.08<br>[-.22–.06]	-.42***<br>[-.53–-.30]	-.44***<br>[-.54–-.32]	-.32***<br>[-.44–-.19]	-.37***<br>[-.48–-.24]	-.63***<br>[-.71–-.54]	-.01<br>[-.15–.13]	-	
16 SciFi	-.03<br>[-.17–.11]	-.33***<br>[-.45–-.20]	.30***<br>[.17–.43]	.32***<br>[.19–.44]	.14*<br>[.00–.28]	.35***<br>[.22–.47]	-.09<br>[-.22–.05]	> -.01<br>[-.14–.14]	-.16*<br>[-.29–-.02]	-.13<br>[-.26–.01]	.01<br>[-.13–.15]	-.09<br>[-.23–.05]	> -.01<br>[-.14–.14]	.07<br>[-.07–.21]	.06<br>[-.08–.20]	-

Notes. *N* = 197, *p* < .05, *p* < .01, *p* < .001. AoPE = Acceptance of passive enhancement, AoAE = Acceptance of active enhancement, IQ = psychometric intelligence, SAI = Self-assessed intelligence, C = Consciousness, C-C = Competence, C-O = Order, C-Du = Dutifulness, C-A = Achievement striving, C-S = Self-discipline, C-De = Deliberation, Anx = Trait Anxiety, NS = Novelty seeking, SciFi = Science Fiction.

[a] *N* = 196 (exclusion of one participant with divers gender identity).
