# Supplementary material for: Individual differences and motives for the acceptance of cognitive enhancement: A mixed-methods investigation
Source: PLoS One. 2026 Jul 10;21(7):e0353234. doi: 10.1371/journal.pone.0353234 (PMC13354088; doi:10.1371/journal.pone.0353234)
Supplement: S10 Table — (PDF) [file pone.0353234.s010.pdf]

Table S10

Inter-Rater Reliability of the Motives for the Acceptance of Passive Enhancement Methods in Study 2.

Category	Krippendorf´s Alpha	Percentage agreement
**Overall**	.952	98.55%
**Cognitive Abilities**	.953	96.43%
Cognitive Enhancement	.862	91.07%
Increase Efficiency	.889	91.07%
Offsetting Deficits	1	100%
Old-Age Provision	1	100%
Preventing Deficits	1	100%
**Interest in Enhancement**	.912	91.07%
Interest in methods	.981	94.64%
Transhumanism	1	100%
**Targeted application**	.923	96.43%
**Risk-Benefit Analysis**	.805	89.29%
**Well-Being**	1	100%
Safety	1	100%
Health	1	100%
Non-Invasive	1	100%
**Acquiring new skills**	.876	96.43%
**Application**	.925	96.43%
Simple	1	100%
Format	.876	96.43%
**Illicit**	.876	98.21%
**Long-term Effectivity**	1	100%
**Utilize in**	.848	94.64%
Career/Academia	1	100%
Everyday life	.876	98.21%
**Prerequisites**	1	100%
Health	1	100%

Category	Krippendorf´s Alpha	Percentage agreement
Side-effects	1	100%
Secondary damage	1	100%
Addiction	1	100%
Effort	1	100%
Safety	.955	96.43%
Safety	1	100%
Data protection	1	100%
Control	1	100%
Evidence-based application	.908	94.64%
Ethical Considerations	1	100%
Therapeutic application	1	100%

Notes. N = 38; $N_{answers}$=56. Main categories are bolded, the further differentiated (second level) sub-categories are written in cursive.
