# Supplementary material for: Individual differences and motives for the acceptance of cognitive enhancement: A mixed-methods investigation
Source: PLoS One. 2026 Jul 10;21(7):e0353234. doi: 10.1371/journal.pone.0353234 (PMC13354088; doi:10.1371/journal.pone.0353234)
Supplement: S11 Table — (PDF) [file pone.0353234.s011.pdf]

Table S11

Inter-Rater Reliability of the Motives for the Acceptance of Active Enhancement Methods in Study 2.

Category	Krippendorf´s Alpha	Percentage agreement
**Overall**	.956	98.21%
**Cognitive Abilities**	.970	95%
General Enhancement	.917	85.83%
Increase Efficiency	.970	98.33%
Optimisation	.848	97.50%
Offsetting Deficits	1	100%
Old-Age Provision	1	100%
Preventing Deficits	1	100%
**Application**	.986	97.50%
Simple	.977	98.33%
Format	.960	95.83%
Fun	1	100%
**Interest in Enhancement**	.855	95%
**Well-Being**	.936	97.50%
Health	1	100%
Safety	.880	97.50%
Non-Invasive	1	100%
**Research**	1	100%
**Authenticity**	1	100%
**Risk-Benefit Analysis**	.903	95%
**Targeted application**	1	100%
**Acquiring new skills**	.912	97.50%
**Long-term effectivity**	1	100%
**Utilize in**	.945	97.50%
Career/Academia	.918	95.83%
Everyday life	1	100%
**Doubts**	1	100%

Category	Krippendorf´s Alpha	Percentage agreement
**Prerequisites**	1	100%
Health	1	100%
Safety	1	100%
Information	1	100%
Scepsis towards AI	1	100%
Data protection	1	100%
Effort	1	100%
Ethical Considerations	1	100%
Effectivity	.879	99.17%

Notes. N = 50; $N_{answers}$=120. Main categories are bolded, the further differentiated (second level) sub-categories are written in cursive.
