# Supplementary material for: Individual differences and motives for the acceptance of cognitive enhancement: A mixed-methods investigation
Source: PLoS One. 2026 Jul 10;21(7):e0353234. doi: 10.1371/journal.pone.0353234 (PMC13354088; doi:10.1371/journal.pone.0353234)
Supplement: S12 Table — (PDF) [file pone.0353234.s012.pdf]

Table S12

Inter-Rater Reliability of the Motives for the Rejection of Passive Enhancement Methods in Study 2.

Category	Krippendorf´s Alpha	Percentage agreement
**Overall**	.936	97.67%
**Health Concerns**	.962	95.65%
Side-Effects	.957	98.91%
Addiction/Dependence	.969	96.74%
Secondary damage	.864	92.39%
Invasiveness	.861	93.48%
**Safety Concerns**	.791	88.04%
Scepticism	.789	86.96%
Data protection	.933	97.83%
Control	1	100%
Evidence	.722	90.22%
**Ethical Considerations**	.911	95.65%
**Effort**	1	100%
**Lack of long-term impact**	.941	96.74%
**Risk-Benefit Analysis**	.918	95.65%
**Superior Alternatives**	1	100%
**Unnecessary**	1	100%
**Unauthentic**	1	100%
**Unnatural**	1	100%
**Illicit**	1	100%
**Sci-fi Media**	1	100%
**Rejection**	.970	96.74%
of Medication [a]	1	100%
of Surgery [b]	.877	96.74%
of Implants [b]	1	100%
**Conditions**	.777	97.83%
Therapeutic application	1	100%

Category	Krippendorf´s Alpha	Percentage agreement
Evidence-based Application	1	100%
Preventing Deficits	1	100%

Notes. N = 43; $N_{answers}$=92. Main categories are bolded.

[a] Category only occurs for pharmacological Enhancement.

[b] Category only occurs for Brain Machine Interface.
