# Supplementary material for: Individual differences and motives for the acceptance of cognitive enhancement: A mixed-methods investigation
Source: PLoS One. 2026 Jul 10;21(7):e0353234. doi: 10.1371/journal.pone.0353234 (PMC13354088; doi:10.1371/journal.pone.0353234)
Supplement: S13 Table — (PDF) [file pone.0353234.s013.pdf]

Table S13

Inter-Rater Reliability of the Motives for the Rejection of Active Enhancement Methods in Study 2.

Category	Krippendorf´s Alpha	Percentage agreement
**Overall**	.901	97.80%
**Effort**	.932	93.10%
**Safety Concerns**	.862	93.10%
Scepticism	.862	93.10%
Information	1	100%
Loss of Reality [a]	1	100%
**Unnecessary**	1	100%
**Superior Alternatives**	1	100%
**Risk-Benefit Analysis**	0	93.10%
**Ethical Considerations**	.649	96.55%
**Health Concerns**	1	100%
**Unnatural**	1	100%
**Unauthentic**	1	100%
**Rejection of Video Games [a]**	.891	96.55%
**Therapeutic application**	0	96.55%

Notes. $N = 21$; $N_{answers}$=29. Main categories are bolded.
[a] Category only occurs for Game-based Enhancement.
