# Supplementary material for: Individual differences and motives for the acceptance of cognitive enhancement: A mixed-methods investigation
Source: PLoS One. 2026 Jul 10;21(7):e0353234. doi: 10.1371/journal.pone.0353234 (PMC13354088; doi:10.1371/journal.pone.0353234)
Supplement: S14 Table — (PDF) [file pone.0353234.s014.pdf]

Table S14

Categories and Sub-Categories, Definitions, Examples and Frequency of the Motives for the Acceptance of Passive Enhancement Methods in Study 2.

Category	Definition	Example	Frequency	
			Absolut	%
**Cognitive Abilities**	Referring to an improvement of cognitive abilities.		**104**	**46.64%**
Cognitive Enhancement	Referring to the pursuit of improved cognitive abilities or performance through enhancement.	*To improve my mental abilities.*	64	28.70%
Increase Efficiency	Referring to an improved workflow, resource savings (e.g., energy or time), and enhanced productivity.	*(…) would make me much more productive.*	35	15.70%
Offsetting Deficits	Addressing potential compensation for (perceived) cognitive deficits.	*(…) to be less forgetful.*	16	7.17%
Old-Age Provision	Addressing the prevention of cognitive decline, or the ability to acquire new skills in later life.	*I could imagine that it could also help older people to maintain their cognitive abilities.*	10	4.48%
Preventing Deficits	Accepting the application of enhancement to prevent falling behind others who benefit from its advantages.	*(…) I would also do it as not to be at a disadvantage compared to others.*	4	1.79%
**Interest in Enhancement**	Referring to an interest in Transhumanism, or the enhancement method.		**53**	**23.77%**

Category	Definition	Example	Frequency	
			Absolut	%
Interest in method	Referring to an interest in the enhancement method, its mode of action and effects.	*(...) I would like to try it out of curiosity.*	44	19.73%
Transhumanism	Addressing transhumanism or the evolution of humanity through enhancement.	*I think transhumanism is exciting and that it could become an important aspect of the future of humanity.*	10	4.48%
**Application**	Referring positively to the application of the enhancement method.		33	14.80%
Simple	Referring to the application of the enhancement method being perceived as simple, easy, or practical.	*It sounds like a method that can improve mental performance without much effort.*	16	7.17%
Format	Referring to the method and its application as positive, e.g. being familiar with the method, or perceiving it as safe or effective.	*There also appear to be no side effects (...).*	19	8.52%
**Risk-Benefit Analysis**	Referring to the evaluation of whether the benefits of the enhancement method outweigh the (potential) costs or risks, or the perception of minimal costs or risks.	*It seems to me that the benefits outweigh the risks.*	31	13.90%
**Well-Being**	Referring to positive aspects related to the safety, and health impact of the enhancement method.		**29**	**13.00%**

Category	Definition	Example	Frequency	
			Absolut	%
Non-Invasive	The enhancement method is perceived as non-invasive, or the physiological/neurological intervention involved in the enhancement are acceptable.	*Given that this is a non-invasive method, I could imagine trying it out.*	24	10.76%
Health	Addressing that the enhancement method does not result in negative health effects, such as side effects, secondary damage, pain, or addiction.	*(…) since I wouldn't be afraid of complications, I would do it.*	9	4.04%
Safety	Perception of the enhancement method as safe or low risk.	*Therefore, it seems (…) less dangerous to me.*	4	1.79%
**Targeted application**	Referencing the temporary effect of the enhancement method or the possibility to apply it selectively.	*Because it can be used as needed (…)*	**28**	**12.56%**
**Acquiring new skills**	Referencing the acquisition of new skills through enhancement.	For example, I could learn how to repair a car (…).	**20**	**8.97%**
**Illicit**	Referencing the perception of enhancement similar to (illegal or legal) drugs, doping or cheating.	*I see it as a kind of doping (…)*	**6**	**2.69%**
**Long-term Effectivity**	Referring to a possible long-term improvement through the enhancement method.	*If the long-term effect is significant, it is definitely worth it.*	**5**	**2.24%**

Category	Definition	Example	Frequency	
			Absolut	%
**Utilize in**	Addressing an area of life, where the enhancement would yield benefit or that one would want to improve.		**47**	**21.08%**
Career/Academia	Benefits in a professional field, work, or university.	*(…) to study for major exams.*	37	16.59%
Everyday life	Benefits in one's everyday life or specifically one's personal life.	*(…) i would consider the possibility of increased performance for (…) private purposes.*	17	7.62%
**Prerequisites**	Referring to aspects that must be met or are considered for the acceptance or use of the enhancement method.		**85**	**38.12%**
Health	Addressing health concerns that are conditional for the acceptance of the enhancement method.		50	22.42%
Side-effects	Addressing side-effects, and the necessity of identifying or	*If there are no severe side effects (…)*	40	17.94%
Secondary damage	Addressing secondary damage, like brain damage or complications.	*Yes, as long as [the method] does not cause damage to the brain.*	19	8.52%
Addiction	The absence of dependence or addiction - whether physical or psychological - to the enhancement method.	*I would take it immediately if it wasn't (…) or addictive.*	9	4.04%

Category	Definition	Example	Frequency	
			Absolut	%
Safety	Addressing safety concerns that are conditional for the acceptance of the enhancement method.		45	20.18%
Safety	Addressing the safety of the enhancement method, emphasizing the need for it to be low-risk or free from significant hazards.	*This answer only applies if there are absolutely no safety concerns (…).*	21	9.41%
Evidence-based application	The need for the enhancement method to be rigorously tested or studied or for sufficient information to be available to actually utilize enhancement.	*I would only use it if there were enough studies/research on it!*	19	8.52%
Control	Referring to a low risk for a loss of control or external control through the enhancement method.	*However, my freedom of choice should still be given.*	8	3.59%
Data protection	Addressing the need for data security, ensuring protection against hacking and unauthorized use by companies.	*However, data protection and the use of my brain data must be precisely fulfilled so that my thoughts cannot be read or stored by third parties.*	5	2.24%
Effort	The enhancement method would only be utilized if the application would be easy and not too time consuming.	*(…) I would use this method as long as [the method] does not take too much time.*	12	5.38%

Category	Definition	Example	Frequency	
			Absolut	%
Ethical Considerations	Addressing ethical concerns that need to be resolved in order to actually utilize enhancement, like availability or pricing, either on a personal or societal level.	*If ethical issues have already been fully resolved (…).*	9	4.04%
Therapeutic application	The approval of the enhancement method only in case of illness or disability, therefore rejecting its use for enhancement purposes.	*[I would use it if] I had a disease that can only be cured by this method.*	3	1.35%

Notes. *N* = 223. Main categories are bolded, the further differentiated (second level) sub-categories are written in cursive. Frequency = Number and percentage of answers in which the category occurs.
