# Supplementary material for: Individual differences and motives for the acceptance of cognitive enhancement: A mixed-methods investigation
Source: PLoS One. 2026 Jul 10;21(7):e0353234. doi: 10.1371/journal.pone.0353234 (PMC13354088; doi:10.1371/journal.pone.0353234)
Supplement: S16 Table — (PDF) [file pone.0353234.s016.pdf]

**Table S16**

*Categories and Sub-Categories, Definitions, Examples and Frequency of the Motives for the Acceptance of Active Enhancement Methods in Study 2.*

| Category | Definition | Example | Frequency | |
|---|---|---|---|---|
| | | | Absolut | % |
| **Cognitive Abilities** | Referring to an improvement of cognitive abilities. | | **258** | **54.32%** |
| Cognitive Enhancement | Referring to the pursuit of improved cognitive abilities or performance through enhancement. | *It is always good to increase your intelligence.* | 200 | 42.11% |
| Increase Efficiency | Referring to an improved workflow, resource savings (e.g., energy or time), and enhanced productivity. | *Because I like to work more efficiently (…).* | 37 | 7.79% |
| Offsetting Deficits | Addressing potential compensation for (perceived) cognitive deficits. | *My short-term memory is terrible.* | 29 | 6.11% |
| Old-Age Provision | Addressing the prevention of cognitive decline, or the ability to acquire new skills in later life. | *Use it or lose it, good for old-age provision.* | 19 | 4.00% |
| Optimisation | Referring to the realization of one's potential or the optimisation/perfection of skills. | *(…) to fully utilise his own "cognitive potential".* | 14 | 2.95% |
| Preventing Deficits | Accepting the application of enhancement to prevent falling behind others who benefit from its advantages. | *I would also be afraid not to take up the offer, as my mental abilities would stagnate, while other people would significantly increase their abilities.* | 2 | 0.42% |

| Category | Definition | Example | Frequency | |
|---|---|---|---|---|
| | | | Absolut | % |
| **Application** | Referring to positive aspects about the application of the enhancement method. | | **235** | **49.47%** |
| Format | Referring to positive aspects of the enhancement method's application, such as approval of the device or familiarity with its use. | *The fact that [the method] can be completed on a mobile phone definitely speaks in favour of this method.* | 156 | 32.84% |
| Simple | Referring to the application of the enhancement method being perceived as simple, easy, or practical. | *This method is not too time-consuming (…).* | 91 | 19.16% |
| Fun | Referring to the potential for fun or entertainment associated with the enhancement method. | *Sounds like a fun way to improve your cognitive skills (…).* | 48 | 10.11% |
| **Interest in Enhancement** | Referring to an interest in enhancement, the enhancement method, its mode of action and effects. | *(…) the idea is, of course, once again ingenious.* | **79** | **16.63%** |
| **Well-Being** | Referring to positive aspects related to the safety, and health impact of the enhancement method. | | **69** | **14.53%** |
| Non-Invasive | The enhancement method is perceived as non-invasive, or the physiological/neurological intervention involved in the enhancement are acceptable. | *(…) is non-invasive. It would therefore be acceptable for me.* | 37 | 7.79% |

| Category | Definition | Example | Frequency | |
|---|---|---|---|---|
| | | | Absolut | % |
| Safety | Perception of the enhancement method as safe or low risk. | *[The method] sounds harmless to me.* | 27 | 5.68% |
| Health | Addressing that the enhancement method does not result in negative health effects, such as side effects, secondary damage, pain, or addiction. | *There are no negative side effects.* | 15 | 3.16% |
| **Targeted Application** | Referencing the possibility to apply the enhancement selectively regarding which skills to improve or the extent of enhancement, or being in control about the enhancement | *You can also stop [the method] at any time (…).*<br>*[I] have it in my own hands.* | **42** | **8.84%** |
| **Risk-Benefit Analysis** | Referring to the evaluation of whether the benefits of the enhancement method outweigh the (potential) costs or risks, or the perception of minimal costs or risks. | *(…) the arguments in favour are so overwhelming that it would be almost negligent not to take advantage of this fictitious offer.* | **39** | **8.21%** |
| **Authenticity** | Perception of the performance gain being based on one's own abilities, or it being natural. | *Opportunity to achieve improvement through my own work.* | **37** | **7.79%** |
| **Acquiring New Skills** | Referencing the acquisition of new skills through enhancement. | *Previously unrecognised skills can be acquired.* | **12** | **2.53%** |
| **Long-Term Effectivity** | Referring to a possible long-term improvement through the enhancement method. | *[The method] has as lasting effect.* | **11** | **2.32%** |

| Category | Definition | Example | Frequency | |
|---|---|---|---|---|
| | | | Absolut | % |
| **Research** | Addressing the enhancement method being well researched or the effectivity proven by science. | *Success is proven by science.* | **6** | **1.26%** |
| **Utilize in** | Addressing an area of life, where the enhancement would yield benefit or that one would want to improve. | | **74** | **15.58%** |
| Career/Academia | Benefits in a professional field, work, or university. | *(…) and increase success in their academic and/or professional life.* | 50 | 10.53% |
| Everyday Life | Benefits in one's everyday life or specifically one's personal life. | *(…) would make everyday life easier.* | 45 | 9.47% |
| **Doubts** | Expressing doubts if one is the target audience for the enhancement method, or if one would actually use it frequently. | *But I'm not sure whether I'm disciplined enough to actually go through with it.* | **19** | **4.00%** |
| **Prerequisites** | Referring to aspects that must be met or are considered for the acceptance or use of the enhancement method. | | **51** | **10.74%** |
| Safety | Addressing safety concerns that are conditional for the acceptance of the enhancement method. | | 21 | 4.21% |
| *Information* | The need for additional information or knowledge about the enhancement method, or further inquiries. | *I would be interested to know exactly how the tasks are set (…).* | 15 | 3.16% |

| Category | Definition | Example | Frequency | |
|---|---|---|---|---|
| | | | Absolut | % |
| *Scepsis towards AI* | Referring to a sceptical perspective on AI and algorithms, including a need for further understanding of their functionality and implications. | *(…) and, above all, there are always the risks associated with algorithms and AIs that need to be considered.* | 5 | 1.05% |
| *Data Protection* | Addressing the need for data security, ensuring protection against hacking and unauthorized use by companies. | *(…) not to collect data about me.* | 5 | 1.05% |
| Effort | The enhancement method would only be utilized if the application would be easy and not too time consuming. | *I would generally make use of it if the effort involved is not too high.* | 17 | 3.58% |
| Effectivity | Addressing the need for the enhancement method to be effective, or more effective than conventional methods. | *(…) however, if solving Sudokus or logic puzzles had the same effect (…), I probably wouldn't do it.* | 17 | 3.58% |
| Ethical Considerations | Addressing ethical concerns that need to be resolved in order to actually utilize enhancement, like availability or pricing, either on a personal or societal level. | *But who decides who gets access to [the enhancement method]?* | 11 | 2.32% |

*Notes*. *N* = 475. Main categories are bolded, the further differentiated (second level) sub-categories are written in cursive. Frequency = Number and percentage of answers in which the category occurs.
