# Supplementary material for: Individual differences and motives for the acceptance of cognitive enhancement: A mixed-methods investigation
Source: PLoS One. 2026 Jul 10;21(7):e0353234. doi: 10.1371/journal.pone.0353234 (PMC13354088; doi:10.1371/journal.pone.0353234)
Supplement: S17 Table — (PDF) [file pone.0353234.s017.pdf]

Table S17

Categories and Sub-Categories, Definitions, Examples and Frequency of the Motives for the Rejection of Active Enhancement Methods in Study 2.

Category	Definition	Example	Frequency	
			Absolut	%
**Effort**	The application being perceived as too effortful, time consuming or unpractical.	*I wouldn't necessarily want to do it because it's time-consuming.*	**48**	**41.38%**
**Safety Concerns**	Concerns towards the safety of enhancement or the enhancement method.		**22**	**18.97%**
Scepticism	Expressing concerns towards the enhancement method itself, the technology, or AI.	*I'm suspicious of anything to do with artificial intelligence, as I'm not sure to what extent it can be controlled (…).*	19	16.38%
Loss of Reality [a]	Concerns about the potential for losing touch with reality as a result of the enhancement.	*I think it makes you lose sight of reality.*	5	4.31%
Information	Expressing concerns about insufficient information or knowledge regarding the enhancement method, or a high level of inquiry.	*(…) and I don't know enough about it.*	3	2.59%
**Unnecessary**	The belief that an increase in cognitive performance is not necessary or desirable.	*(…) because I am not dissatisfied with my cognitive abilities.*	**18**	**15.52%**
**Superior Alternatives**	Preference for or perception of other enhancement methods as better.	*[I] would prefer to train my cognitive ability differently.*	**9**	**7.76%**

Category	Definition	Example	Frequency	
			Absolut	%
**Risk-Benefit Analysis**	Referencing that (potential) costs outweigh benefits.	*The amount of time it takes is not proportional to the benefit.*	**9**	**7.76%**
**Ethical Considerations**	Addressing ethical concerns like availability, loss of humanness, pricing, or criticism of meritocracy either on a personal or societal level.	*(…) not everyone can afford it (issue of equal opportunities).*	**7**	**6.03%**
**Health Concerns**	Concerns towards potential health effects of enhancement, like side-effects, secondary damage or addiction.	*In addition, it is not known whether [the enhancement method] does something harmful to the scalp.*	**5**	**4.31%**
**Unnatural**	The perception of the performance gain as unnatural or against nature.	*[The enhancement] would go against nature, which I think is bad (…).*	**4**	**3.45%**
**Unauthentic**	Referencing the performance gain as not being based in one's own abilities, and it therefore being perceived as less valuable, successful, or authentic.	*(…) I like to achieve my goals/abilities through honest work and effort.*	**2**	**1.72%**
**Rejection of Video Games** [a]	Rejection of video games or virtual reality.	*I wouldn't play it because I generally don't like computer games and it would annoy me (…)*	**24**	**20.69%**

Category	Definition	Example	Frequency	
			Absolut	%
**Therapeutic application**	The approval of the enhancement method only in case of illness or disability, therefore rejecting its use for enhancement purposes.	*If I had a disability, I would accept it and try it out.*	**3**	**2.59%**

Notes. *N* = 116. Main categories are bolded. Frequency = Number and percentage of answers in which the category occurs.

[a] Category only occurs for game-based enhancement.
