# Supplementary material for: Individual differences and motives for the acceptance of cognitive enhancement: A mixed-methods investigation
Source: PLoS One. 2026 Jul 10;21(7):e0353234. doi: 10.1371/journal.pone.0353234 (PMC13354088; doi:10.1371/journal.pone.0353234)
Supplement: Attachment — Submitted filename: IndividualPLOS.pdf [file pone.0353234.s018.pdf]

Individual differences and motives for the acceptance of cognitive enhancement: A mixed-methods investigation

The manuscript investigates the acceptance of cognitive enhancement methods and the individual differences associated with this acceptance. The topic is timely and potentially relevant given the increasing societal interest in enhancement technologies. The inclusion of two preregistered studies, the combination of quantitative and qualitative approaches, and the open-science practices (preregistration, OSF data/code availability) are important strengths of the work. The manuscript is generally well written and theoretically informed. The distinction between active and passive enhancement methods is meaningful and appears empirically supported across both studies. However, the paper also presents several conceptual and methodological weaknesses that should be addressed before publication. In particular, the theoretical framework remains somewhat diffuse, many hypotheses are weakly justified, the manuscript includes a very large number of correlational analyses with relatively small effects, and the interpretation of findings occasionally exceeds the evidential strength of the data. Overall, the manuscript has potential, but substantial revision would improve its clarity, theoretical coherence, and interpretative rigor. I have the following comments:

(1) Theoretical Framework Is Overly Broad and Fragmented. The manuscript combines: intelligence, self-estimated intelligence, Big Five traits, Dark Triad traits, anxiety, novelty seeking, self-esteem, vocational interests, science-fiction hobbyism, and qualitative motives within a single framework. While each variable is individually motivated, the overall theoretical integration is weak. The manuscript often reads as a collection of loosely connected predictors rather than a coherent explanatory model of enhancement acceptance. For example: the rationale linking intelligence to enhancement acceptance remains speculative, novelty seeking is only briefly justified, and several hypotheses appear exploratory despite formal preregistration. The paper would benefit from: a clearer overarching conceptual framework, stronger prioritization of hypotheses, and a sharper distinction between confirmatory and exploratory analyses. Currently, the study risks appearing “kitchen sink”-like, where many variables are tested simultaneously without a sufficiently integrated theoretical rationale.

(2) Multiple Testing and Risk of False Positives. The manuscript conducts a very large number of correlations and regressions across two studies. Although preregistration is commendable, the statistical strategy still raises concerns: dozens of bivariate correlations, multiple regression models, subgroup analyses, exploratory analyses, and qualitative categorizations. Most reported effects are relatively small (e.g., $r \approx .15$–$.30$). In this context, the manuscript should discuss: multiplicity concerns, robustness of effects, and the possibility that some findings may represent chance associations. Importantly, some findings are inconsistent across studies: intelligence predicts active enhancement in

Study 1 but only partially in Study 2, conscientiousness effects vary, several personality effects disappear.
The manuscript currently emphasizes statistically significant findings while underemphasizing inconsistencies.

(3) Interpretation of Effect Sizes Is Sometimes Overstated. Several statistically significant findings correspond to relatively modest effect sizes. For example: intelligence correlations around r = .19, investigative interests around r = .18–.26, science-fiction hobbyism around r = .23–.31. These are meaningful but small-to-moderate associations. However, parts of the discussion occasionally imply stronger psychological importance than warranted. The authors should adopt more cautious language and avoid implying that these traits are major determinants of enhancement acceptance.

(4) Conceptual Ambiguity of "Acceptance". The manuscript defines acceptance as "willingness to make use" of enhancement methods. However, this construct remains somewhat ambiguous: Is it a moral attitude? A behavioral intention? A technological openness trait? A risk-benefit evaluation? Mere curiosity? Especially because many enhancement methods described are highly speculative (e.g., genetic enhancement, brain-machine interfaces), participants' responses may reflect: science-fiction imagination, perceived realism, novelty, or media exposure, rather than genuine behavioral willingness. The manuscript should discuss more explicitly the ecological validity and interpretative limits of these acceptance measures.

(5) Factor Structure Requires More Critical Discussion. The active/passive distinction is interesting and appears replicated. However: the factor solution explains only 42.49% of variance, some loadings are moderate, and the conceptual classification is debatable. For example: neurofeedback contains both active and passive components, brain-machine interfaces arguably involve extensive training, current-based stimulation may vary in user involvement. The manuscript currently treats the two-factor structure as more definitive than warranted.

(6) Qualitative Component Is Underdeveloped. The qualitative analyses are potentially valuable but appear secondary and insufficiently integrated into the manuscript's theoretical contribution. The paper would benefit from: a clearer methodological description of coding procedures, intercoder reliability information, richer illustrative quotations, and deeper integration between qualitative and quantitative findings. At present, the mixed-methods approach feels somewhat additive rather than genuinely integrative.

I also have the following minor comments:

(1) Some sections of the introduction are overly long and could be streamlined. For example, the detailed descriptions of enhancement technologies sometimes resemble review material rather than directly supporting the research questions.
(2) The manuscript occasionally uses speculative or rhetorical language: “replacement of humans by AI,” “superintelligence,” “designer babies,” etc.
These passages could be toned down in a scientific article.
(3) The term “acceptance” is sometimes conflated with actual future behavior.
(4) Some Cronbach’s alpha values are relatively low, especially psychopathy ($\alpha = .55$). This limitation should be discussed more explicitly.
(5) The manuscript would benefit from a stronger distinction between preregistered confirmatory analyses and exploratory follow-up analyses. At several points, exploratory findings are discussed with a level of emphasis similar to preregistered hypotheses.
(6) Some terminology should be standardized throughout the manuscript. For example, the paper alternates between: “cognitive enhancement,” “enhancement,” “enhancement methods,” and “acceptance of enhancement,” which occasionally reduces conceptual precision.
(7) The reporting of Bayes Factors is appreciated, but their interpretation is sometimes minimal. The manuscript could briefly clarify how evidence categories were interpreted (e.g., anecdotal, moderate, strong evidence).
(8) The rationale for excluding the “mind upload” vignette from the original questionnaire could be discussed more critically, since perceived realism may itself be psychologically informative.
(9) Some regression models explain relatively modest proportions of variance (e.g., 13–18%), yet the discussion occasionally implies broader explanatory power than warranted.
(10) The manuscript would benefit from a short limitations paragraph discussing: self-report bias, social desirability, hypothetical responding, and the use of predominantly educated European samples.
(11) The discussion could engage more directly with ethical and societal implications of enhancement acceptance, especially regarding inequality, coercion, and accessibility, which are mentioned in the introduction but less integrated into the interpretation of findings.
(12) Figure and table presentation is generally clear, although some large correlation tables could potentially be moved entirely to supplementary materials to improve readability of the main text.
(13) The manuscript is generally carefully prepared and transparent, particularly regarding preregistration and open-science practices, which constitutes a significant strength of the work.
