# Supplementary material for: Individual differences and motives for the acceptance of cognitive enhancement: A mixed-methods investigation
Source: PLoS One. 2026 Jul 10;21(7):e0353234. doi: 10.1371/journal.pone.0353234 (PMC13354088; doi:10.1371/journal.pone.0353234)
Supplement: Attachment — Submitted filename: Response to Reviewers.pdf [file pone.0353234.s020.pdf]

Reply to the Editor:

Thank you for submitting your manuscript to PLOS ONE and apologies the lengthy process this took. After careful consideration, we feel that it has merit but does not fully meet PLOS ONE's publication criteria as it currently stands. Therefore, we invite you to submit a revised version of the manuscript that addresses the points raised during the review process.

Answer: We thank you for your efforts in coordinating the review process and welcome the opportunity to revise our manuscript in light of your comments and those of the reviewers. Please find our point-by-point responses below, along with the corresponding changes made to the manuscript. We believe these revisions have substantially strengthened our work and hope that it is now suitable for publication in PLOS ONE.

One reviewer has already cautioned you on matters that still persist. While they did not specify, I suspect you know what these are and could at least attempt to address them here. The other reviewer had issue with conceptual, methodological, and statistical clarity.

Answer: Although we cannot determine with certainty what Reviewer 1 was referring to, we believe we have addressed their concern by explicitly acknowledging that the concept of cognitive enhancement has a long history, with discussions dating back centuries (see also our response to Reviewer 1). We are also confident that the conceptual, methodological, and statistical concerns raised by Reviewer 2 have been resolved.

Reply to Reviewer 1:

This looks like an interesting study, the authors have carried out earlier research on the topic and I've reviewed their work before.
However, as I've also told them before, the way they present cognitive enhancement as something new is inaccurate. For example, the references 3 and 4 (line 61) seem odd as evidence that enhancement is "readily applied". There are comprehensive surveys with representative data and even recent books addressing this issue in more detail, also in its social and historical dimension. Time is scarce. I don't have enough time to give these authors the same suggestions over and over again. Good luck with your projects!

Answer: Thank you for your review and your repeated engagement with our work on cognitive enhancement.

In response to your concerns, we have revised several parts of the abstract (see p. 2) and introduction (see p. 3 and 12) to better acknowledge the long history and broader social and historical context of cognitive enhancement. Specifically, we now (a) more clearly describe cognitive enhancement as embedded in a longer-standing discourse, (b) cite additional relevant work that provides a more comprehensive historical and sociological perspective on enhancement (e.g., Schleim, 2023; Schleim & Quednow, 2018), and (c) aim to provide a more nuanced view on the prevalence of cognitive enhancement use.

At the same time, we believe that new technologies, social media influences (including emerging "neuroenhancement bubbles" and self-improvement trends), and the rapid diffusion of biomedical methods (e.g., GLP-1 agonist hype) are currently reshaping the enhancement landscape. These developments might accelerate the wider use of cognitive enhancement methods.

Reply to Reviewer 2:

The manuscript investigates the acceptance of cognitive enhancement methods and the individual differences associated with this acceptance. The topic is timely and potentially relevant given the increasing societal interest in enhancement technologies. The inclusion of two preregistered studies, the combination of quantitative and qualitative approaches, and the open-science practices (preregistration, OSF data/code availability) are important strengths of the work. The manuscript is generally well written and theoretically informed. The distinction between active and passive enhancement methods is meaningful and appears empirically supported across both studies. However, the paper also presents several conceptual and methodological weaknesses that should be addressed before publication. In particular, the theoretical framework remains somewhat diffuse, many hypotheses are weakly justified, the manuscript includes a very large number of correlational analyses with relatively small effects, and the interpretation of findings occasionally exceeds the evidential strength of the data. Overall, the manuscript has potential, but substantial revision would improve its clarity, theoretical coherence, and interpretative rigor. I have the following comments:

Answer: We are grateful for your thorough review of our manuscript. We were pleased to hear that you consider the research timely and that our open science practices were well received. Your comments have been carefully considered, and we have revised the manuscript accordingly. Most importantly, we are now more thoroughly explaining the exploratory nature of our research and have carefully revised the interpretation of our findings. Please see our point-by-point responses below.

(1) Theoretical Framework Is Overly Broad and Fragmented. The manuscript combines: intelligence, self-estimated intelligence, Big Five traits, Dark Triad traits, anxiety, novelty seeking, self-esteem, vocational interests, science-fiction hobbyism, and qualitative motives within a single framework. While each variable is individually motivated, the overall theoretical integration is weak. The manuscript often reads as a collection of loosely connected predictors rather than a coherent explanatory model of enhancement acceptance. For example: the rationale linking intelligence to enhancement acceptance remains speculative, novelty seeking is only briefly justified, and several hypotheses appear exploratory despite formal preregistration. The paper would benefit from: a clearer overarching conceptual framework, stronger prioritization of hypotheses, and a sharper distinction between confirmatory and exploratory analyses. Currently, the study risks appearing "kitchen sink"-like, where many variables are tested simultaneously without a sufficiently integrated theoretical rationale.

Answer: Thank you very much for this thoughtful and constructive comment. We appreciate the opportunity to clarify our theoretical rationale.

Our overarching aim with this work was to provide an initial, systematic mapping of individual-difference correlates of cognitive enhancement acceptance. Because the literature on psychological predictors of cognitive enhancement acceptance is still emerging, often inconsistent and mostly focused on single cognitive enhancement methods, we deliberately adopted an exploratory approach. This approach combined (a) variables that have been conceptually or empirically linked to enhancement in previous work (e.g., personality traits, intelligence and science-fiction hobbyism) and (b) less frequently, but conceptually plausible, examined predictors (e.g., novelty seeking and trait anxiety). Our intention was to establish a broader empirical foundation on which more focused theoretical models can be built. We therefore refrained from constructing a post-hoc explanatory framework, keeping the exploratory rationale closely aligned with our preregistered research questions and hypotheses. We clarified this aim of our study on p. 11 of the introduction:

"*Research on the psychological predictors of cognitive enhancement acceptance is still emerging, often inconsistent, and mostly focused on single enhancement methods. The present study therefore examines a range of individual difference variables: those conceptually or empirically linked to cognitive enhancement acceptance in previous work, such as personality traits, intelligence, and*

science-fiction hobbyism, as well as less frequently examined but conceptually plausible predictors such as novelty seeking and trait anxiety. The goal is to establish a broader empirical foundation from which more focused theoretical models on the predictors of cognitive enhancement acceptance can be developed in the future."

Furthermore, we now more explicitly elaborate on why we tested intelligence and novelty seeking as predictors. See p.8 and 10:

"First, we aimed at taking a closer look at intelligence and self-estimated intelligence, as cognitive enhancement methods explicitly target cognitive abilities, like intelligence. For this link, two hypotheses have been proposed [9,40]: the rich-get-richer hypothesis and the compensation hypothesis. The rich-get-richer hypothesis proposes that individuals with high cognitive abilities would benefit more from cognitive enhancement and might also be more drawn towards cognitive enhancement (see also 'Matthew effect' [41]). Their cognitive abilities would increase, further distinguishing them from individuals with lower cognitive abilities. The compensation hypothesis proposes the opposite effect. People with lower cognitive abilities would benefit more from cognitive enhancement and might be more drawn towards it, resulting in an equalizing effect. This would alter the distribution of intelligence in the population, rendering cognitive ability less important due to the reduced variance [40] (see also reverse Matthew effect [42])."

"Furthermore, in this study we also tested novelty seeking as a potential predictor of the acceptance of cognitive enhancement methods. A study from Maier et al. [53] found a positive relationship between novelty seeking and the use of pharmacological enhancement and people with high levels of novelty seeking may be early adopters of new technologies [54]. They further show a higher risk preference [55], and higher delay discounting (i.e., a higher preference for a smaller, immediate reward over a bigger, delayed rewards [56]), potentially indicating a positive relationship with the acceptance of (specifically passive) cognitive enhancement methods."

Taken together, we agree that a more elaborate, tightly integrated theoretical framework would be valuable and is a key next step for the field. At the same time, we believe that mapping out which individual characteristics (e.g., personality, intelligence, interests) do or do not relate to cognitive enhancement acceptance is a necessary foundation for such a framework. As stated in our Discussion, we describe how our results suggest that individual differences alone may not be sufficient to explain cognitive enhancement acceptance, and we explicitly call for future work that integrates these characteristics with situational and contextual factors. In our revised manuscript, we further elaborate on this need for a more comprehensive theoretical framework and clarify how our findings can inform it.

Finally, we now more clearly distinguish our analyses between confirmatory, planned exploratory, and follow-up exploratory analyses in line with our preregistrations in the result sections of our manuscript. For more on this, please see our response to your minor comment nr. 5.

(2) Multiple Testing and Risk of False Positives. The manuscript conducts a very large number of correlations and regressions across two studies. Although preregistration is commendable, the statistical strategy still raises concerns: dozens of bivariate correlations, multiple regression models, subgroup analyses, exploratory analyses, and qualitative categorizations. Most reported effects are relatively small (e.g., $r \approx .15–.30$). In this context, the manuscript should discuss: multiplicity concerns, robustness of effects, and the possibility that some findings may represent chance associations. Importantly, some findings are inconsistent across studies: intelligence predicts active enhancement in Study 1 but only partially in Study 2, conscientiousness effects vary, several personality effects disappear.
The manuscript currently emphasizes statistically significant findings while underemphasizing inconsistencies.

Answer: Thank you for raising this important point. We agree that the large number of correlations and regression analyses, combined with relatively small effect sizes, raises concerns about multiplicity and the robustness of individual findings.

We now apply exploratory Bonferroni–Holm corrections to selected sets of correlations (see Supporting Material Tables S5 and S8) so that readers can better judge the robustness of these associations under stricter multiple-testing control. More specifically, we applied corrections when variables shared the same measurement instrument, such as Big Five, Dark Triad, and interest questionnaires. Under this correction, some associations with enhancement and acceptance (e.g., with Machiavellianism and narcissism in Study 1) appear relatively robust, whereas others (e.g., with Conscientiousness in Study 1 and its sub-facet Dutifulness in Study 2) are less stable and should be interpreted more cautiously.

Importantly, to follow our preregistered analysis plan, we continued to select predictors for the regression models based on uncorrected bivariate significance. We chose this approach to avoid an overly conservative procedure that could substantially increase Type II error and obscure potentially meaningful, but small, effects, particularly given the mostly exploratory nature of our research questions. However, the possibility of chance findings can, of course, not be fully ruled out. We therefore added a limitations paragraph explicitly addressing these issues (see p. 60):

"Critically, across both studies, we conducted a large number of correlation and regression analyses, thus, chance findings cannot be ruled out. Moreover, some measures (e.g., intelligence and personality instruments) differed between the two studies (which is favorable when aiming at generalizable claims), and some predictors—most notably the psychopathy scale in Study 1—showed relatively low internal consistencies. These issues may have contributed to inconsistencies across studies and should be taken into account when interpreting the results. We therefore place particular emphasis on associations that were observed in both studies and are in line with previous research (e.g., the moderate association of cognitive enhancement acceptance and science-fiction hobbyism [9])."

We also highlight that some measures (e.g., the intelligence and personality assessments) differed between Studies 1 and 2 (which is an advantage when aiming at generalizing claims), and that single predictors—most notably the psychopathy scale in Study 1—showed relatively low internal consistency. We acknowledge that these measurement issues may have contributed to inconsistencies across studies and that some effects might be due to random variation. Accordingly, across our whole manuscript, we now emphasize more clearly that the observed effects are generally small and, in some cases, inconclusive (e.g., associations found only in one of the two studies). Most importantly, we place stronger interpretative weight on those associations that were observed consistently across both studies and align with previous work (e.g., the moderate association of cognitive enhancement acceptance and science-fiction hobbyism).

(3*) Interpretation of Effect Sizes Is Sometimes Overstated. Several statistically significant findings correspond to relatively modest effect sizes. For example: intelligence correlations around r = .19, investigative interests around r = .18–.26, science-fiction hobbyism around r = .23–.31. These are meaningful but small-to-moderate associations. However, parts of the discussion occasionally imply stronger psychological importance than warranted. The authors should adopt more cautious language and avoid implying that these traits are major determinants of enhancement acceptance.*

Answer: In the revised manuscript, we have adopted more cautious language when interpreting our findings and now more explicitly acknowledge that the observed effect sizes are mostly small to moderate. For instance, we now also explicitly mention this in the abstract (see p. 2). Additionally, we also place stronger interpretative weight on those associations that were observed consistently across both studies and that align with previous work (e.g., the moderate association of cognitive enhancement acceptance and science-fiction hobbyism). For more on this, please see our response to your major comment nr. 2.

(4) Conceptual Ambiguity of "Acceptance". The manuscript defines acceptance as "willingness to make use" of enhancement methods. However, this construct remains somewhat ambiguous: Is it a moral attitude? A behavioral intention? A technological openness trait? A risk-benefit evaluation? Mere curiosity? Especially because many enhancement methods described are highly speculative (e.g.,

genetic enhancement, brain-machine interfaces), participants' responses may reflect: science-fiction imagination, perceived realism, novelty, or media exposure, rather than genuine behavioral willingness. The manuscript should discuss more explicitly the ecological validity and interpretative limits of these acceptance measures.

Answer: We thank the reviewer for this helpful comment and agree that the construct of "acceptance" was conceptually ambiguous in our original manuscript. In the revised manuscript, we now explicitly clarify that we operationalized acceptance as participants' *hypothetical* willingness to make use of enhancement methods as described in our study vignettes. We also acknowledge—and agree with you—that this measure might capture and be shaped by different aspects, such as behavioral intentions, moral attitudes and perceived realism. While our research did not focus on testing the underlying mechanisms of cognitive enhancement acceptance, we now also explicitly highlight that future studies should disentangle these mechanisms more explicitly.

These points have been incorporated into the Limitations and future direction section as follows (see p. 59):

"A central limitation of our study concerns the conceptualization and measurement of "acceptance". We operationalized acceptance as participants' hypothetical willingness to make use of enhancement methods, assuming these methods were effective in enhancing cognitive performance as described in our study vignettes. This inevitably entails some conceptual ambiguity: our measure may capture a mixture of behavioral intentions, moral attitudes, technological openness, risk–benefit evaluations, perceived realism, or even mere curiosity. We therefore view the current work as an initial step in systematically examining cognitive enhancement acceptance and argue that future research should disentangle these underlying mechanisms more explicitly (e.g., by distinguishing behavioral intentions from moral approval, or by including measures of perceived realism, and risk-benefit evaluations) and use more ecologically-valid measure of the willingness to apply cognitive enhancement methods"

(5) Factor Structure Requires More Critical Discussion. The active/passive distinction is interesting and appears replicated. However: the factor solution explains only 42.49% of variance, some loadings are moderate, and the conceptual classification is debatable. For example: neurofeedback contains both active and passive components, brain-machine interfaces arguably involve extensive training, current-based stimulation may vary in user involvement. The manuscript currently treats the two-factor structure as more definitive than warranted.

Answer: Thank you for raising this concern. We agree that the factor structure should be discussed with more caution. Thus, we have added a paragraph to the discussion ("acceptance of cognitive enhancement methods") in which we explicitly address the limited explained variance, the moderate loadings of some items, and the conceptual ambiguity of the active/passive classification for certain methods. In this paragraph, we also acknowledge that, although the active/passive distinction appears robust across our two studies and replicates previous work by our lab, the two-factor solution should be regarded as a preliminary framework that needs further refinement in future research:

"From a conceptual perspective, the distinction between active and passive enhancement methods may be less clear-cut than our findings suggest. Some methods classified as passive also contain active components (e.g., the training required for brain–machine interfaces), and a framework proposed by Grinschgl et al. [6] conceptualizes active and passive enhancement along a continuum rather than as discrete categories. Although the active/passive distinction proved robust across both studies and replicated previous findings [9], the two-factor solution explains a limited proportion of variance (e.g., 42% in Study 1) and some item loadings are only moderate. Rather than two strictly distinctive factors, there might be a continuum of the required agency for different cognitive enhancement methods, with some active and passive cognitive enhancement methods containing both active and passive elements (see also [6]). Future research should aim at testing such

a continuum by, for instance, including an even broader range of cognitive enhancement methods than the present study."

(6) Qualitative Component Is Underdeveloped. The qualitative analyses are potentially valuable but appear secondary and insufficiently integrated into the manuscript's theoretical contribution. The paper would benefit from: a clearer methodological description of coding procedures, intercoder reliability information, richer illustrative quotations, and deeper integration between qualitative and quantitative findings. At present, the mixed-methods approach feels somewhat additive rather than genuinely integrative.

Answer: Thank you for bringing this to our attention. Regarding the methodological description of coding procedures, we aimed to describe these procedures as transparently and detailed as possible, while at the same time keeping readability and conciseness in mind. We therefore refrained from adding additional information to the main manuscript, but can still do so if the reviewer thinks that this adds value to the manuscript

Regarding intercoder reliability information, we would kindly like to refer you to the supporting information (Table S10-S13), where we provide intercoder reliability information for each code in each analysis (see also p. 40 in the manuscript). We calculated Krippendorff's Alpha and percent agreement to assess the inter-rater reliability of each analysis in Study 2. The resulting Krippendorff's Alpha coefficients for each analysis overall (.90 - .96) and the percentage agreement for each analysis overall (97.67% - 98.55%) are good.

We fully agree that the integration of the qualitative and quantitative components can be deepened. In the revised manuscript, we have therefore explicitly strengthened this integration in the Discussion, for example by adding richer illustrative quotations and more clearly connecting the qualitative themes with our quantitative results and with the existing literature (see for example p. 52). We hope that these changes make the mixed-methods contribution more cohesive and theoretically informative.

I also have the following minor comments:
(1) Some sections of the introduction are overly long and could be streamlined. For example, the detailed descriptions of enhancement technologies sometimes resemble review material rather than directly supporting the research questions.

Answer: We agree that the introduction was rather long, and, in the revised manuscript, we have streamlined several sections to make it more concise. More specifically, we shortened the descriptions of cognitive enhancement methods (see p. 5-6) and, for details on effectiveness, refer readers to the recent review by Grinschgl et al. (2025).

(2) The manuscript occasionally uses speculative or rhetorical language: "replacement of humans by AI," "superintelligence," "designer babies," etc.
These passages could be toned down in a scientific article.

Answer: We agree that these terms sound rather rhetorical, but nevertheless play a prominent role in current public discussions. Thus, we believe that we need to make reference to them. Whenever useful, we have used quotation marks to indicate their rhetorical nature.

(3) The term "acceptance" is sometimes conflated with actual future behavior.

Answer: We agree that some formulations were indeed not fitting, and we have adapted the wording in the revised manuscript to clearly refer to acceptance rather than actual future behavior. Please also consider our response to major comment (4) regarding this topic.

(4) Some Cronbach's alpha values are relatively low, especially psychopathy ($\alpha = .55$). This limitation should be discussed more explicitly.

Answer: We agree that the relatively low internal consistency of single scales represents an important limitation. In the revised manuscript, we now address this more explicitly in the limitations

section and caution that low reliability (esp. for the variable psychopathy) may have impacted associations and contributed to inconsistent findings across studies:

"It also needs to be noted that the internal consistency of the passive enhancement questionnaire (average of three passive cognitive enhancement methods) was rather low in Study 2 (but good in Study 1). This difference was likely introduced due to reducing the questionnaire by one enhancement method (genetic enhancement) in Study 2. However, the results still align with Study 1 and previous research [9], thus, the impact of this limited internal consistency might not be too severe.

Critically, across both studies, we conducted a large number of correlation and regression analyses, thus, chance findings cannot be ruled out. Moreover, some measures (e.g., intelligence and personality instruments) differed between the two studies (which is favorable when aiming at generalizable claims), and some predictors—most notably the psychopathy scale in Study 1—showed relatively low internal consistencies. These issues may have contributed to inconsistencies across studies and should be taken into account when interpreting the results. We therefore place particular emphasis on associations that were observed in both studies and are in line with previous research (e.g., the moderate association of cognitive enhancement acceptance and science-fiction hobbyism [9])."

Please also refer to our responses to your Major Comments 2 and 3, as the issues of measurement reliability, multiple testing, and small effect sizes are closely related and are now discussed together to provide a more nuanced interpretation of our results.

(5) The manuscript would benefit from a stronger distinction between preregistered confirmatory analyses and exploratory follow-up analyses. At several points, exploratory findings are discussed with a level of emphasis similar to preregistered hypotheses.

Answer: We thank you for this comment, as this clarification will help readers better understand our analytical rationale. In our revised manuscript, we have clarified that some of our analyses were planned exploratory analyses, preregistered as such due to limited or conflicting prior evidence and theoretical conceptualization. In the revised manuscript, we (a) explicitly label these analyses as planned exploratory and (b) more clearly distinguish them from preregistered confirmatory tests and exploratory follow-up analysis, which were clearly labeled being not mentioned in our preregistration (see Table 1 and results section of Study 1 and Study 2).

(6) Some terminology should be standardized throughout the manuscript. For example, the paper alternates between: "cognitive enhancement," "enhancement," "enhancement methods," and "acceptance of enhancement," which occasionally reduces conceptual precision.

Answer: Thank you for bringing this to our attention. We agree that some terminology was used interchangeably, which may (1) reduce conceptual precision and (2) lead to possible misinterpretations. In our revised manuscript, we have adapted the wording so that it is clear when we refer to cognitive enhancement in general, to specific cognitive enhancement methods, and to their acceptance. We are confident that these changes improve the conceptual precision of the manuscript.

(7) The reporting of Bayes Factors is appreciated, but their interpretation is sometimes minimal. The manuscript could briefly clarify how evidence categories were interpreted (e.g., anecdotal, moderate, strong evidence).

Answer: In the revised manuscript, we now clarify what our Bayes factors test. We thank the reviewer for making us aware that our original description of the Bayesian analyses was not sufficiently transparent:

"Besides calculating Pearson correlations, we also calculated preregistered Bayes Factors for these analyses. These Bayes Factors serve as complementary evidence in favor of the null over the alternative hypothesis and were computed using a default Jeffreys-type Bayesian test for linear

correlation [75], with noninformative priors on the population means and variances and a symmetric, and a weakly informative prior on the correlation coefficient under the alternative hypothesis."

We specifically report BF as an index of evidence in favor of the null hypothesis over an alternative model, using the default priors implemented in the BayesFactor package in R. We also emphasize that, because we did not specify tailored informative priors for each hypothesis, these Bayes factors are intended as complementary to the frequentist analyses rather than as the primary basis for inference. Furthermore, in our results section (see p. 26 and 36) we now mention when Bayes Factors were rather high, thus, favoring the null hypothesis.

(8) The rationale for excluding the "mind upload" vignette from the original questionnaire could be discussed more critically, since perceived realism may itself be psychologically informative.

Answer: Thank you for this comment. We agree that this decision should be explained more clearly. As we clarify in the revised manuscript, we excluded the "mind upload" vignette because it was not framed to enhance one's cognitive functioning during one's lifetime, but rather to preserve one's mind without a physical body (e.g., beyond physical death). Since our focus in this study is specifically on methods that involve the enhancement of cognitive functioning in the current self, we decided to exclude this vignette from the questionnaire (see p. 19):

"*We excluded the vignette for mind upload, as it does not describe the enhancement of one's cognitive functioning during one's lifetime, but rather the preservation or continuation of the mind without a physical body (i.e., beyond physical death)."*

(9) Some regression models explain relatively modest proportions of variance (e.g., 13–18%), yet the discussion occasionally implies broader explanatory power than warranted.

Answer: We agree that some regression models explain only modest proportions of variance. In the revised manuscript, we have adjusted our wording in the Discussion (and also the abstract) to better reflect these small effect sizes and to avoid implying broader explanatory power than warranted. Please also see our response to your Major Comments 2 and 3, where we address related issues concerning multiple testing, small effect sizes, and the robustness of findings.

(10) The manuscript would benefit from a short limitations paragraph discussing: self-report bias, social desirability, hypothetical responding, and the use of predominantly educated European samples.

Answer: Thank you for this helpful comment. We now more explicitly address the use of young, highly educated samples, particularly in light of our argument that socio-economic status and related contextual factors may play an important role in cognitive enhancement acceptance and should be considered in future research. We further highlight that our studies only investigated German-speaking samples, and that attitudes towards enhancement might differ between cultures. We also address potential self-report bias and hypothetical responding in the limitations section:

"Furthermore, the present samples primarily consisted of young, well-educated, German-speaking individuals from Austria and Germany, which limits the generalizability of our findings. Future studies should aim to include more diverse samples, particularly with respect to socio-economic status, which is associated not only with differential access to enhancement technologies but also with learning motivation [104]—factors that might shape enhancement acceptance. Should cognitive enhancement methods become more effective in the future, unequal access could further amplify existing disparities in cognitive performance and related outcomes. Attitudes toward enhancement may also differ across cultures, making cross-cultural replications an important next step. Beyond sample characteristics, further methodological limitations—typical for questionnaire studies of this sort—warrant consideration. For instance, acceptance was assessed via self-report, which is susceptible to social desirability bias, particularly given that some enhancement methods might carry stigma."

(11) The discussion could engage more directly with ethical and societal implications of enhancement acceptance, especially regarding inequality, coercion, and accessibility, which are mentioned in the introduction but less integrated into the interpretation of findings.

Answer: Thank you for this important comment. We tried to further deepen these associations in our discussion, also with regard to our qualitative findings. Our main discussion points are that these ethical and societal themes were surprisingly infrequently named as motives for the rejection or acceptance of enhancement and may therefore be less salient when deciding whether one would use enhancement oneself (see discussion section "Acceptance of cognitive enhancement methods"). Further, we conclude that the socio-economic status of a person should be considered in future research, as a higher socio-economic status might not only be tied to better accessibility, but also to already higher learning motivation (see p. 60). We hope this helps to deepen the interpretation of our results considering the ethical and societal issues raised.

However, we also want to refer to other publications (Bostrom & Sandberg, 2009; Neubauer, 2021; Neubauer & Wood, 2022), which specifically adopt a more philosophical viewpoint and further elaborate concerns regarding ethical and societal implications. A deep dive into this topic would be beyond the scope of the present research paper.

(12) Figure and table presentation is generally clear, although some large correlation tables could potentially be moved entirely to supplementary materials to improve readability of the main text.

Answer: Thank you for this suggestion. After careful consideration, we decided not to move additional tables to the supporting information. These correlation tables, in our view, contain results that are central to the contribution of the manuscript. In particular, they provide a compact overview of the pattern of associations and, in addition to the correlation coefficients, also include bootstrapped CIs and Bayes factors, which we consider important for enabling readers to evaluate the robustness and evidential strength of the findings.

We therefore prefer to keep these tables in the main text, while relying on the supporting information for additional robustness checks. We hope this rationale is sufficient and that the current presentation strikes an acceptable balance between readability and transparency.

(13) The manuscript is generally carefully prepared and transparent, particularly regarding preregistration and open-science practices, which constitutes a significant strength of the work.

Answer: Thank you very much. We are glad that our commitment to open science practices was noted and appreciated.
