# Supplementary material for: Individual differences and motives for the acceptance of cognitive enhancement: A mixed-methods investigation
Source: PLoS One. 2026 Jul 10;21(7):e0353234. doi: 10.1371/journal.pone.0353234 (PMC13354088; doi:10.1371/journal.pone.0353234)
Supplement: Peer review history

# Peer review: Decision Letter 0

**DOI:** 10.1371/journal.pone.0353234.r001

**Authors:** Peter Karl Jonason

11 May 2026

PONE-D-25-63419

Individual differences and motives for the acceptance of cognitive enhancement: A mixed-methods investigation

PLOS One

Dear Dr. Kohlmeier,

Thank you for submitting your manuscript to PLOS ONE and apologies the lengthy process this took. After careful consideration, we feel that it has merit but does not fully meet PLOS ONE’s publication criteria as it currently stands. Therefore, we invite you to submit a revised version of the manuscript that addresses the points raised during the review process.

One reviewer has already cautioned you on matters that still persist. While they did not specify, I suspect you know what these are and could at least attempt to address them here. The other reviewer had issue with conceptual, methodological, and statistical clarity.

Please submit your revised manuscript by Jun 25 2026 11:59PM. If you will need more time than this to complete your revisions, please reply to this message or contact the journal office at plosone@plos.org. When you’re ready to submit your revision, log on to https://www.editorialmanager.com/pone/ and select the ‘Submissions Needing Revision’ folder to locate your manuscript file.

Please include the following items when submitting your revised manuscript:

- A letter that responds to each point raised by the academic editor and reviewer(s). You should upload this letter as a separate file labeled ‘Response to Reviewers’.
- A marked-up copy of your manuscript that highlights changes made to the original version. You should upload this as a separate file labeled ‘Revised Manuscript with Track Changes’.
- An unmarked version of your revised paper without tracked changes. You should upload this as a separate file labeled ‘Manuscript’.

If you would like to make changes to your financial disclosure, please include your updated statement in your cover letter. Guidelines for resubmitting your figure files are available below the reviewer comments at the end of this letter.

If applicable, we recommend that you deposit your laboratory protocols in protocols.io to enhance the reproducibility of your results. Protocols.io assigns your protocol its own identifier (DOI) so that it can be cited independently in the future. For instructions see: https://journals.plos.org/plosone/s/submission-guidelines#loc-laboratory-protocols. Additionally, PLOS ONE offers an option for publishing peer-reviewed Lab Protocol articles, which describe protocols hosted on protocols.io. Read more information on sharing protocols at https://plos.org/protocols?utm_medium=editorial-email&utm_source=authorletters&utm_campaign=protocols.

As the corresponding author, your ORCID iD is verified in the submission system and will appear in the published article. PLOS supports the use of ORCID, and we encourage all coauthors to register for an ORCID iD and use it as well. Please encourage your coauthors to verify their ORCID iD within the submission system before final acceptance, as unverified ORCID iDs will not appear in the published article. *Only*  the individual author can complete the verification step; PLOS staff *cannot*  verify ORCID iDs on behalf of authors.

We look forward to receiving your revised manuscript.

Kind regards,

Peter Karl Jonason

Academic Editor

PLOS One

Journal Requirements:

When submitting your revision, we need you to address these additional requirements.

1. Please ensure that your manuscript meets PLOS ONE’s style requirements, including those for file naming. The PLOS ONE style templates can be found at https://journals.plos.org/plosone/s/file?id=wjVg/PLOSOne_formatting_sample_main_body.pdf and https://journals.plos.org/plosone/s/file?id=ba62/PLOSOne_formatting_sample_title_authors_affiliations.pdf

2. Thank you for stating in your Funding Statement:

[This research was supported by two financial grants awarded by the NAWI Faculty of the University of Graz (https://nawi.uni-graz.at/en/), aimed at supporting students in conducting research activities. The funding was provided to support data collection for Study 1 and Study 2. The funders had no role in the study design, data collection and analysis, decision to publish, or preparation of the manuscript.].

Please provide an amended statement that declares *all* the funding or sources of support (whether external or internal to your organization) received during this study, as detailed online in our guide for authors at http://journals.plos.org/plosone/s/submit-now.  Please also include the statement “There was no additional external funding received for this study.” in your updated Funding Statement.

Please include your amended Funding Statement within your cover letter. We will change the online submission form on your behalf.

3. Please remove any funding-related text from the manuscript. Funding information should not appear in any section or other areas of your manuscript. We will only publish funding information present in the Funding Statement section of the online submission form.

4. Please include your full ethics statement in the ‘Methods’ section of your manuscript file. In your statement, please include the full name of the IRB or ethics committee who approved or waived your study, as well as whether or not you obtained informed written or verbal consent. If consent was waived for your study, please include this information in your statement as well.

If the reviewer comments include a recommendation to cite specific previously published works, please review and evaluate these publications to determine whether they are relevant and should be cited. There is no requirement to cite these works unless the editor has indicated otherwise.

[Note: HTML markup is below. Please do not edit.]

Reviewers’ comments:

Reviewer’s Responses to Questions

**Comments to the Author**

1. Is the manuscript technically sound, and do the data support the conclusions?

The manuscript must describe a technically sound piece of scientific research with data that supports the conclusions. Experiments must have been conducted rigorously, with appropriate controls, replication, and sample sizes. The conclusions must be drawn appropriately based on the data presented.

Reviewer #1: Partly

Reviewer #2: Yes

**********

2. Has the statistical analysis been performed appropriately and rigorously?

Reviewer #1: I Don’t Know

Reviewer #2: Yes

**********

3. Have the authors made all data underlying the findings in their manuscript fully available?

The PLOS Data policy requires authors to make all data underlying the findings described in their manuscript fully available without restriction, with rare exception (please refer to the Data Availability Statement in the manuscript PDF file). The data should be provided as part of the manuscript or its supporting information, or deposited to a public repository. For example, in addition to summary statistics, the data points behind means, medians and variance measures should be available. If there are restrictions on publicly sharing data—e.g. participant privacy or use of data from a third party—those must be specified.

Reviewer #1: Yes

Reviewer #2: Yes

**********

4. Is the manuscript presented in an intelligible fashion and written in standard English?

PLOS ONE does not copyedit accepted manuscripts, so the language in submitted articles must be clear, correct, and unambiguous. Any typographical or grammatical errors should be corrected at revision, so please note any specific errors here.

Reviewer #1: Yes

Reviewer #2: Yes

**********

5. Review Comments to the Author

Please use the space provided to explain your answers to the questions above. You may also include additional comments for the author, including concerns about dual publication, research ethics, or publication ethics. (Please upload your review as an attachment if it exceeds 20,000 characters)

Reviewer #1: This looks like an interesting study, the authors have carried out earlier research on the topic and I’ve reviewed their work before.

However, as I’ve also told them before, the way they present cognitive enhancement as something new is inaccurate. For example, the references 3 and 4 (line 61) seem odd as evidence that enhancement is "readily applied". There are comprehensive surveys with representative data and even recent books addressing this issue in more detail, also in its social and historical dimension.

Time is scarce. I don’t have enough time to give these authors the same suggestions over and over again. Good luck with your projects!

Reviewer #2: Individual differences and motives for the acceptance of cognitive enhancement: A mixed-methods investigation

The manuscript investigates the acceptance of cognitive enhancement methods and the individual differences associated with this acceptance. The topic is timely and potentially relevant given the increasing societal interest in enhancement technologies. The inclusion of two preregistered studies, the combination of quantitative and qualitative approaches, and the open-science practices (preregistration, OSF data/code availability) are important strengths of the work. The manuscript is generally well written and theoretically informed. The distinction between active and passive enhancement methods is meaningful and appears empirically supported across both studies. However, the paper also presents several conceptual and methodological weaknesses that should be addressed before publication. In particular, the theoretical framework remains somewhat diffuse, many hypotheses are weakly justified, the manuscript includes a very large number of correlational analyses with relatively small effects, and the interpretation of findings occasionally exceeds the evidential strength of the data. Overall, the manuscript has potential, but substantial revision would improve its clarity, theoretical coherence, and interpretative rigor. I have the following comments:

(1) Theoretical Framework Is Overly Broad and Fragmented. The manuscript combines: intelligence, self-estimated intelligence, Big Five traits, Dark Triad traits, anxiety, novelty seeking, self-esteem, vocational interests, science-fiction hobbyism, and qualitative motives within a single framework. While each variable is individually motivated, the overall theoretical integration is weak. The manuscript often reads as a collection of loosely connected predictors rather than a coherent explanatory model of enhancement acceptance. For example: the rationale linking intelligence to enhancement acceptance remains speculative, novelty seeking is only briefly justified, and several hypotheses appear exploratory despite formal preregistration. The paper would benefit from: a clearer overarching conceptual framework, stronger prioritization of hypotheses, and a sharper distinction between confirmatory and exploratory analyses. Currently, the study risks appearing “kitchen sink”-like, where many variables are tested simultaneously without a sufficiently integrated theoretical rationale.

(2) Multiple Testing and Risk of False Positives. The manuscript conducts a very large number of correlations and regressions across two studies. Although preregistration is commendable, the statistical strategy still raises concerns: dozens of bivariate correlations, multiple regression models, subgroup analyses, exploratory analyses, and qualitative categorizations. Most reported effects are relatively small (e.g., r ≈ .15–.30). In this context, the manuscript should discuss: multiplicity concerns, robustness of effects, and the possibility that some findings may represent chance associations. Importantly, some findings are inconsistent across studies: intelligence predicts active enhancement in Study 1 but only partially in Study 2, conscientiousness effects vary, several personality effects disappear.

The manuscript currently emphasizes statistically significant findings while underemphasizing inconsistencies.

(3) Interpretation of Effect Sizes Is Sometimes Overstated. Several statistically significant findings correspond to relatively modest effect sizes. For example: intelligence correlations around r = .19, investigative interests around r = .18–.26, science-fiction hobbyism around r = .23–.31. These are meaningful but small-to-moderate associations. However, parts of the discussion occasionally imply stronger psychological importance than warranted. The authors should adopt more cautious language and avoid implying that these traits are major determinants of enhancement acceptance.

(4) Conceptual Ambiguity of “Acceptance”. The manuscript defines acceptance as “willingness to make use” of enhancement methods. However, this construct remains somewhat ambiguous: Is it a moral attitude? A behavioral intention? A technological openness trait? A risk-benefit evaluation? Mere curiosity? Especially because many enhancement methods described are highly speculative (e.g., genetic enhancement, brain-machine interfaces), participants’ responses may reflect: science-fiction imagination, perceived realism, novelty, or media exposure, rather than genuine behavioral willingness. The manuscript should discuss more explicitly the ecological validity and interpretative limits of these acceptance measures.

(5) Factor Structure Requires More Critical Discussion. The active/passive distinction is interesting and appears replicated. However: the factor solution explains only 42.49% of variance, some loadings are moderate, and the conceptual classification is debatable. For example: neurofeedback contains both active and passive components, brain-machine interfaces arguably involve extensive training, current-based stimulation may vary in user involvement. The manuscript currently treats the two-factor structure as more definitive than warranted.

(6) Qualitative Component Is Underdeveloped. The qualitative analyses are potentially valuable but appear secondary and insufficiently integrated into the manuscript’s theoretical contribution. The paper would benefit from: a clearer methodological description of coding procedures, intercoder reliability information, richer illustrative quotations, and deeper integration between qualitative and quantitative findings. At present, the mixed-methods approach feels somewhat additive rather than genuinely integrative.

I also have the following minor comments:

(1) Some sections of the introduction are overly long and could be streamlined. For example, the detailed descriptions of enhancement technologies sometimes resemble review material rather than directly supporting the research questions.

(2) The manuscript occasionally uses speculative or rhetorical language: “replacement of humans by AI,” “superintelligence,” “designer babies,” etc.

These passages could be toned down in a scientific article.

(3) The term “acceptance” is sometimes conflated with actual future behavior.

(4) Some Cronbach’s alpha values are relatively low, especially psychopathy (α = .55). This limitation should be discussed more explicitly.

(5) The manuscript would benefit from a stronger distinction between preregistered confirmatory analyses and exploratory follow-up analyses. At several points, exploratory findings are discussed with a level of emphasis similar to preregistered hypotheses.

(6) Some terminology should be standardized throughout the manuscript. For example, the paper alternates between: “cognitive enhancement,” “enhancement,” “enhancement methods,” and “acceptance of enhancement,” which occasionally reduces conceptual precision.

(7) The reporting of Bayes Factors is appreciated, but their interpretation is sometimes minimal. The manuscript could briefly clarify how evidence categories were interpreted (e.g., anecdotal, moderate, strong evidence).

(8) The rationale for excluding the “mind upload” vignette from the original questionnaire could be discussed more critically, since perceived realism may itself be psychologically informative.

(9) Some regression models explain relatively modest proportions of variance (e.g., 13–18%), yet the discussion occasionally implies broader explanatory power than warranted.

(10) The manuscript would benefit from a short limitations paragraph discussing: self-report bias, social desirability, hypothetical responding, and the use of predominantly educated European samples.

(11) The discussion could engage more directly with ethical and societal implications of enhancement acceptance, especially regarding inequality, coercion, and accessibility, which are mentioned in the introduction but less integrated into the interpretation of findings.

(12) Figure and table presentation is generally clear, although some large correlation tables could potentially be moved entirely to supplementary materials to improve readability of the main text.

(13) The manuscript is generally carefully prepared and transparent, particularly regarding preregistration and open-science practices, which constitutes a significant strength of the work.

**********

6. PLOS authors have the option to publish the peer review history of their article (what does this mean?). If published, this will include your full peer review and any attached files.

If you choose “no”, your identity will remain anonymous but your review may still be made public.

**Do you want your identity to be public for this peer review?**  For information about this choice, including consent withdrawal, please see our Privacy Policy.

Reviewer #1: No

Reviewer #2: **Yes:** P Fuster-Parra

**********

[NOTE: If reviewer comments were submitted as an attachment file, they will be attached to this email and accessible via the submission site. Please log into your account, locate the manuscript record, and check for the action link "View Attachments". If this link does not appear, there are no attachment files.]

To ensure your figures meet our technical requirements, please review our figure guidelines: https://journals.plos.org/plosone/s/figures

You may also use PLOS’s free figure tool, NAAS, to help you prepare publication quality figures: https://journals.plos.org/plosone/s/figures#loc-tools-for-figure-preparation.

NAAS will assess whether your figures meet our technical requirements by comparing each figure against our figure specifications.

Attachment Submitted filename: IndividualPLOS.pdf

# Author comment: Author response to Decision Letter 1

**DOI:** 10.1371/journal.pone.0353234.r002

18 Jun 2026

Reply to the Editor:

Thank you for submitting your manuscript to PLOS ONE and apologies the lengthy process this took. After careful consideration, we feel that it has merit but does not fully meet PLOS ONE’s publication criteria as it currently stands. Therefore, we invite you to submit a revised version of the manuscript that addresses the points raised during the review process.

Answer: We thank you for your efforts in coordinating the review process and welcome the opportunity to revise our manuscript in light of your comments and those of the reviewers. Please find our point-by-point responses below, along with the corresponding changes made to the manuscript. We believe these revisions have substantially strengthened our work and hope that it is now suitable for publication in PLOS ONE.

One reviewer has already cautioned you on matters that still persist. While they did not specify, I suspect you know what these are and could at least attempt to address them here. The other reviewer had issue with conceptual, methodological, and statistical clarity.

Answer: Although we cannot determine with certainty what Reviewer 1 was referring to, we believe we have addressed their concern by explicitly acknowledging that the concept of cognitive enhancement has a long history, with discussions dating back centuries (see also our response to Reviewer 1). We are also confident that the conceptual, methodological, and statistical concerns raised by Reviewer 2 have been resolved.

Reply to Reviewer 1:

This looks like an interesting study, the authors have carried out earlier research on the topic and I’ve reviewed their work before.

However, as I’ve also told them before, the way they present cognitive enhancement as something new is inaccurate. For example, the references 3 and 4 (line 61) seem odd as evidence that enhancement is “readily applied”. There are comprehensive surveys with representative data and even recent books addressing this issue in more detail, also in its social and historical dimension. Time is scarce. I don’t have enough time to give these authors the same suggestions over and over again. Good luck with your projects!

Answer: Thank you for your review and your repeated engagement with our work on cognitive enhancement.

In response to your concerns, we have revised several parts of the abstract (see p. 2) and introduction (see p. 3 and 12) to better acknowledge the long history and broader social and historical context of cognitive enhancement. Specifically, we now (a) more clearly describe cognitive enhancement as embedded in a longer-standing discourse, (b) cite additional relevant work that provides a more comprehensive historical and sociological perspective on enhancement (e.g., Schleim, 2023; Schleim & Quednow, 2018), and (c) aim to provide a more nuanced view on the prevalence of cognitive enhancement use.

At the same time, we believe that new technologies, social media influences (including emerging "neuroenhancement bubbles" and self improvement trends), and the rapid diffusion of biomedical methods (e.g., GLP 1 agonist hype) are currently reshaping the enhancement landscape. These developments might accelerate the wider use of cognitive enhancement methods.

Reply to Reviewer 2:

The manuscript investigates the acceptance of cognitive enhancement methods and the individual differences associated with this acceptance. The topic is timely and potentially relevant given the increasing societal interest in enhancement technologies. The inclusion of two preregistered studies, the combination of quantitative and qualitative approaches, and the open-science practices (preregistration, OSF data/code availability) are important strengths of the work. The manuscript is generally well written and theoretically informed. The distinction between active and passive enhancement methods is meaningful and appears empirically supported across both studies. However, the paper also presents several conceptual and methodological weaknesses that should be addressed before publication. In particular, the theoretical framework remains somewhat diffuse, many hypotheses are weakly justified, the manuscript includes a very large number of correlational analyses with relatively small effects, and the interpretation of findings occasionally exceeds the evidential strength of the data. Overall, the manuscript has potential, but substantial revision would improve its clarity, theoretical coherence, and interpretative rigor. I have the following comments:

Answer: We are grateful for your thorough review of our manuscript. We were pleased to hear that you consider the research timely and that our open science practices were well received. Your comments have been carefully considered, and we have revised the manuscript accordingly. Most importantly, we are now more thoroughly explaining the exploratory nature of our research and have carefully revised the interpretation of our findings. Please see our point-by-point responses below.

(1) Theoretical Framework Is Overly Broad and Fragmented. The manuscript combines: intelligence, self-estimated intelligence, Big Five traits, Dark Triad traits, anxiety, novelty seeking, self-esteem, vocational interests, science-fiction hobbyism, and qualitative motives within a single framework. While each variable is individually motivated, the overall theoretical integration is weak. The manuscript often reads as a collection of loosely connected predictors rather than a coherent explanatory model of enhancement acceptance. For example: the rationale linking intelligence to enhancement acceptance remains speculative, novelty seeking is only briefly justified, and several hypotheses appear exploratory despite formal preregistration. The paper would benefit from: a clearer overarching conceptual framework, stronger prioritization of hypotheses, and a sharper distinction between confirmatory and exploratory analyses. Currently, the study risks appearing “kitchen sink”-like, where many variables are tested simultaneously without a sufficiently integrated theoretical rationale.

Answer: Thank you very much for this thoughtful and constructive comment. We appreciate the opportunity to clarify our theoretical rationale.

Our overarching aim with this work was to provide an initial, systematic mapping of individual-difference correlates of cognitive enhancement acceptance. Because the literature on psychological predictors of cognitive enhancement acceptance is still emerging, often inconsistent and mostly focused on single cognitive enhancement methods, we deliberately adopted an exploratory approach. This approach combined (a) variables that have been conceptually or empirically linked to enhancement in previous work (e.g., personality traits, intelligence and science-fiction hobbyism) and (b) less frequently, but conceptually plausible, examined predictors (e.g., novelty seeking and trait anxiety). Our intention was to establish a broader empirical foundation on which more focused theoretical models can be built. We therefore refrained from constructing a post hoc explanatory framework, keeping the exploratory rationale closely aligned with our preregistered research questions and hypotheses. We clarified this aim of our study on p. 11 of the introduction:

“Research on the psychological predictors of cognitive enhancement acceptance is still emerging, often inconsistent, and mostly focused on single enhancement methods. The present study therefore examines a range of individual difference variables: those conceptually or empirically linked to cognitive enhancement acceptance in previous work, such as personality traits, intelligence, and science-fiction hobbyism, as well as less frequently examined but conceptually plausible predictors such as novelty seeking and trait anxiety. The goal is to establish a broader empirical foundation from which more focused theoretical models on the predictors of cognitive enhancement acceptance can be developed in the future.”

Furthermore, we now more explicitly elaborate on why we tested intelligence and novelty seeking as predictors. See p.8 and 10:

“First, we aimed at taking a closer look at intelligence and self-estimated intelligence, as cognitive enhancement methods explicitly target cognitive abilities, like intelligence. For this link, two hypotheses have been proposed [9,40]: the rich-get-richer hypothesis and the compensation hypothesis. The rich-get-richer hypothesis proposes that individuals with high cognitive abilities would benefit more from cognitive enhancement and might also be more drawn towards cognitive enhancement (see also ‘Matthew effect’ [41]). Their cognitive abilities would increase, further distinguishing them from individuals with lower cognitive abilities. The compensation hypothesis proposes the opposite effect. People with lower cognitive abilities would benefit more from cognitive enhancement and might be more drawn towards it, resulting in an equalizing effect. This would alter the distribution of intelligence in the population, rendering cognitive ability less important due to the reduced variance [40] (see also reverse Matthew effect [42]).”

“Furthermore, in this study we also tested novelty seeking as a potential predictor of the acceptance of cognitive enhancement methods. A study from Maier et al. [53] found a positive relationship between novelty seeking and the use of pharmacological enhancement and people with high levels of novelty seeking may be early adopters of new technologies [54]. They further show a higher risk preference [55], and higher delay discounting (i.e., a higher preference for a smaller, immediate reward over a bigger, delayed rewards [56]), potentially indicating a positive relationship with the acceptance of (specifically passive) cognitive enhancement methods.”

Taken together, we agree that a more elaborate, tightly integrated theoretical framework would be valuable and is a key next step for the field. At the same time, we believe that mapping out which individual characteristics (e.g., personality, intelligence, interests) do or do not relate to cognitive enhancement acceptance is a necessary foundation for such a framework. As stated in our Discussion, we describe how our results suggest that individual differences alone may not be sufficient to explain cognitive enhancement acceptance, and we explicitly call for future work that integrates these characteristics with situational and contextual factors. In our revised manuscript, we further elaborate on this need for a more comprehensive theoretical framework and clarify how our findings can inform it.

Finally, we now more clearly distinguish our analyses between confirmatory, planned exploratory, and follow-up exploratory analyses in line with our preregistrations in the result sections of our manuscript. For more on this, please see our response to your minor comment nr. 5.

(2) Multiple Testing and Risk of False Positives. The manuscript conducts a very large number of correlations and regressions across two studies. Although preregistration is commendable, the statistical strategy still raises concerns: dozens of bivariate correlations, multiple regression models, subgroup analyses, exploratory analyses, and qualitative categorizations. Most reported effects are relatively small (e.g., r ≈ .15–.30). In this context, the manuscript should discuss: multiplicity concerns, robustness of effects, and the possibility that some findings may represent chance associations. Importantly, some findings are inconsistent across studies: intelligence predicts active enhancement in Study 1 but only partially in Study 2, conscientiousness effects vary, several personality effects disappear.

The manuscript currently emphasizes statistically significant findings while underemphasizing inconsistencies.

Answer: Thank you for raising this important point. We agree that the large number of correlations and regression analyses, combined with relatively small effect sizes, raises concerns about multiplicity and the robustness of individual findings.

We now apply exploratory Bonferroni–Holm corrections to selected sets of correlations (see Supporting Material Tables S5 and S8) so that readers can better judge the robustness of these associations under stricter multiple-testing control. More specifically, we applied corrections when variables shared the same measurement instrument, such as Big Five, Dark Triad, and interest questionnaires. Under this correction, some associations with enhancement and acceptance (e.g., with Machiavellianism and narcissism in Study 1) appear relatively robust, whereas others (e.g., with Conscientiousness in Study 1 and its sub-facet Dutifulness in Study 2) are less stable and should be interpreted more cautiously.

Importantly, to follow our preregistered analysis plan, we continued to select predictors for the regression models based on uncorrected bivariate significance. We chose this approach to avoid an overly conservative procedure that could substantially increase Type II error and obscure potentially meaningful, but small, effects, particularly given the mostly exploratory nature of our research questions. However, the possibility of chance findings can, of course, not be fully ruled out. We therefore added a limitations paragraph explicitly addressing these issues (see p. 60):

“Critically, across both studies, we conducted a large number of correlation and regression analyses, thus, chance findings cannot be ruled out. Moreover, some measures (e.g., intelligence and personality instruments) differed between the two studies (which is favorable when aiming at generalizable claims), and some predictors—most notably the psychopathy scale in Study 1—showed relatively low internal consistencies. These issues may have contributed to inconsistencies across studies and should be taken into account when interpreting the results. We therefore place particular emphasis on associations that were observed in both studies and are in line with previous research (e.g., the moderate association of cognitive enhancement acceptance and science-fiction hobbyism [9]).”

We also highlight that some measures (e.g., the intelligence and personality assessments) differed between Studies 1 and 2 (which is an advantage when aiming at generalizing claims), and that single predictors—most notably the psychopathy scale in Study 1—showed relatively low internal consistency. We acknowledge that these measurement issues may have contributed to inconsistencies across studies and that some effects might be due to random variation. Accordingly, across our whole manuscript, we now emphasize more clearly that the observed effects are generally small and, in some cases, inconclusive (e.g., associations found only in one of the two studies). Most importantly, we place stronger interpretative weight on those associations that were observed consistently across both studies and align with previous work (e.g., the moderate association of cognitive enhancement acceptance and science-fiction hobbyism).

(3) Interpretation of Effect Sizes Is Sometimes Overstated. Several statistically significant findings correspond to relatively modest effect sizes. For example: intelligence correlations around r = .19, investigative interests around r = .18–.26, science-fiction hobbyism around r = .23–.31. These are meaningful but small-to-moderate associations. However, parts of the discussion occasionally imply stronger psychological importance than warranted. The authors should adopt more cautious language and avoid implying that these traits are major determinants of enhancement acceptance.

Answer: In the revised manuscript, we have adopted more cautious language when interpreting our findings and now more explicitly acknowledge that the observed effect sizes are mostly small to moderate. For instance, we now also explicitly mention this in the abstract (see p. 2). Additionally, we also place stronger interpretative weight on those associations that were observed consistently across both studies and that align with previous work (e.g., the moderate association of cognitive enhancement acceptance and science-fiction hobbyism). For more on this, please see our response to your major comment nr. 2.

Attachment Submitted filename: Response to Reviewers.pdf

# Editor report: Decision Letter 1

**DOI:** 10.1371/journal.pone.0353234.r003

**Authors:** Peter Karl Jonason

21 Jun 2026

Individual differences and motives for the acceptance of cognitive enhancement: A mixed-methods investigation

PONE-D-25-63419R1

Dear Dr. Kohlmeier,

We’re pleased to inform you that your manuscript has been judged scientifically suitable for publication and will be formally accepted for publication once it meets all outstanding technical requirements.

Within one week, you’ll receive an e-mail detailing the required amendments. When these have been addressed, you’ll receive a formal acceptance letter and your manuscript will be scheduled for publication.

An invoice will be generated when your article is formally accepted. Please note, if your institution has a publishing partnership with PLOS and your article meets the relevant criteria, all or part of your publication costs will be covered. Please make sure your user information is up-to-date by logging into Editorial Manager at Editorial Manager® and clicking the ‘Update My Information’ link at the top of the page. For questions related to billing, please contact billing support.

If your institution or institutions have a press office, please notify them about your upcoming paper to help maximize its impact. If they’ll be preparing press materials, please inform our press team as soon as possible -- no later than 48 hours after receiving the formal acceptance. Your manuscript will remain under strict press embargo until 2 pm Eastern Time on the date of publication. For more information, please contact onepress@plos.org.

Kind regards,

Peter Karl Jonason

Academic Editor

PLOS One

Additional Editor Comments (optional):

Reviewers’ comments:

# Editor report: Acceptance letter

**DOI:** 10.1371/journal.pone.0353234.r004

**Authors:** Peter Karl Jonason

PONE-D-25-63419R1

PLOS One

Dear Dr. Kohlmeier,

I’m pleased to inform you that your manuscript has been deemed suitable for publication in PLOS One. Congratulations! Your manuscript is now being handed over to our production team.

At this stage, our production department will prepare your paper for publication. This includes ensuring the following:

* All references, tables, and figures are properly cited

* All relevant supporting information is included in the manuscript submission,

* There are no issues that prevent the paper from being properly typeset

You will receive further instructions from the production team, including instructions on how to review your proof when it is ready. Please keep in mind that we are working through a large volume of accepted articles, so please give us a few days to review your paper and let you know the next and final steps.

Lastly, if your institution or institutions have a press office, please let them know about your upcoming paper now to help maximize its impact. If they’ll be preparing press materials, please inform our press team within the next 48 hours. Your manuscript will remain under strict press embargo until 2 pm Eastern Time on the date of publication. For more information, please contact onepress@plos.org.

You will receive an invoice from PLOS for your publication fee after your manuscript has reached the completed accept phase. If you receive an email requesting payment before acceptance or for any other service, this may be a phishing scheme. Learn how to identify phishing emails and protect your accounts at https://explore.plos.org/phishing.

If we can help with anything else, please email us at customercare@plos.org.

Thank you for submitting your work to PLOS ONE and supporting open access.

Kind regards,

PLOS ONE Editorial Office Staff

on behalf of

Dr. Peter Karl Jonason

Academic Editor

PLOS One
